# Circular RNA circDhx32 promotes cardiac inflammatory responses in mouse cardiac ischemia-reperfusion injury via binding to FOXO1 competed with AdipoR1

**DOI:** 10.1038/s41401-025-01593-9

**Published:** 2025-06-17

**Authors:** Wei Si, Chun-lei Wang, Ling-hua Zeng, Qiao-yue Zhao, Ya-ting Xie, Yang Yang, Hong-tao Diao, Jing-lun Song, Han Wu, Feng Zhang, Zhuo Wang, Xue Kong, Wei-tao Jiang, Xin-yue Zhang, Ke-ying Lin, Fang-ting Yao, Yu-ting Xiong, Teng-fei Pan, Ping Pang, Bao-feng Yang, Yu Bian

**Affiliations:** 1 Department of Pharmacology, the State-Province Key Laboratories of Biomedicine-Pharmaceutics of China, Key Laboratory of Cardiovascular Research, Ministry of Education, College of Pharmacy, Harbin Medical University, Harbin, 150081 China; 2 Shanghai Frontiers Science Research Center for Druggability of Cardiovascular noncoding RNA, Institute for Frontier Medical Technology, Shanghai University of Engineering Science, Shanghai, 201620 China

**Keywords:** ischaemic cardiac disease, ischaemia‒reperfusion injury, CircDhx32, FOXO1, AdipoR1, inflammation

## Abstract

Ischaemic heart disease is an important cause of death in humans, and resupply of blood to damaged myocardium can exacerbate the risk of cardiac I/R injury. Circular RNAs (circRNAs) play an important role in cardiovascular disease. In this study we investigated the regulatory role of circDhx32 in the progression of I/R injury. Cardiac I/R model was established in mice by ligating the left anterior descending coronary artery (LAD) for 45 min, followed by blood reperfusion for 24 h or 2 weeks. For in vitro study, neonatal mouse ventricular cardiomyocytes were subjected to hypoxia-reoxygenation (H/R) assault. CircDhx32 was significantly upregulated in I/R-treated mice and H/R-treated cardiomyocytes. Cardiomyocyte-specific knockdown of circDhx32 ameliorated the pathological outcomes of cardiac I/R injury including improved cardiac function, reduced infarct size and reduced release of cardiac injury biomarkers. The protective effects of circDhx32 silencing were also observed in cardiomyocytes after H/R. We demonstrated that ALKBH5 functioned as an m^6^A demethylase, removing the m^6^A modification sites of circDhx32. Reduced m^6^A modification inhibited recognition and binding by the m^6^A readers YTHDF2 and YTHDC1, leading to circDhx32 degradation and diminished nucleoplasmic export under pathological conditions. Elevated circDhx32 inhibited the transcriptional activation of AdipoR1 by binding to FOXO1. Conversely, circDhx32 deficiency alleviated the inflammatory responses in I/R-treated mice and H/R-treated cardiomyocytes including decreased mRNA expression levels and release of inflammatory cytokines such as IL-6, TNF-α and IL-1β potentially through modulation of the AdipoR1-AMPK-NF-κB signaling pathway. In conclusion, ALKBH5 acted as m^6^A eraser accompanied by the m^6^A readers YTHDF2 and YTHDC1 to promote high expression and nuclear retention of circDhx32 under pathological conditions. CircDhx32 regulated the inflammatory responses to cardiac I/R injury by targeting the AdipoR1-AMPK-NF-κB signaling pathway, which competed with AdipoR1 for FOXO1. These results reveal a novel mechanism underlying cardiac ischaemic injury, and circDhx32 is expected to be a potential therapeutic target for early intervention in ischaemic cardiac disease.

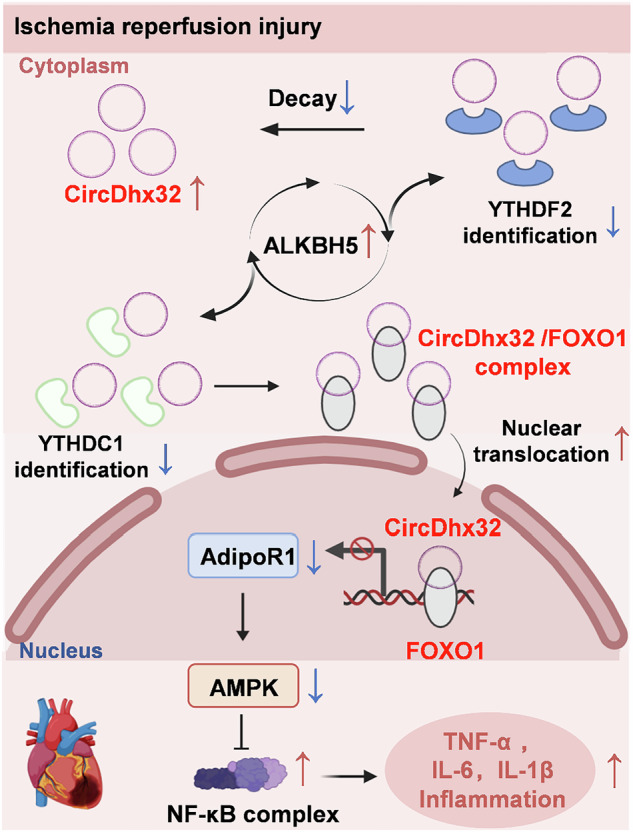

## Introduction

Ischaemic heart disease is an important cause of death in humans, and resupply of blood to damaged myocardium can ameliorate the myocardial infarct size while exacerbating the risk of cardiac I/R injury [1, 2]. Accumulating evidence indicates that the cell inflammatory response is of paramount importance in cardiac I/R injury [1, 3–5]. The inhibition of CaMKII-δ9 markedly restrains cardiac NF-κB activation and the inflammatory response and subsequently ameliorates myocardial I/R injury [6]. However, specific therapies to mitigate reperfusion injury remain unvalidated [5, 7]. Hence, exploring the molecular mechanisms of cardiac I/R injury and finding novel therapeutic targets are highly clinically important.

CircRNAs are formed by backsplicing of primary transcripts and lack 5′ to 3′ polarity as well as a polyadenylation tail, have high stability and are evolutionarily conserved [8–11]. CircRNAs can serve as microRNA sponges and protein scaffolds and influence protein expression [12–14]. These molecules play crucial roles in various cardiovascular diseases; for example, overexpression of circSQSTM1, which restrains inflammation and oxidative stress by directly binding with eIF4A3, attenuates the progression of atherosclerosis [15]. Wang et al. reported that circRYK increases the stability of VLDLR mRNA via the RNA-binding protein HuR, driving glioblastoma progression [16]. N6-methyladenosine (m^6^A) modification is the most extensive modification in RNA, and previous studies reported that m^6^A dysregulation of circRNAs substantially accelerates the development and progression of various diseases [17, 18]. For example, the m^6^A reader IGF2BP1 can identify and bind to the m^6^A modification sites of circMDK to increase its transcriptomic stability, thereby promoting the progression of hepatocellular carcinoma [19], whereas circ0008399 binds to WTAP to promote the formation of the WTAP/METTL3/METTL14 m^6^A methyltransferase complex, increasing its RNA stability in an m^6^A-dependent manner, which provides potential therapeutic targets for bladder cancer [20]. m^6^A-modified circSAV1 recruits YTHDF1 to promote IREB2 translation, thus contributing to ferroptosis in chronic obstructive pulmonary disease [21]. However, the regulatory relationships between circRNAs and cardiac I/R injury are still not entirely clear, and the role of circRNAs in cardiac I/R injury requires further elucidation.

Forkhead box O1 (FOXO1) is a conserved cross-species transcription factor encoded by the FKHR gene that has a major effect on transcription, metabolism and coding of target genes, thereby modulating cellular metabolism and death, oxidative stress and tissue remodeling [22–24]. FOXO1 is shown to promote the transcription of VCAM-1 and ICAM-1 to mediate the endothelial cell inflammatory response in atherosclerosis [25]. Additionally, the activation of FOXO1 stimulates NOX4 expression via the upregulation of KLF5 expression, which causes cardiac dysfunction and oxidative stress in diabetic cardiomyopathy [26]. There is reliable evidence that FOXO1 targets adiponectin receptor protein 1 (AdipoR1) to affect cardiovascular disease, and Cui et al. found that FOXO1 regulates the transcription of AdipoR1 in cardiomyocytes by binding directly to the AdipoR1 promoter sequence from -167 to -157 bp [27]. In addition, upregulated FOXO1 can significantly increase the basal promoter activity of AdipoR1 [28]. AdipoR1 is the most abundant adipose-specific hormone and can inhibit the inflammatory response, promote mitochondrial biogenesis and suppress apoptosis by activating 5’ adenosine monophosphate-activated protein kinase (AMPK) [29–32]. For example, AdipoR1 can target APN to suppress the release of proinflammatory cytokines such as TNF-α and IL-1β and plays a therapeutic role in Alzheimer’s disease via the AdipoR1-AMPK-NF-κB signaling pathway [33]. Tang et al. reported that AdipoR1 participates in adiponectin-induced IL-6 expression and release by activating the AdipoR1-AMPK-NF-κB signaling pathway in human synovial fibroblasts [34]. Additionally, in human bronchial epithelial cells, AdipoR1 significantly decreases with increasing NP-Nd2O3 concentration and plays a crucial role in the NF-κB pathway-induced inflammatory response [35]. Thus, AdipoR1 may assist in the development of novel efficacious therapies for ischaemic cardiac impairment.

In this study, we identified mmu_circ_42424, also named circDhx32, as a novel conserved circRNA transcribed from the exons of the coding gene DEAH-box helicase 32 (Dhx32), which was markedly upregulated in I/R-induced mouse hearts and H/R-treated cardiomyocytes. However, the aberrant expression of circDhx32 was closely associated with the level of m^6^A modification, and ALKBH5 could remove the m^6^A modification sites of circDhx32, leading to increased RNA stability and nuclear accumulation in a YTHDF2- and YTHDC1-dependent manner. We subsequently aimed to confirm whether circDhx32 could modulate the inflammatory response to cardiac I/R injury. Our results indicated that circDhx32 could bind to FOXO1 to inhibit its transcriptional activation of AdipoR1; in contrast, silencing circDhx32 reversed the downregulated protein expression of AdipoR1 and it induced inflammatory response in cardiac I/R model mice. Thus, circDhx32 may be a pivotal factor that links cardiac I/R injury to inflammation and has advantages as a potential therapeutic target for ischaemic heart disease and prognosis in the clinic.

## Materials and methods

### Mouse model of cardiac I/R injury

This research was approved by the Institutional Animal Care and Use Committee of Harbin Medical University (IRB3056724). All experimental procedures were performed in accordance with the NIH Guide for the Care and Use of Laboratory Animals. Eight-week-old male C57BL/6 mice (Changsheng Bio-technology Co., Ltd., Liaoning, China) weighing 22–24 g were chosen to establish an I/R mouse model. After anaesthesia and trachea cannulation, the left anterior descending coronary artery (LAD) was ligated with 7–0 nylon at 1–2 mm below the left auricle for 45 min, followed by blood reperfusion for 24 h or 2 weeks. In the sham group, the sutures were passed through the LAD without ligation. Echocardiography was used to evaluate cardiac function, and the mice were subsequently anatomized to obtain tissues and serum for further experiments.

### Gene delivery in mice

C57BL/6 male mice were injected with the virus AAV9 (3 × 10^11^ virus particles; shcircDhx32-V, shNC-V) vector loaded with a short hairpin RNA fragment and a cTNT promoter coupled to the GFP gene via the tail vein to silence circDhx32 (Genechem Biotechnology, Shanghai, China). Cardiac I/R surgery was performed 4 weeks after virus injection.

### Echocardiographic analysis

The mice were anaesthetized via intraperitoneal injection of 2% avertin. M-mode echocardiography was used to evaluate left ventricular (LV) function with a Vevo2100 echocardiographic system (Visualsonics, Toronto, Ontario, Canada) at a probe frequency of 10 MHz. A noninvasive ultrasound beam was used to generate a vertical shot of the left ventricle of each mouse to assess the ejection fraction (EF) and fractional shortening (FS), followed by at least three consecutive cardiac cycle measurements.

### Evans blue and TTC staining

Evans blue and TTC staining were used to visualize the infarct size (IS) and the area at risk (AAR) in the I/R model mice. In brief, 2% Evans blue (Solarbio, Beijing, China) was injected into the mice, and the hearts were quickly frozen. Then, the hearts were cut into four slices with a thickness of 1 mm, after which they were stained with 2% TTC dye buffer (Solarbio) in the dark at 37 °C for 20 min. A stereomicroscope (Zeiss, Jena, Germany) was used to photograph the cardiac sections, and ImageJ (NIH, MD, USA) was used to calculate the percentage of the infarct area.

### H&E and Masson’s trichrome staining

The heart sections were dewaxed with xylene, followed by dehydration with alcohol. According to the manufacturer’s instructions, H&E (Solarbio) was used to stain the nucleus and cytoplasm, while Masson’s trichrome staining kit (Solarbio) was performed to evaluate fibrosis levels, followed by image analysis software to observe and photograph.

### Assay kits

As previously described [36], an LDH detection assay kit (Nanjing Jiancheng Bioengineering Institute, Nanjing, China) was used to measure lactate dehydrogenase (LDH), creatine kinase MB (CK-MB) and cardiac troponin-T (cTnT) levels via CK-MB and cTnT ELISA kits (Elabscience, Wuhan, China).

### Primary cardiomyocyte culture

For neonatal mouse ventricular cardiomyocytes, the hearts of 1- to 3-day-old mice were digested in a solution containing trypsin (Solarbio) and PBS for 8–12 h in a 4 °C shaker. After being washed in Dulbecco’s modified Eagle’s medium (DMEM, Biological Industries, Migdal HaEmek, Israel), the tissues were digested with collagenase Type II (Thermo Fisher Scientific, MA, USA) until the hearts disappeared. Then, the lysates were collected and centrifuged at 1500 rpm for 10 min to precipitate the cells. Finally, DMEM supplemented with 10% fetal bovine serum (Gibco, CA, USA), 100 U/mL penicillin and 100 μg/mL streptomycin was used to resuspend the cells, and the cells were seeded onto plates for subsequent experiments.

### Plasmid construction, siRNA interference and transfection

Cardiomyocytes were cultured at 75% density, followed by plasmid transfection (5 μg) via Lipofectamine 3000 reagent (Invitrogen, CA, USA) or siRNAs at a final concentration of 50 nM using X-treme gene siRNA transfection reagent (Roche, Basel, Switzerland) according to the manufacturer’s instructions. CircDhx32 siRNA sequences were chemically synthesized by GenePharma (Shanghai, China). YTHDF2-specific small interfering RNA (si-YTHDF2) and negative control siRNA (si-NC) were purchased from RiboBio (Guangzhou, China). ALKBH5-specific siRNA (si-ALKBH5) and FOXO1-specific siRNA (si-FOXO1) were obtained from Sevenbio (Beijing, China). The sequences of siRNA were shown in Table S1 (Supporting Information). After transfection, the cells were harvested or subjected to hypoxia for 12 h and reoxygenation for 24 h to establish the H/R model.

### Assessment of cell viability via the CCK-8 assay

Cardiomyocytes were cultured in 96-well plates and incubated with CCK-8 solution (Beijing Labgic Technology Co., Ltd., Beijing, China), followed by incubation at 37 °C for 1.5–2 h in the dark. For analysis of cell viability, the absorbance was measured at absorbance values 450 nm using a colorimetric microplate reader.

### Live/dead cell staining

For analysis of cell viability, a live/dead assay kit (#L3224, Life Technologies, CA, USA) was used to incubate cardiomyocytes. The live and dead cells were stained green and red, respectively, according to the manufacturer’s instructions. A laser scanning confocal microscope was used to display the fluorescence intensity of the live and dead cells, as previously described [37, 38].

### Fluorescence in situ hybridization-immunofluorescence (FISH-IF) colocalization

FISH was performed with a FISH Kit (RiboBio). An oligonucleotide-modified probe sequence for circDhx32 was synthesized by RiboBio. Cardiomyocytes were fixed and permeabilized with 4% formaldehyde and 0.5% Triton-100 according to the manufacturer’s instructions. Then, the cells were prehybridized at 37 °C for 30 min and incubated with hybridization solution containing the circDhx32 probe at 37 °C overnight. After being washed in each concentration gradient SSC, the cells were incubated with an anti-FOXO1 antibody and a fluorescent secondary antibody. Finally, DAPI was used to stain the nucleus, and fluorescence staining was examined under a confocal laser scanning microscope (Zeiss).

### Western blotting

Total protein was extracted from cardiomyocytes and the myocardium with RIPA lysis buffer (Beyotime Institute of Biotechnology, Shanghai, China) containing protease inhibitors (Roche) and phosphatase inhibitors (Roche), and a nuclear and cytoplasmic extraction kit (78835, Thermo Scientific Pierce, WA, USA) was used to separate the nuclear and cytoplasmic proteins. A bicinchoninic acid protein kit (Beyotime Institute of Biotechnology) was used to determine the protein concentration. Protein lysates were separated by sodium dodecyl sulfate‒polyacrylamide gel electrophoresis (SDS‒PAGE), followed by transfer to nitrocellulose membranes (Millipore, MA, USA). After blocking, the membranes were incubated with primary antibodies against GAPDH (#TA-08, ZsBio, Beijing, China, 1:1000), lamin B1 (#A11495, ABclonal, Wuhan, China, 1:500), YTHDF2 (24744-1-AP, Proteintech, Wuhan, China, 1:1000), YTHDC1 (14392-1-AP, Proteintech, 1:1000), ALKBH5 (67811-1-AP, Proteintech, 1:1000), FOXO1 (18592-1-AP, Proteintech, 1:1000), the adiponectin receptor (14361-1-AP, Proteintech, 1:1000), p65 (10745-1-AP, Proteintech, 1:1000), p-p65 (#ab76302, Abcam, Cambridge, UK, 1:1000), AMPKα (D5A2) (5831S, Cell Signaling Technology, MA, USA, 1:1000) and p-AMPKα (Thr172) (2535S, Cell Signaling Technology, 1:1000) at 4 °C overnight. The membranes were subsequently incubated with secondary antibodies in the dark for 50 min at room temperature, and the protein bands were quantified by an Odyssey infrared imaging system (LI-COR, NE, USA). GAPDH and lamin B1 were used as internal controls.

### RNA isolation and qRT‒PCR

Total RNA was extracted from cardiomyocytes and the myocardium using TRIzol reagent (Invitrogen). A Cytoplasmic and Nuclear RNA Purification Kit (Norgen Biotek; Ontario, Canada) was used to isolate cytoplasmic and nuclear RNA from cells according to the manufacturer’s instructions. A NanoDrop ND-8000 (Thermo Fisher Scientific) was used to measure the RNA concentration, and a reverse transcription kit (Toyobo, Osaka, Japan) was used to obtain cDNA. Then, real-time PCR was performed via SYBR Green Master Mix (Toyobo) to quantify the circDhx32, Dhx32, FOXO1, AdipoR1, ALKBH5, YTHDF2, IL-6, TNF-α and IL-1β levels on an ABI 7900HT Fast Real-Time PCR System (Applied Biosystems, CA, USA). GAPDH or 18S was used to normalize RNA expression as an internal control. The following primers used in this study were shown in Table S2 (Supporting Information).

### Actinomycin D (Act D) and RNase R treatment

Act D and RNase R were used to verify the characteristics of the circRNAs. Act D (5 μg/mL, MedchemExpress, NJ, USA) was used to block the transcription of DNA to RNA and assess the stability of circRNA. Total RNA (1 μg) was incubated with 1 U of RNase R (Epicenter, WI, USA) at 37 °C for 10 min to digest linear RNA. The circDhx32 and GAPDH expression levels were subsequently determined by qRT‒PCR.

### m^6^A-circRNA epitranscriptomic microarray and bioinformatic analysis

Sample preparation and microarray hybridization were performed according to Arraystar’s standard protocols. Briefly, 2 μg of total RNA was incubated with an anti-m^6^A rabbit polyclonal antibody (Synaptic Systems, Goettingen, Germany) for immunoprecipitation. The m^6^A-modified RNAs were eluted from the magnetic beads and marked as “IP”, and the unmodified RNAs were recovered from the supernatant as “Sup”. The “IP” and “Sup” RNAs were treated with RNase R to eliminate linear RNAs, and then, a RNeasy MinElute Cleanup Kit (cat. #74204, Qiagen, Dusseldorf, Germany) was used to purify the remaining RNA. Next, the enriched “IP” and “Sup” RNAs were labeled with Cy3 (for “Sup”) or Cy5 (for “IP”) and purified using a Super RNA Labeling Kit (Arraystar, MD, USA) and a RNeasy Mini Kit, respectively. The labeled RNAs were subsequently hybridized onto an Arraystar circRNA Epitranscriptomic Microarray (8 × 15 K, Arraystar). After being scanned in two color channels by an Agilent Scanner G2505C (Agilent, Beijing, China), the microarrays were analysed using Agilent Feature Extraction software (version 11.0.1.1). Differentially expressed m^6^A-methylated circRNAs with a fold change ( | FC | ≥ 1.5) and statistically significant (*P* ≤ 0.05) thresholds were screened between the sham and I/R groups.

### RNA-binding protein immunoprecipitation (RIP) assay

A Magna RIP RNA-Binding Protein Immunoprecipitation Kit (Millipore) was used to verify the interactions between proteins and RNAs. The RNA‒protein complexes were collected via RIP lysis buffer and incubated overnight with 50 μL of protein-A/G agarose beads (Roche) and antibodies against m^6^A (#202003, Synaptic Systems, 5 μg) or YTHDF2 (#24744-1-AP, Proteintech, 5 μg), YTHDC1 (14392-1-AP, Proteintech, 5 μg) or FOXO1 (18592-1-AP, Proteintech, 5 μg) at 4 °C with rotation. After the immunoprecipitated RNA was purified, the final products were subjected to qRT‒PCR analysis and agarose gel electrophoresis to quantify the expression of RNAs.

### Luciferase reporter assay

After the cells were transfected with the FOXO1 and circDhx32 plasmids, a Dual-Luciferase Reporter Assay System (Promega, WI, USA) was used to collect and lyse the cells according to the manufacturer’s protocol. For analysis of the relative luciferase activity, the ratio of firefly and Renilla luciferase activities was measured and normalized to Renilla luciferase activity.

### Chromatin immunoprecipitation (ChIP) assay

For ChIP assays, a SimpleChIP^®^ Plus Sonication Chromatin IP Kit (Cell Signaling Technology) was used to detect the binding capacity between the AdipoR1 promoter and FOXO1 with or without transfection of the circDhx32 plasmid. In brief, cardiomyocytes were treated with 37% formaldehyde to crosslink proteins to DNA. After the nucleus was lysed completely and the crosslinked chromatin preparation was digested, the obtained chromatin was incubated with 10 μg of anti-FOXO1 (Proteintech) antibody or 1 μg of isotype-matched control IgG (Cell Signaling Technology) overnight. Then, protein G magnetic beads were used to collect the immunoprecipitated complex, and a spin column was used to elute and purify the complexes. The purified DNA was subsequently separated and measured via qPCR using specific primers. The primers used were AdipoR1-F 5’-AGGATGGCCTCGAGTACAAG-3’ and AdipoR1-R 5’-AATTCGCCCTGTAGATTCGC-3’.

### Electrophoretic mobility shift assay (EMSA)

Nuclear and cytoplasmic proteins were extracted using the NE-PER Nuclear and Cytoplasmic Extraction Reagents Kit (Thermo Fisher Scientific) according to the manufacturer’s protocol. A LightShift Chemiluminescent EMSA Kit (Thermo Fisher Scientific) was subsequently used to perform further experiments. In brief, the binding buffer and labeled probe poly (dI. dC) were used to incubate the nucleus extract at 20 °C for 30 min, after which excessive unlabeled cold competition probes or mutant probes were added for the binding competition experiment. After the conjunctive complex was separated and crosslinked on a polyacrylamide gel, chemiluminescence was used to visualize the interaction between FOXO1 and the AdipoR1 promoter. The following double-stranded biotin-labeled probe sequences were used: AdipoR1-bF 5’-GTTGGCGTTGTTTAATCCTGCCA-biotin-3’ and AdipoR1-bR 5’-TGGCAGGATTAAACAACGCCAAC-biotin-3’. The cold probe sequences used were AdipoR1-bF 5’-GTTGGCGTTGTTTAATCCTGCCA-3’ and AdipoR1-bR 5’-TGGCAGGATTAAACAACGCCAAC-3’. The mutant probe sequences used were AdipoR1-bF 5’-GTTGGCTGGTGGGCCGACTGCCA-3’ and AdipoR1-bR 5’-TGGCAGTCGGCCCACCAGCCAAC-3’.

### Statistical analysis

All the data are presented as the means ± SEMs of at least three independent experiments, and GraphPad Prism 9.0 (GraphPad Software, CA, USA) was used to analyse the data statistically. Student’s unpaired two-tailed *t* test was used for two-group comparisons, and one-way ANOVA with Dunnett’s correction was applied for multiple group comparisons. A *P* value < 0.05 was considered to indicate a significant difference.

## Results

### Upregulation of circDhx32 in mouse I/R-affected hearts and H/R-induced cardiomyocyte injury

**Fig. 1:**
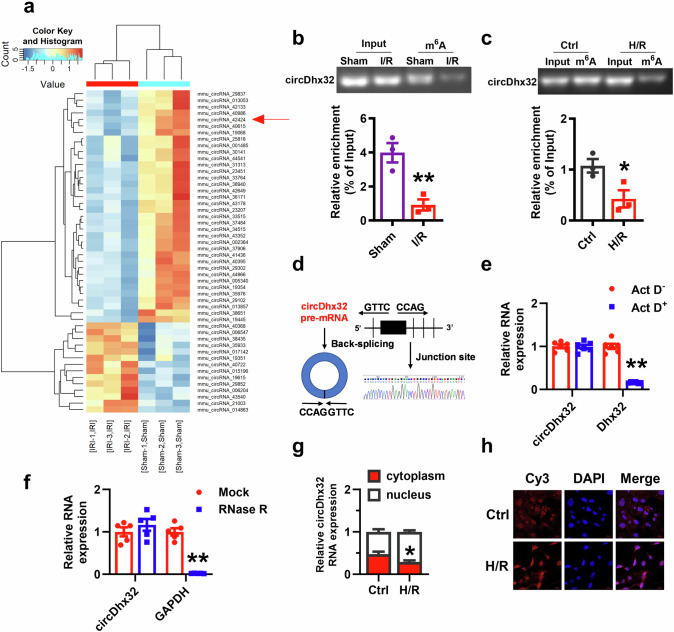
CircDhx32 is upregulated in I/R-treated mouse hearts and H/R-treated cardiomyocytes. **a** Heatmap showing numerous circRNAs with significant differential m^6^A modifications in I/R-treated mice. **b, c** RIP revealed the binding of circDhx32 with a m^6^A antibody in I/R-treated mice or H/R-induced cardiomyocytes. The Y-axis represents the percentage of input for each IP sample according to the formula: %Input =1/10*2^Ct [IP] – Ct [input]^. *n* = 3. ^**^P < 0.01 *vs*. Sham, ^*^P < 0.05 *vs.*^.^ Ctrl. *P* values were determined via an unpaired *t* test. **d** Sanger sequencing was performed to validate that circDhx32 originated from its host gene. **e** Expression of linear RNA (Dhx32) and circDhx32 after Act D treatment for 24 h. *n* = 6. ^**^P < 0.01 *vs*. Act D^-^. *P* values were determined via an unpaired *t* test. **f** Relative RNA expression of GAPDH and circDhx32 in cardiomyocytes with or without RNase R incubation. *n* = 5. ^**^P < 0.01 *vs*. Mock. *P* values were determined via an unpaired *t* test. **g** An RNA fractionation assay was used to evaluate the distribution of circDhx32 in the nucleus and cytoplasm under H/R conditions. *n* = 5. *P < 0.05 *vs*. Ctrl. *P* values were determined via an unpaired *t* test. **h** Subcellular localization of circDhx32 in H/R-induced cardiomyocytes was detected via FISH. The nuclei were stained with DAPI. Cy3 was used to stain for circDhx32. Scale bar = 20 µm. *n* = 4.

Previous studies have indicated that m^6^A methylation is involved in various RNAs, such as mRNAs and long noncoding RNAs [39, 40]. To screen circRNAs associated with m^6^A modification, we performed m^6^A-circRNA epitranscriptomic microarray analysis on mouse hearts obtained from three normal mice and three mice with cardiac ischaemia‒reperfusion injury. The results revealed that 210 circRNAs, 15 upregulated and 195 downregulated m^6^A-modified circRNAs, were significantly altered compared with those in the sham group (Fig. 1a, Supplementary Fig. S1a, b). Here, we first examined the changes in the expression of the circRNAs with the most significant differences in m^6^A modifications in cardiac I/R model mice and H/R-induced cardiomyocytes and immunofluorescence confirmed the fineness of primary cardiomyocytes (Supplementary Fig. S1c–e). We found that the expression of mmu_circ_42424 showed the greatest increase. We named it circDhx32 owing to its host gene Dhx32 and chose this circRNA for further study. MeRIP assays further confirmed that the m^6^A modification of circDhx32 was obviously reduced after I/R injury and H/R treatment (Fig. 1b, c). To verify the circular structure of circDhx32, we designed divergent primers to perform Sanger sequencing, which revealed that circDhx32 was generated from exon 22 to exon 30 of the linear Dhx32 gene through backsplicing (Fig. 1d). Furthermore, compared with the linear gene, circDhx32 was more stable after treatment with Act D and RNase R exonucleases (Fig. 1e, f). To explore the subcellular localization of circDhx32 in cardiomyocytes, we conducted cytoplasmic and nuclear RNA analysis and FISH experiments. The results revealed that circDhx32 was located in both the nucleus and the cytoplasm, whereas H/R strongly increased circDhx32 in the nucleus (Fig. 1g, h). These results indicated that circDhx32 was significantly upregulated in I/R model mouse hearts and H/R-treated cardiomyocytes.

### Silencing circDhx32 alleviates I/R-induced heart injury in mice

**Fig. 2:**
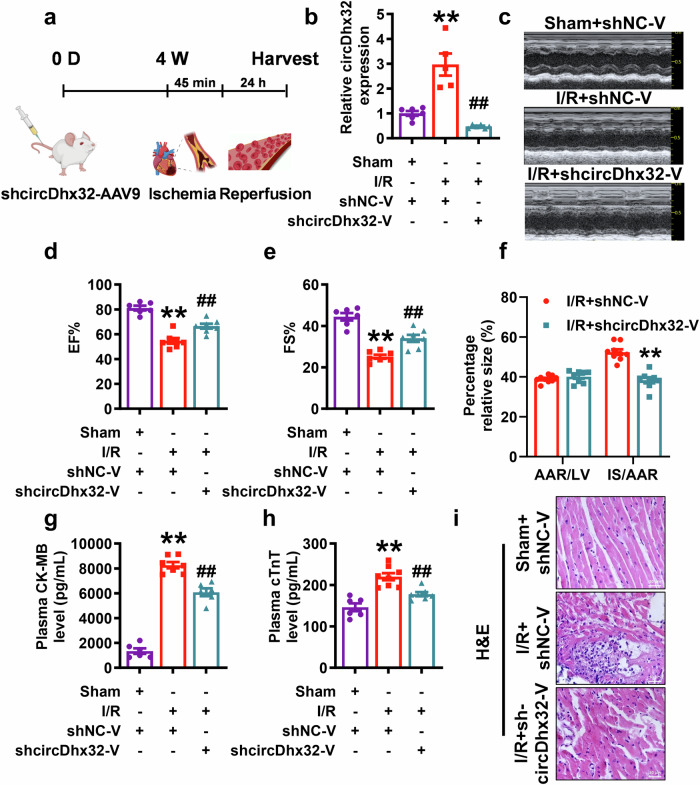
Cardiomyocyte-specific circDhx32 knockdown relieves myocardial impairment in I/R model mice. **a** Schematic diagram showing that mice were injected with the AAV9 vector carrying an NC-shRNA or circDhx32-shRNA fragment via the tail vein, after which the I/R model was established and harvested at the corresponding time points. **b** qRT‒PCR was performed to test circDhx32 expression. *n* = 5‒6. ^**^P < 0.01 *vs*. Sham+shNC-V; ^##^*P* < 0.01 *vs*. I/R+shNC-V. One-way ANOVA followed by Dunnett’s multiple comparisons test was performed to evaluate significant differences. **c**‒**e** Representative images of echocardiographs and statistics of EF and FS. *n* = 6**‒**7. ^**^P < 0.01 *vs*. Sham**+**shNC-V; ^##^*P* < 0.01 *vs*. I/R+shNC-V. One-way ANOVA followed by Dunnett’s multiple comparisons test was performed to evaluate significant differences. **f** Evans blue/TTC staining was used to evaluate infarct size in I/R-treated mouse hearts. *n* = 8‒9. ^**^P < 0.01 *vs*. I/R+shNC-V. One-way ANOVA followed by Dunnett’s multiple comparisons test was performed to evaluate significant differences. **g** Detection of plasma CK-MB levels in the mice. *n* = 6‒7. ^**^P < 0.01 *vs*. Sham+shNC-V; ^##^*P* < 0.01 *vs*. I/R+shNC**-**V. One-way ANOVA followed by Dunnett’s multiple comparisons test was performed to evaluate significant differences. **h** Concentration of cTnT in mouse serum. *n* = 6‒8. ^**^P < 0.01 *vs*. Sham+shNC-V; ^##^*P* < 0.01 *vs*. I/R+shNC-V. One-way ANOVA followed by Dunnett’s multiple comparisons test was performed to evaluate significant differences. **i** Representative images of H&E staining of the myocardium. Scale bar = 20 μm. *n* = 6**‒**8.

To explore the functional role of circDhx32 during cardiac I/R injury, we performed loss-of-function experiments using an AAV9 vector that carried a sequence fragment (sh-circDhx32) to silence circDhx32 (Fig. 2a). GFP staining and qRT‒PCR confirmed the successful delivery of the AAV9 vector, and silencing circDhx32 reversed the increase in circDh32 levels observed post-I/R injury but had no effect on the expression level of Dhx32 in the myocardium (Fig. 2b, Supplementary Fig. S2a, b). Echocardiography revealed that deletion of circDhx32 rescued the decreases in EF and FS in cardiac I/R model mice but did not affect cardiac function in normal mice (Fig. 2c–e, Supplementary Fig. S2c, d). Similarly, circDhx32 silencing decreased the infarct size and the release of CK-MB and cTnT after cardiac I/R injury (Fig. 2f–h, Supplementary Fig. S2e). H&E staining revealed that downregulation of circDhx32 reversed I/R-induced disorders in cardiomyocyte arrangement and increased inflammatory cell infiltration (Fig. 2i). Then we investigated whether silencing circDhx32 played a role in I/R-induced cardiac remodeling in two weeks post-I/R injury. The echocardiography results showed that silencing circDhx32 dramatically improved cardiac function following I/R injury (Supplementary Fig. S3a–c). Moreover, we performed Evans-blue/TTC staining and found that deletion of circDhx32 decreased the infarct size after cardiac I/R injury (Supplementary Fig. S3d, e). H&E and masson staining revealed that downregulation of circDhx32 reversed I/R-induced disorders in cardiomyocyte arrangement and collagen deposition increase (Supplementary Fig. S3f–h). The above data revealed that silencing circDhx32 normalized cardiac function and alleviated myocardial impairment and remodeling in the context of I/R injury.

### Decreased circDhx32 relieves cardiomyocyte injury after H/R

**Fig. 3:**
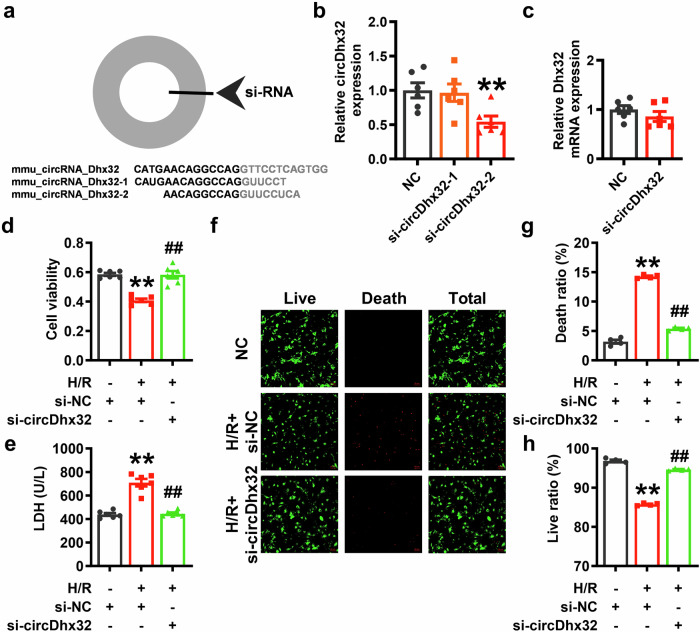
CircDhx32 downregulation alleviates cardiomyocyte injury upon H/R treatment. **a** Schematic diagram of circDhx32 siRNA. **b** Relative circDhx32 expression after transfection of circDhx32 siRNA (si-circDhx32) or NC siRNA (si-NC). *n* = 6. ^**^P < 0.01 *vs*. NC. *P* values were determined via an unpaired *t* test. **c** mRNA expression level of Dhx32 in cardiomyocytes. *n* = 6. *P* values were determined via an unpaired *t* test. **d** Cell viability was examined by CCK-8 assays of cardiomyocytes after H/R. *n* = 6. ^**^P < 0.01 *vs*. si-NC; ^##^*P* < 0.01 *vs*. H/R+si-NC. One-way ANOVA followed by Dunnett’s multiple comparisons test was performed to evaluate significant differences. **e** Detection of LDH release in cardiomyocytes after H/R. *n* = 6. ^**^P < 0.01 *vs*. si-NC; ^##^*P* < 0.01 *vs*. H/R+si-NC. One-way ANOVA followed by Dunnett’s multiple comparisons test was performed to evaluate significant differences. **f**‒**h** Representative images of live and dead cell staining and data analysis of the death ratio and live ratio (%). Scale bar = 50 μm. *n* = 4. ^**^P < 0.01 *vs*. si-NC; ^##^*P* < 0.01 *vs*. H/R+si-NC. One-way ANOVA followed by Dunnett’s multiple comparisons test was performed to evaluate significant differences.

We further investigated the effect of circDhx32 silencing on H/R-induced cardiomyocyte injury. We designed a small interfering RNA to silence circDhx32 and found that the knockdown effect of si-circDhx32-2 was more significant (Fig. 3a, b). Consistent with our expectations, the silencing of circDhx32 had no effect on Dhx32 expression (Fig. 3c). In addition, si-circDhx32 alleviated the detrimental effects induced by H/R damage, including decreased cell viability accompanied by increased LDH release (Fig. 3d–h). Collectively, the above results showed that the inhibition of circDhx32 protected against H/R-induced cardiomyocyte injury.

### Reduced m^6^A modification of circDhx32 increases its stability and nuclear retention

**Fig. 4:**
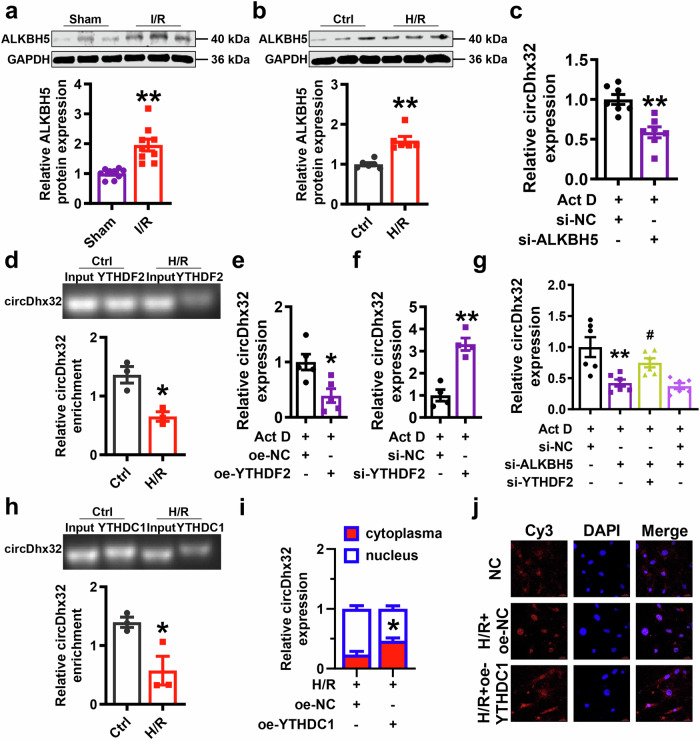
The RNA stability and location of circDhx32 are regulated by its m^6^A modification level. **a** The protein expression level of ALKBH5 in I/R-treated mice. *n* = 9. **P < 0.01 *vs*. Sham. *P* values were determined via an unpaired *t* test. **b** Western blot analysis of ALKBH5 in H/R-induced NMVCs. *n* = 6. ^**^P < 0.01 *vs*. Ctrl. *P* values were determined via an unpaired *t* test. **c** The level of circDhx32 in cardiomyocytes transfected with si-NC or si-ALKBH5 and then subjected to Act D treatment at the indicated time points. *n* = 7. ^**^P < 0.01 *vs*. Act D+si-NC. *P* values were determined via an unpaired *t* test. **d** RIP was performed to assess relative circDhx32 enrichment with a YTHDF2 antibody. *n* = 3. ^*^P < 0.05 *vs*. Ctrl. *P* values were determined via an unpaired *t* test. **e** The expression of circDhx32 treated with Act D upon YTHDF2 overexpression. *n* = 5. ^*^P < 0.05 *vs*. Act D+oe-NC. *P* values were determined via an unpaired *t* test. **f** The expression of circDhx32 in cells treated with Act D upon YTHDF2 silencing. *n* = 4. ^**^P < 0.01 *vs*. Act D+si-NC. *P* values were determined via an unpaired *t* test. **g** After cotransfection with si-YTHDF2 and si-ALKBH5 for 48 h, cardiomyocytes were treated with Act D, and circDhx32 expression was quantified via qRT‒PCR at the indicated time points. *n* = 6. ^**^P < 0.01 *vs*. Act D+si-NC; ^#^*P* < 0.05 *vs*. Act D+si-ALKBH5+si-NC. One-way ANOVA followed by Dunnett’s multiple comparisons test was performed to evaluate significant differences. **h** The binding of circDhx32 to YTHDC1 was verified by a RIP assay. *n* = 3. ^*^P < 0.05 *vs*. Ctrl. *P* values were determined via an unpaired *t* test. **i** Cytoplasmic and nuclear fractionation assays revealed the localization of circDhx32 in H/R-induced cardiomyocytes upon YTHDC1 upregulation. *n* = 5. ^*^P < 0.05 *vs*. H/R+oe-NC. *P* values were determined via an unpaired *t* test. **j** FISH revealed that circDhx32 was located mainly in the nucleus and that overexpression of YTHDC1 reversed this phenomenon. Cy3 and DAPI were used to stain circDhx32 and the nucleus, respectively. Scale bar = 20 µm. *n* = 4‒7. The data are expressed as the means ± SEMs.

Next, we investigated whether the upregulation and nuclear translocation of circDhx32 under pathological conditions were related to its m^6^A modification. Hence, we investigated whether the dysregulation of circRNAs was related to m^6^A modification and found that the m^6^A demethylase ALKBH5 was markedly elevated in cardiac I/R model mice and H/R-treated cardiomyocytes (Fig. 4a, b). To determine whether ALKBH5 influenced circDhx32 stability by regulating its m^6^A modification, we first verified the knockdown efficacy of ALKBH5 (Supplementary Fig. S4a, b) and then used Act D, an inhibitor of DNA transcription. The qRT‒PCR data revealed that the stability of circDhx32 was significantly decreased by silencing ALKBH5 in Act D-treated cardiomyocytes (Fig. 4c).

Our previous study confirmed that the m^6^A reader YTHDF2 can recognize and bind to the m^6^A modification site of RNA to promote its degradation [41]. The RIP results revealed the binding of circDhx32 to YTHDF2, while H/R decreased the binding capacity (Fig. 4d). Similarly, we found that upregulated YTHDF2 facilitated circDhx32 decay; in contrast, decreased YTHDF2 increased its stability (Fig. 4e, f; Supplementary Fig. S4c–f). We then cotransfected YTHDF2 and ALKBH5 siRNAs into the cells and ascertained that silencing YTHDF2 reversed the degradation of circDhx32 caused by si-ALKBH5 after Act D treatment (Fig. 4g). YTHDC1 can regulate RNA nuclear export through the identification of m^6^A modification sites [42]. Interestingly, YTHDC1 could also bind to circDhx32 (Fig. 4h). Cytoplasmic and nuclear RNA analyses and FISH experiments revealed that upregulation of YTHDC1 reversed H/R-induced accumulation of circDhx32 in the nucleus (Fig. 4i, j; Supplementary Fig. S4g, h). Taken together, our data suggested that ALKBH5-mediated m^6^A methylation of circDhx32 regulates its stability and nuclear location in a YTHDF2- and YTHDC1-dependent manner.

### CircDhx32 interacts with FOXO1 and inhibits its transcriptional activation of AdipoR1

**Fig. 5:**
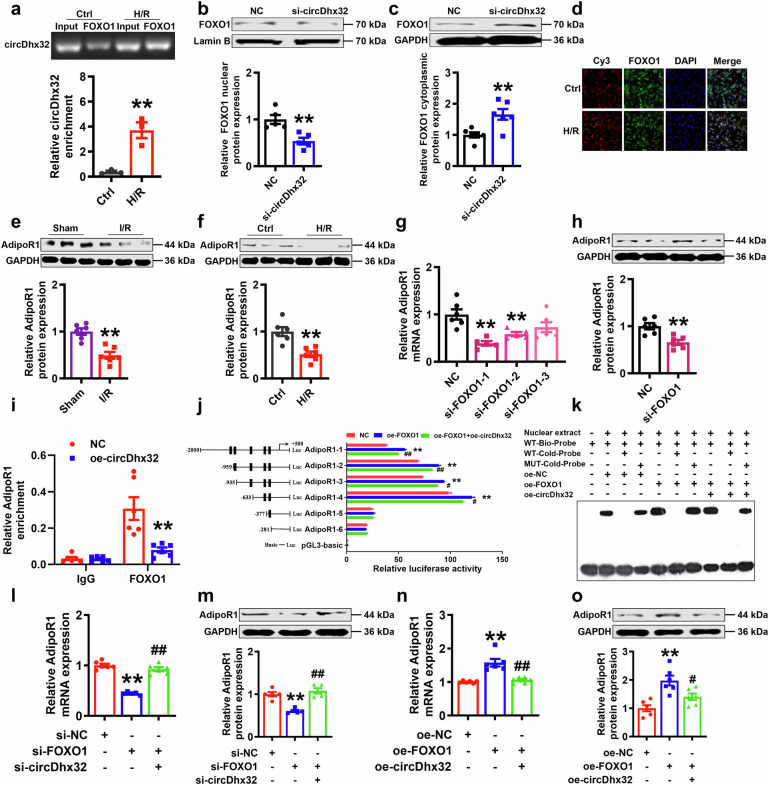
CircDhx32 suppresses the transcription of AdipoR1 by competitively binding to FOXO1. **a** RIP assay showing the increased interaction capacity between circDhx32 and FOXO1 under H/R conditions. *n* = 3. ^**^P < 0.01 *vs*. Ctrl. *P* values were determined via an unpaired *t* test. **b, c** Western blot analysis of the nuclear and cytoplasmic protein expression of FOXO1 upon circDhx32 silencing. *n* = 5‒6. ^**^P < 0.01 *vs*. NC. *P* values were determined via an unpaired *t* test. **d** FISH-IF assay showing the colocalization of circDhx32 (red) and FOXO1 (green) after H/R. DAPI (blue) was used to stain the nuclei. Scale bar = 20 μm. *n* = 4. **e, f** Western blot analysis of AdipoR1 in I/R-treated mouse hearts and H/R-induced cardiomyocytes. *n* = 6. ^**^P < 0.01 *vs*. Sham, ^**^P < 0.01 *vs*. Ctrl. *P* values were determined via an unpaired *t* test. **g** Relative mRNA level of AdipoR1 after transfection with si-NC or si-FOXO1. *n* = 5‒6. ^**^P < 0.01 *vs*. NC. *P* values were determined via an unpaired *t* test. **h** The protein expression of AdipoR1 after FOXO1 silencing. *n* = 6. ^**^P < 0.01 *vs*. NC. *P* values were determined via an unpaired *t* test. **i** ChIP‒qPCR was performed to analyse the binding of FOXO1 to the AdipoR1 promoter upon circDhx32 overexpression. IgG was used as a negative control. *n* = 6. ^**^P < 0.01 *vs*. NC. *P* values were determined via an unpaired *t* test^.^ **j** Relative luciferase activity was determined in NMVCs cotransfected with a luciferase reporter plasmid containing the AdipoR1 promoter sequence and an overexpression plasmid containing circDhx32 and FOXO1. *n* = 3. ^**^P < 0.01 *vs*. NC; ^#^*P* < 0.05, ^##^*P* < 0.01 *vs*. oe-FOXO1. One-way ANOVA followed by Dunnett’s multiple comparisons test was performed to evaluate significant differences. **k** RNA EMSAs were used to detect the interaction between AdipoR1 and FOXO1 in primary cardiomyocytes with or without circDhx32 plasmid transfection. **l** Relative AdipoR1 mRNA expression was examined by qRT‒PCR in cardiomyocytes cotransfected with si-FOXO1 and si-circDhx32. *n* = 5‒6. ^**^P < 0.01 *vs*. si-NC; ^##^*P* < 0.01 *vs*. si-FOXO1. One-way ANOVA followed by Dunnett’s multiple comparisons test was performed to evaluate significant differences. **m** The protein level of AdipoR1 was determined by Western blotting. *n* = 5. ^**^P < 0.01 *vs*. si-NC; ^##^*P* < 0.01 *vs*. si-FOXO1. One-way ANOVA followed by Dunnett’s multiple comparisons test was performed to evaluate significant differences. **n, o** mRNA and protein levels of AdipoR1 in cardiomyocytes after overexpression of FOXO1 and circDhx32. *n* = 6. ^**^P < 0.01 *vs*. oe-NC; ^#^*P* < 0.05, ^##^*P* < 0.01 *vs*. oe-FOXO1. One-way ANOVA followed by Dunnett’s multiple comparisons test was performed to evaluate significant differences.

Next, we investigated the underlying mechanism of circDhx32 regulation in cardiac I/R model mice and H/R-induced cardiomyocyte injury. m^6^A-circRNA epitranscriptomic microarray analysis revealed that these m^6^A-dysregulated circRNAs strongly bound to proteins to exert their functions after cardiac I/R injury (Supplementary Fig. S5a, b). Therefore, we used RPISeq (http://pridb.gdcb.iastate.edu/RPISeq/) to predict interacting proteins of circDhx32. We found that circDhx32 had stronger interaction probabilities with FOXO1 and that the expression of FOXO1 increased under pathological conditions both in vivo and in vitro (Supplementary Fig. S6a–c). RIP assays demonstrated that FOXO1 could interact with circDhx32 and be more strongly recognized by circDhx32 in H/R-induced cardiomyocyte injury (Fig. 5a). Consequently, we explored whether the nucleoplasmic distribution of FOXO1 was regulated by circDhx32. The Western blot results revealed that silencing circDhx32 was responsible for decreasing FOXO1 nuclear distribution and increasing its cytoplasmic accumulation (Fig. 5b, c). Similarly, H/R stimulation led to increased circDhx32 RNA and FOXO1 protein expression, which also prominently increased their colocalization and interaction, especially in the nuclear fraction (Fig. 5d). Next, we screened the downstream promoter that binds to FOXO1 via JASPAR (JASPAR-A database of transcription factor binding profiles, https://jaspar.elixir.no/), and 10 sequences of FOXO1 recognition sites were found in the AdiopoR1 promoter region (Supplementary Fig. S6d, e). In addition, the protein levels of AdipoR1 in cardiac I/R model mice and H/R-treated cardiomyocytes were sharply downregulated (Fig. 5e, f). qRT‒PCR and Western blot assays confirmed that si-FOXO1 reduced the mRNA and protein levels of AdipoR1 (Fig. 5g, h; Supplementary Fig. S6f, g). Notably, a ChIP assay confirmed that FOXO1 could bind directly to the promoter region of AdipoR1, whereas overexpression of circDhx32 repressed the transcriptional activation of FOXO1 to AdipoR1 (Fig. 5i, Supplementary Fig. S6h). A dual-luciferase reporter gene assay revealed that the regulation of the AdipoR1 level was mediated by potential FOXO1 binding sites in AdipoR1 located between −613 and −623 bp, whereas this transcriptional activation was blocked upon circDhx32 overexpression (Fig. 5j). An EMSA further confirmed this result (Fig. 5k). Silencing circDhx32 rescued the reduction in AdipoR1 expression induced by si-FOXO1, whereas circDhx32 overexpression inhibited this transcriptional activation (Fig. 5l–o, Supplementary Fig. S6i). Taken together, the above results revealed that circDhx32 repressed AdipoR1 transcription by competing with AdipoR1 for FOXO1.

### CircDhx32 regulates the inflammatory response via the AdipoR1-AMPK-NF-κB signaling pathway

**Fig. 6:**
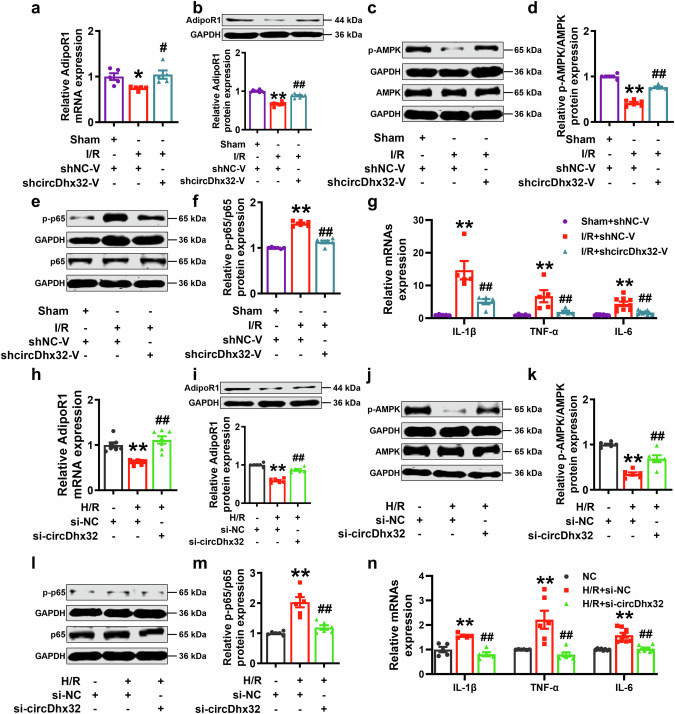
CircDhx32 regulates the inflammatory response via the AdipoR1-AMPK-NF-κB signaling pathway in cardiac I/R model mice and H/R-induced cardiomyocytes. **a, b** The mRNA and protein levels of AdipoR1 in I/R model mice were measured by qRT‒PCR and Western blotting. *n* = 5‒8. ^*^P < 0.05, ^**^P < 0.01 *vs*. Sham+shNC-V; ^#^*P* < 0.05, ^##^*P* < 0.01 *vs*. I/R+shNC-V. One-way ANOVA followed by Dunnett’s multiple comparisons test was performed to evaluate significant differences. **c**‒**f** Western blot analysis was performed to test p-AMPK/AMPK and p-p65/p65 protein expression in I/R-treated mouse hearts. *n* = 6. ^**^P < 0.01 *vs*. Sham+shNC-V; ^##^*P* < 0.01 *vs*. I/R+shNC-V. One-way ANOVA followed by Dunnett’s multiple comparisons test was performed to evaluate significant differences. **g** IL-1β, TNF-α and IL-6 mRNA expression levels in I/R-treated mice. *n* = 5‒9. **P < 0.01 *vs*. Sham+shNC-V; ^##^*P* < 0.01 *vs*. I/R+shNC-V. One-way ANOVA followed by Dunnett’s multiple comparisons test was performed to evaluate significant differences. **h, i** AdipoR1 mRNA and protein expression in H/R-treated cardiomyocytes after transfection with si-circDhx32. *n* = 6‒7. **P < 0.01 *vs*. si-NC; ^##^*P* < 0.01 *vs*. H/R+si-NC. One-way ANOVA followed by Dunnett’s multiple comparisons test was performed to evaluate significant differences. **j**‒**m** Western blot analysis was used to determine p-AMPK/AMPK and p-p65/p65 protein expression in cardiomyocytes after H/R injury upon circDhx32 silencing. *n* = 6. ^**^P < 0.01 *vs*. si-NC; ^##^*P* < 0.01 *vs*. H/R+si-NC. One-way ANOVA followed by Dunnett’s multiple comparisons test was performed to evaluate significant differences. **n** The mRNA levels of IL-1β, TNF-α and IL-6 in cardiomyocytes were measured by qRT‒PCR. *n* = 5‒8. ^**^P < 0.01 *vs*. NC; ^##^*P* < 0.01 *vs*. H/R+si-NC. One-way ANOVA followed by Dunnett’s multiple comparisons test was performed to evaluate significant differences.

Accumulating evidence has shown that AdipoR1 activates the AMPK pathway to play a crucial role in various biological processes [32, 43]. AMPK is involved in modulating NF-κB signaling pathway activation, thus exerting anti-inflammatory effects [44]. Accordingly, we evaluated whether AdipoR1 was involved in the effect of circDhx32-regulated cardiac injury in I/R model mice and found that silencing circDhx32 reversed the I/R-induced decrease in AdipoR1 expression (Fig. 6a, b). The Western blot results revealed that activated AMPK and NF-κB were substantially elevated under I/R conditions and that circDhx32 knockdown reversed this phenomenon (Fig. 6c–f). Furthermore, we investigated whether circDhx32 affects the release of inflammatory factors in I/R-affected heart tissue. As shown in Fig. 6g, injecting shcircDhx32-V decreased the mRNA expression levels of IL-1β, TNF-α and IL-6 post-I/R injury. Next, we investigated whether silencing circDhx32 could also relieve the inflammatory response of cardiomyocytes after H/R. Consistent with the in vivo results, the effects of H/R on AdipoR1 expression, the p-AMPK-to-AMPK (p-AMPK/AMPK) ratio and the p-p65-to-p65 (p-p65/p65) ratio were significantly altered upon circDhx32 silencing (Fig. 6h‒m). In addition, transfection of circDhx32 siRNA decreased proinflammatory cytokine levels in H/R-treated cardiomyocytes (Fig. 6n). In summary, these results suggest that circDhx32 deficiency attenuates cardiac I/R injury by mitigating the inflammatory response via the AdipoR1-AMPK-NF-κB signaling pathway.

## Discussion

**Fig. 7:**
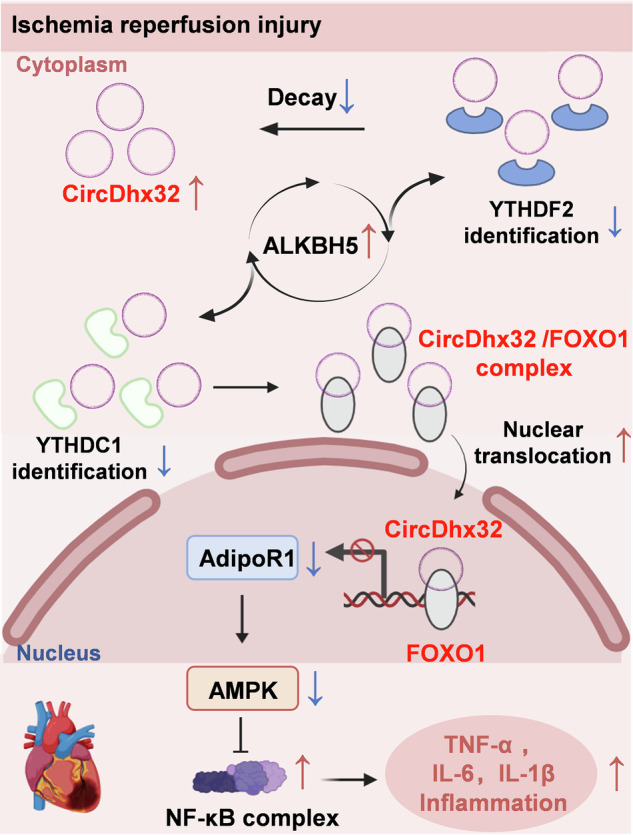
Diagram of the regulatory effect of circDhx32 on cardiac I/R injury. YTHDF2 and YTHDC1 recognize fewer m^6^A modification sites on circDhx32 caused by ALKBH5 and increase its stability and nuclear retention in cardiac I/R model mice. Nuclear circDhx32 binds to FOXO1 and then competitively relieves its ability to activate AdipoR1, ultimately inhibiting the expression of AdipoR1 and thereby exacerbating the inflammatory response in cardiac I/R model mice by regulating the AMPK/NF-κB signaling pathway.

In this study, we aimed to elucidate the association between circDhx32 and cardiac I/R injury. We first found that circDhx32 was increased in I/R-affected hearts and H/R-induced cardiomyocytes, whereas silencing circDhx32 normalized cardiac function, relieved cardiomyocyte impairment and improved myocardial remodeling under pathological conditions. Additionally, the upregulation and nuclear retention of circDhx32 are mediated by the m^6^A demethylase ALKBH5 in a YTHDF2- and YTHDC1-dependent manner. Increased circDhx32 interacted with the transcription factor FOXO1 and carried it into the nucleus, thereby repressing its transcriptional activation of AdipoR1 (Fig. 7).

We first revealed that in cardiac I/R injury, (1) circDhx32 is upregulated after cardiac I/R injury and H/R. (2) the m^6^A demethylase ALKBH5 removes the m^6^A modification sites of circDhx32, thus promoting its stability and nuclear location in a YTHDF2- and YTHDC1-dependent manner. (3) CircDhx32 interacts with FOXO1 and inhibits its transcriptional activation of AdipoR1, accelerating the inflammatory response and cardiac I/R impairment by regulating the AdipoR1-AMPK-NF-κB signaling pathway.

CircRNAs have various advantages, such as high stability and evolutionary conservation, and play crucial roles in diverse biological processes [45]. Recently, increasing evidence has shown that circRNAs participate in multiple cardiovascular diseases, including myocardial infarction [45], cardiac hypertrophy [46], cardiac senescence [47], diabetic cardiomyopathy [48] and atherosclerosis [49]. Nevertheless, how circDhx32 regulates cardiac I/R injury is still unclear. In this study, m^6^A-circRNA epitranscriptomic microarray analysis was used to screen for circRNAs with dysregulated m^6^A methylation in cardiac I/R model mice, and one of the new molecules that attracted our attention was mmu_circ_42424. We named this circRNA circDhx32 according to its host gene Dhx32 and performed subsequent experiments. The subsequent experiments confirmed that circDhx32, which has a circular structure and better stability than linear mRNA does, was upregulated in vivo and in vitro under pathological conditions. Interestingly, we found that circDhx32 was localized in both the nucleus and the cytoplasm but was transferred into the nucleus under H/R conditions, suggesting that it probably played a regulatory role in the nucleus. We then delivered an AAV9 vector carrying an NC-shRNA or circDhx32-shRNA fragment into the mouse heart and discovered that amelioration of cardiac function with circDhx32 silencing was concomitant with a reduction in infarct size and the release of biomarkers of cardiac injury, such as CK-MB and cTnT. Similarly, circDhx32 downregulation was of crucial importance in myocardial disarrangement and collagen deposition elimination, indicating that removed adverse cardiac remodeling upon I/R injury. Consistent with the in vivo results, after transfection with circDhx32 siRNA, detrimental effects, including decreased cell viability accompanied by increased LDH release, were observed in H/R-treated cardiomyocytes.

RNA m^6^A modification is the most common epigenetic modification and plays vital biological roles in various processes, including RNA splicing, localization, stability, and translation [50]. Previous studies reported that m^6^A modification is a dynamic and reversible modification mediated by m^6^A methyltransferases (METTL3, METTL14, and WTAP) and m^6^A demethylases (FTO and ALKBH5) [51]. In addition, the m^6^A reader YT521-B homology (YTH) domain-containing proteins (YTHDF1-3, YTHDC1 and YTHDC2) can recognize and bind to the m^6^A motif (RRACH, R = A/G/U, A = m^6^A, H = A/C/U) to influence target RNA function [52]. Dysregulation of m^6^A modification contributes to the development and progression of diverse diseases, especially cardiovascular diseases, such as arrhythmias, coronary heart disease and ischaemic heart failure [53]. Yang et al. reported that ALKBH5 is dramatically upregulated at the acute stage of myocardial infarction and prevents cardiac rupture by removing the m^6^A modification of ErbB4 mRNA [54]. YTHDF2 can transfer m^6^A RNA from the translation state to the degradation state; for example, our previous study revealed that YTHDF2 recognizes the m^6^A site on SLC7A11 mRNA to promote its degradation, thus effectively alleviating cardiac I/R injury [41]. Song et al. reported that YTHDC1 binds with circRNA3634 and accelerates its nuclear-to-cytoplasmic export in an m^6^A-dependent manner in antler chondrocytes [55]. In this study, we found that the level of ALKBH5 sharply increased in vivo and in vitro under pathological conditions, resulting in a significant reduction in the m^6^A modification level of circDhx32. Therefore, it was difficult for YTHDF2 and YTHDC1 to identify and bind with the m^6^A modification sites of circDhx32, which led to increased stability and accumulation in the nucleus. These findings explain the elevated expression and nuclear translocation of circDhx32 in I/R-treated mice and H/R-induced cardiomyocytes.

Numerous studies have revealed that circRNAs can bind to RNA-binding proteins to regulate downstream gene function, and Zhou et al. reported that circFIRRE binds to the HNRNPC protein to stabilize GLI2 mRNA, ultimately contributing to the progression of esophageal squamous cell carcinoma [56]. CircCwc27 suppresses Pur-α recruitment to the promoters of Alzheimer’s disease-related genes by interacting with Pur-α and trapping it in the cytoplasm, thereby accelerating cognitive dysfunction [57]. CircCDKN2B-AS1 has a carcinogenic effect on cervical cancer by binding to the IMP3 protein to stabilize HK2 mRNA [58]. Circ-Foxo3 blocks the antisenescent and antistress effects of ID-1, E2F1, FAK, and HIF1α by retaining them in the cytoplasm via conjugation, thus resulting in increased cellular senescence [47]. In our study, we filtered proteins that could bind to circDhx32 by RPISeq and predicted that FOXO1 would have a stronger interaction with circDhx32. Numerous investigations have shown that the function of FOXO1, a master transcription factor with complex activities, relies on the adjustment of downstream target factors such as apoptosis- and autophagy-associated genes, cell cycle arrest genes and immune regulators [59]. Zhang et al. reported that silencing FOXO1 reverses detrimental alterations, such as fibrosis, apoptosis, and excessive autophagy, in diabetic cardiomyopathy [60]. The inhibition of FOXO1 relieves vascular inflammatory reactions and plaque progression by decreasing the production and secretion of IL-1β, which provides new insights into atherosclerosis [61]. Cardiomyocyte-specific FOXO1 knockout ameliorates the detrimental phenotype and lessened cardiac steatosis in the context of heart failure with a preserved ejection fraction [62]. Our data confirmed the interaction between circDhx32 and FOXO1 via a RIP assay, and we unexpectedly found that circDhx32 influenced the nuclear translocation of FOXO1. CircLIFR-007 has been reported to promote hnRNPA1 nuclear export, thereby increasing the interaction between hnRNPA1 and YAP in the cytoplasm [63]. In addition, circDhx32 silencing decreased nuclear FOXO1 protein expression and increased its cytoplasmic accumulation under physiological conditions, whereas H/R increased its nuclear colocalization. Therefore, we speculated that increased circDhx32 bound to FOXO1, followed by transport into the nucleus to regulate downstream gene transcription after I/R and H/R.

Previous studies have shown that FOXO1 is a transcriptional activator of AdipoR1 [28], and JASPAR predicted that FOXO1 could bind to the AdipoR1 promoter. Interestingly, we found that FOXO1 could act as a transcription factor to promote AdipoR1 transcription via interaction with its promoter, while transfection of the circDhx32 plasmid reversed this change. ChIP and luciferase assays further confirmed these results. However, we speculated that the binding of circDhx32 to FOXO1 may change the FOXO1 conformation, resulting in its inability to function as a normal transcriptional regulator, which needs to be investigated in the future. AdipoR1 is considered one of the primary receptors for APN and targets the AMPK signaling pathway to regulate glucose and lipid metabolism, inflammation and oxidative stress [30]. An AdipoR1/2 dual agonist activated the AMPK pathway, thereby depressing inflammation and extracellular matrix deposition [43]. AMPK can suppress the activation of the NF-κB signaling pathway through multiple pathways [44]. Our studies revealed that circDhx32 impaired inflammation after cardiac I/R injury via the AdipoR1-AMPK-NF-κB signaling pathway. Silencing circDhx32 reversed the downregulation of AdipoR1 and the protein level of activated AMPK and reduced the increase in activated NF-κB and the release of proinflammatory cytokines against cardiac I/R impairment.

However, this research has several shortcomings: (1) There may be other reasons for circDhx32 upregulation in mice with cardiac I/R injury. (2) In addition to binding to RBPs, circDhx32 may regulate cardiac I/R injury in other ways, such as by sponging microRNAs.

## Conclusions

In conclusion, ALKBH5 acted as m^6^A eraser accompanied by the m^6^A writers YTHDF2 and YTHDC1 to promote high expression of circDhx32 in I/R-treated mice and H/R-induced cardiomyocytes and nuclear retention. CircDhx32 regulated the inflammatory response to cardiac I//R injury by targeting the AdipoR1-AMPK-NF-κB signaling pathway, which competed with AdipoR1 for FOXO1. These findings elucidate a novel molecular mechanism, and circDhx32 may be a novel potential therapeutic target for the clinical prevention and treatment of myocardial ischaemia‒reperfusion injury.

## Supplementary information

Supplementary Figure Legends

Supplementary Tables

Supplementary Figure 1

Supplementary Figure 2

Supplementary Figure 3

Supplementary Figure 4

Supplementary Figure 5

Supplementary Figure 6

western blots raw data
