# Supplementary material for: Circular RNA circDhx32 promotes cardiac inflammatory responses in mouse cardiac ischemia-reperfusion injury via binding to FOXO1 competed with AdipoR1
Source: Acta Pharmacol Sin. 2025 Jun 17;46(11):2924–37. doi: 10.1038/s41401-025-01593-9 (PMC12552442; doi:10.1038/s41401-025-01593-9)
Supplement: Supplementary file 1 — Supplementary Figure Legends [file 41401_2025_1593_MOESM1_ESM.docx]

**Supplementary Figure Legends**

**Fig S1 Screening of m^6^A modification-associated circRNAs and primary cardiomyocytes identification. a, b** Volcano plot and scatter plot showed differentially m^6^A modification circRNAs in mouse hearts from Sham and I/R group by m^6^A-circRNA epitranscriptomic microarray analysis. **c** The expression levels of differentially m^6^A modification circRNAs were determined by qRT-PCR in heart tissues with I/R injury. *n* = 6**‒**8. ^*^*P* < 0.05, ^**^*P* < 0.01 *vs*. Sham. *P* values were determined by unpaired *t* test. **d** Immunofluorescence analysis of α-actinin (Red) in primary cardiomyocytes. DAPI (Blue) was used to stain nuclei. Scale bar = 50 μm. *n* = 3. **e** The expression levels of differentially m^6^A modification circRNAs were determined by qRT-PCR in cardiomyocytes treated with H/R. *n* = 6**‒**8. ^*^*P* < 0.05, ^**^*P* < 0.01 *vs*. Ctrl. *P* values were determined by unpaired *t* test.

**Fig S2 CircDhx32 knockdown virus efficiency verification and the Evans blue and TTC stained slices diagram. a** GFP staining verified the circDhx32 knockdown efficiency. Scale bar = 100 µm. *n* = 3. **b** The mRNA expression level of Dhx32 in cardiac I/R mice after injected with AAV9 vector carrying an NC-shRNA or circDhx32-shRNA fragment. *n* = 6. **c, d** EF and FS were detected by ultrasound and statistics in mice after circDhx32 silencing. *n* = 9. *P* values were determined by unpaired *t* test. **e** Representative graphs of Evans blue/TTC staining in I/R-treated mouse hearts. Scale bar = 20 µm.

**Fig S3 CircDhx32 downregulation improves myocardial remodeling in I/R mouse hearts. a-c** Representative images of echocardiographs and EF and FS were used to evaluate cardiac function. *n* = 8. ^**^*P* < 0.01 *vs*. Sham+shNC-V; ^##^*P* < 0.01 *vs*. I/R 2w+shNC-V. One-way ANOVA followed by Dunnett’s multiple comparisons test was performed to evaluate significant differences. **d, e** Evans blue/TTC staining was performed to assess infarct size in I/R-treated mouse hearts upon circDxh32 silencing. Scale bar = 20 μm. *n* = 6. ^**^*P* < 0.01 *vs*. I/R 2w+shNC-V. One-way ANOVA followed by Dunnett’s multiple comparisons test was performed to evaluate significant differences. **f** Representative images of H&E staining of the myocardium. Scale bar = 20 μm. *n* = 7**‒**8. **g** Masson staining was used to exhibit the fibrotic level of myocardial tissue. Myofibers were stained in red and collagenous fibers were stained in blue. Scale bar = 500 μm. *n* = 7. ^**^*P* < 0.01 *vs*. Sham+shNC-V; ^##^*P* < 0.01 *vs*. I/R 2w+shNC-V. One-way ANOVA followed by Dunnett’s multiple comparisons test was performed to evaluate significant differences.

**Fig S4 Verification of silencing and overexpression related genes efficiency. a, b** The expression of ALKBH5 after transfection of si-ALKBH5. *n* = 4**‒**6. ^**^*P* < 0.01 *vs*. NC. *P* values were determined by unpaired *t* test. **c, d** qRT-PCR and Western blot verified the efficiency of YTHDF2 overexpression. *n* = 6. ^**^*P* < 0.01 *vs*. NC. *P* values were determined by unpaired *t* test. **e, f** Silencing YTHDF2 efficiency was verified by qRT-PCR and Western blot. *n* = 6. ^**^*P* < 0.01 *vs*. NC. *P* values were determined by unpaired *t* test. **g, h** qRT–PCR and Western blot verified the transfection efficiency of YTHDC1 overexpression plasmid. *n* = 6. ^**^*P* < 0.01 *vs*. NC. *P* values were determined by unpaired *t* test.

**Fig S5 The Gene Ontology molecular function of m^6^A-circRNA epitranscriptomic microarray analysis. a, b** Dot and pie plot exhibited the Gene Ontology molecular function of circRNAs with m^6^A dysregulation between Sham group mice and I/R group mice.

**Fig S6 Prediction of FOXO1 binding to circDhx32 and AdipoR1 promoter. a** RPISeq exhibited the interaction probabilities between circDhx32 and FOXO1. **b, c** Western blot analysis of the protein expression of FOXO1 in I/R mouse hearts and H/R cardiomyocytes. *n* = 6**‒**9. ^**^*P* < 0.01 *vs*. Sham, ^**^*P* < 0.01 *vs*. Ctrl. *P* values were determined by unpaired *t* test. **d, e** JASPAR predicted ten potential binding sites for transcription factor FOXO1 and promoter region of AdipoR1. **f, g** The mRNA and protein expression levels of FOXO1 in cardiomyocytes after transfecting with si-NC or si-FOXO1. *n* = 5**‒**6. ^*^*P* < 0.05, ^**^*P* < 0.01 *vs*. NC. *P* values were determined by unpaired *t* test. **h** qRT-PCR verified the efficiency of circDhx32 overexpression. *n* = 5**‒**6. ^**^*P* < 0.01 *vs*. NC. *P* values were determined by unpaired *t* test. **i** Overexpressing FOXO1 efficiency verified by Western blot. *n* = 6. ^**^*P* < 0.01 *vs*. NC. *P* values were determined by unpaired *t* test.
