# Supplementary material for: Circular RNA circDhx32 promotes cardiac inflammatory responses in mouse cardiac ischemia-reperfusion injury via binding to FOXO1 competed with AdipoR1
Source: Acta Pharmacol Sin. 2025 Jun 17;46(11):2924–37. doi: 10.1038/s41401-025-01593-9 (PMC12552442; doi:10.1038/s41401-025-01593-9)
Supplement: Supplementary file 2 — Supplementary Tables [file 41401_2025_1593_MOESM2_ESM.docx]

**Supplementary Table 1. The sequences of siRNA used for transfection**

| Gene name | Sense | Antisense |
| --- | --- | --- |
| circDhx32 | GAACAGGCCAGGUUCCUCATT | UGAGGAACCUGGCCUGUUCTT |
| YTHDF2 | CUAGAGAACAACGAGAAUATT | UAUUCUCGUUGUUCUCUAGTT |
| ALKBH5 | CCACCCAGCUAUGCUUCAGAUTT | AUCUGAAGCAUAGCUGGGUGGTT |
| FOXO1 | CGGAGGAUUGAACCAGUAUAATT | UUAUACUGGUUCAAUCCUCCGTT |

**Supplementary Table 2. Primer sequences used in qRT-PCR analysis**

| Gene name | Forward primer | Reverse primer |
| --- | --- | --- |
| GAPDH | AAGAAGGTGGTGAAGCAGGC | TCCACCACCCTGTTGCTGTA |
| 18S | CCTGGATACCGCAGCTAGGA | GCGGCGCAATACGAATGCCCC |
| YTHDF2 | TAGCCAACTGCGACACATTC | CACGACCTTGACGTTCCTTT |
| ALKBH5 | ACTGTGCTCAGTGGGTATGC | CCGGCGTTCCTTAATGTCCT |
| FOXO1 | ACCTGCTGAATGCCACTGAA | GAAAACAGGGGTTGCTGCTG |
| AdipoR1 | AGTCCGCGAGTGCTGTCT | TGTTCCCGCACCCAACTC |
| circDhx32 | CTTAAGCCAGCGGAAATGCAG | GCTGGTAATGGATGGAAAGGC |
| Dhx32 | AACGCTCTGTCAGCTCCTCT | GTTGCTGGGTTACCTTCGGT |
| IL-6 | AGCCAGAGTCCTTCAGAGAGA | GTGACTCCAGCTTATCTCTTGGT |
| TNF-α | ATCGGTCCCCAAAGGGATGA | TGGTTTGTGAGTGTGAGGGT |
| IL-1β | TGCCACCTTTTGACAGTGATG | GCAGCCCTTCATCTTTTGGG |
