# Supplementary figures and images for: Circular RNA circDhx32 promotes cardiac inflammatory responses in mouse cardiac ischemia-reperfusion injury via binding to FOXO1 competed with AdipoR1
Source: Acta Pharmacol Sin. 2025 Jun 17;46(11):2924–37. doi: 10.1038/s41401-025-01593-9 (PMC12552442; doi:10.1038/s41401-025-01593-9)

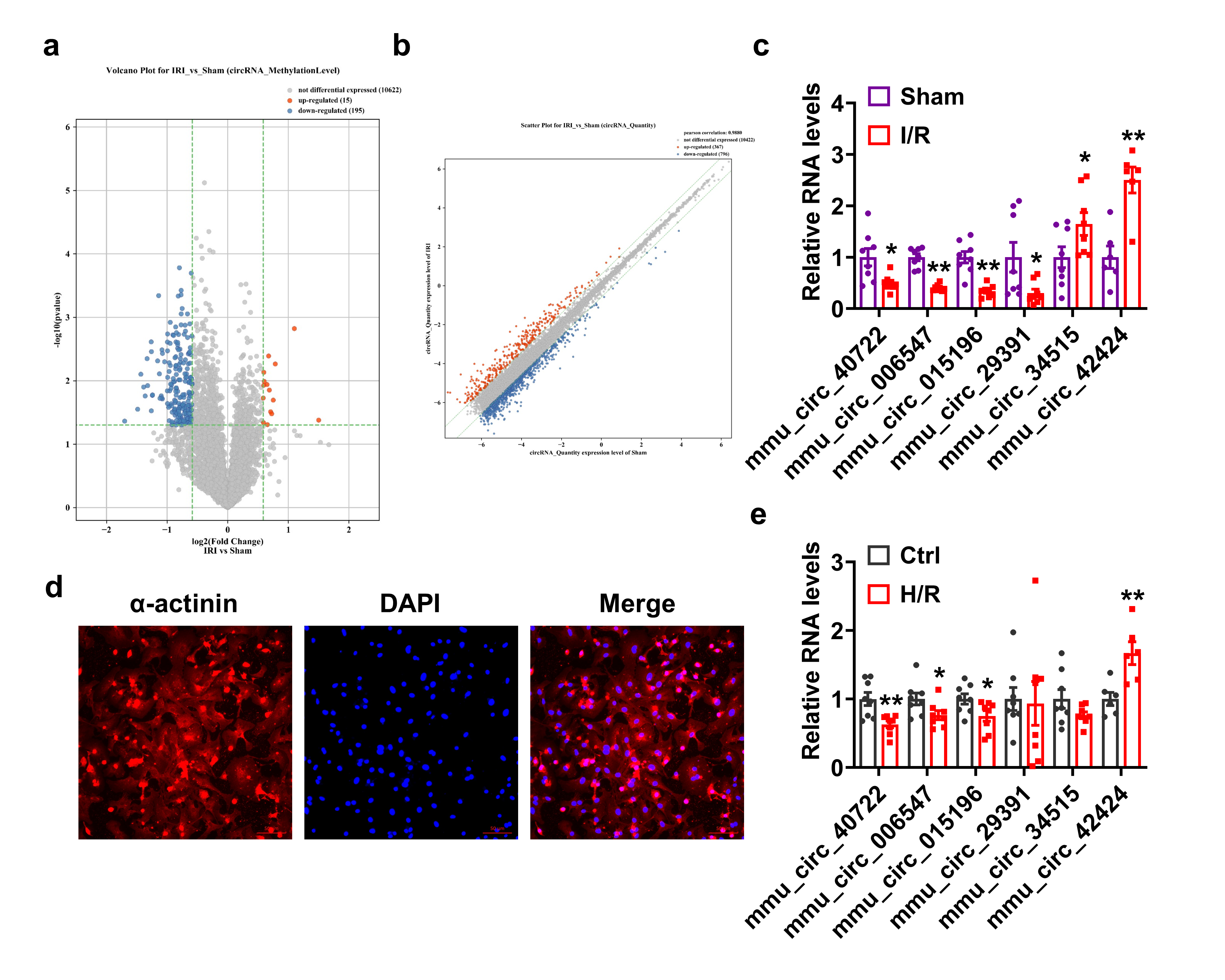

Supplement: Supplementary file 3 — Supplementary Figure 1 [file 41401_2025_1593_MOESM3_ESM.tif]

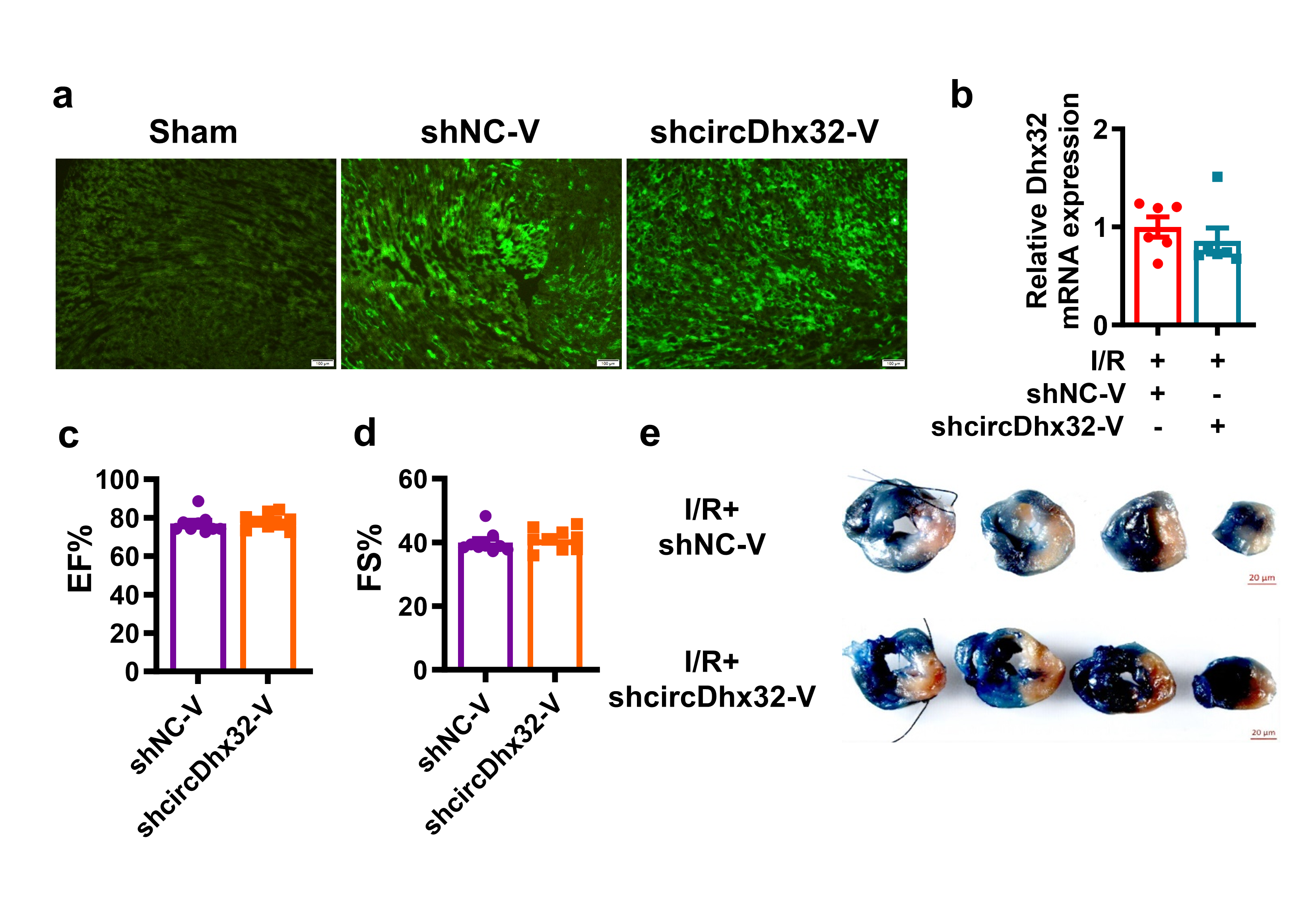

Supplement: Supplementary file 4 — Supplementary Figure 2 [file 41401_2025_1593_MOESM4_ESM.tif]

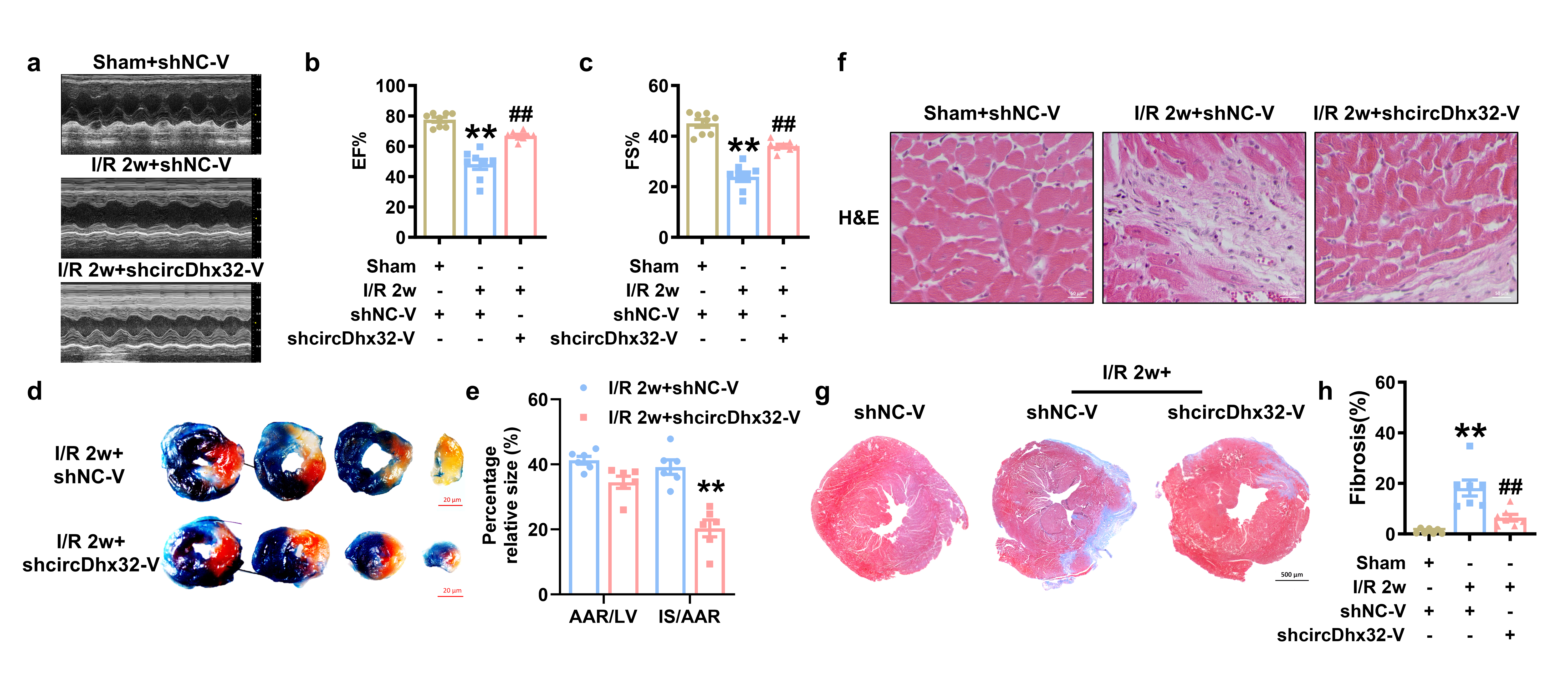

Supplement: Supplementary file 5 — Supplementary Figure 3 [file 41401_2025_1593_MOESM5_ESM.tif]

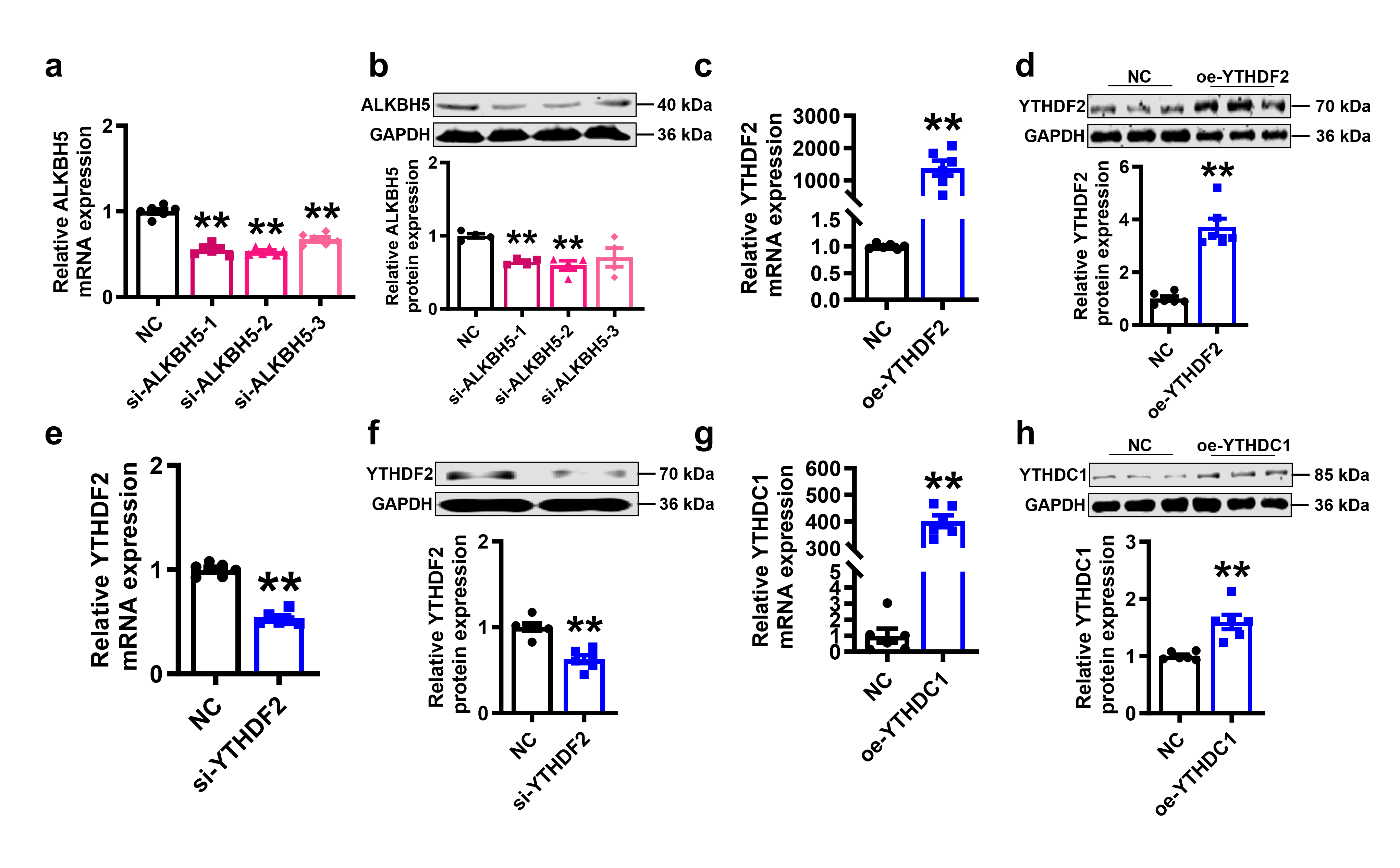

Supplement: Supplementary file 6 — Supplementary Figure 4 [file 41401_2025_1593_MOESM6_ESM.tif]

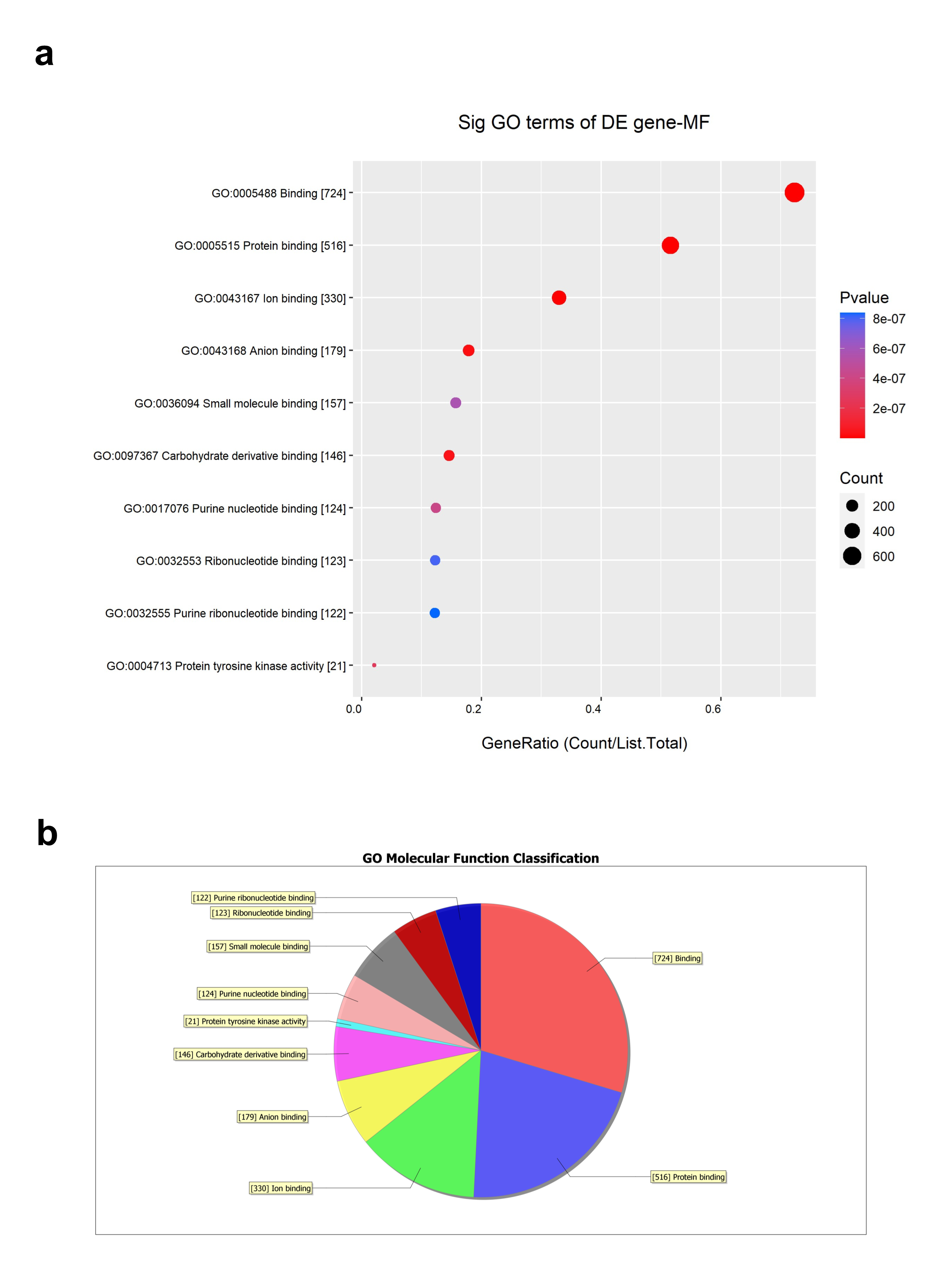

Supplement: Supplementary file 7 — Supplementary Figure 5 [file 41401_2025_1593_MOESM7_ESM.tif]

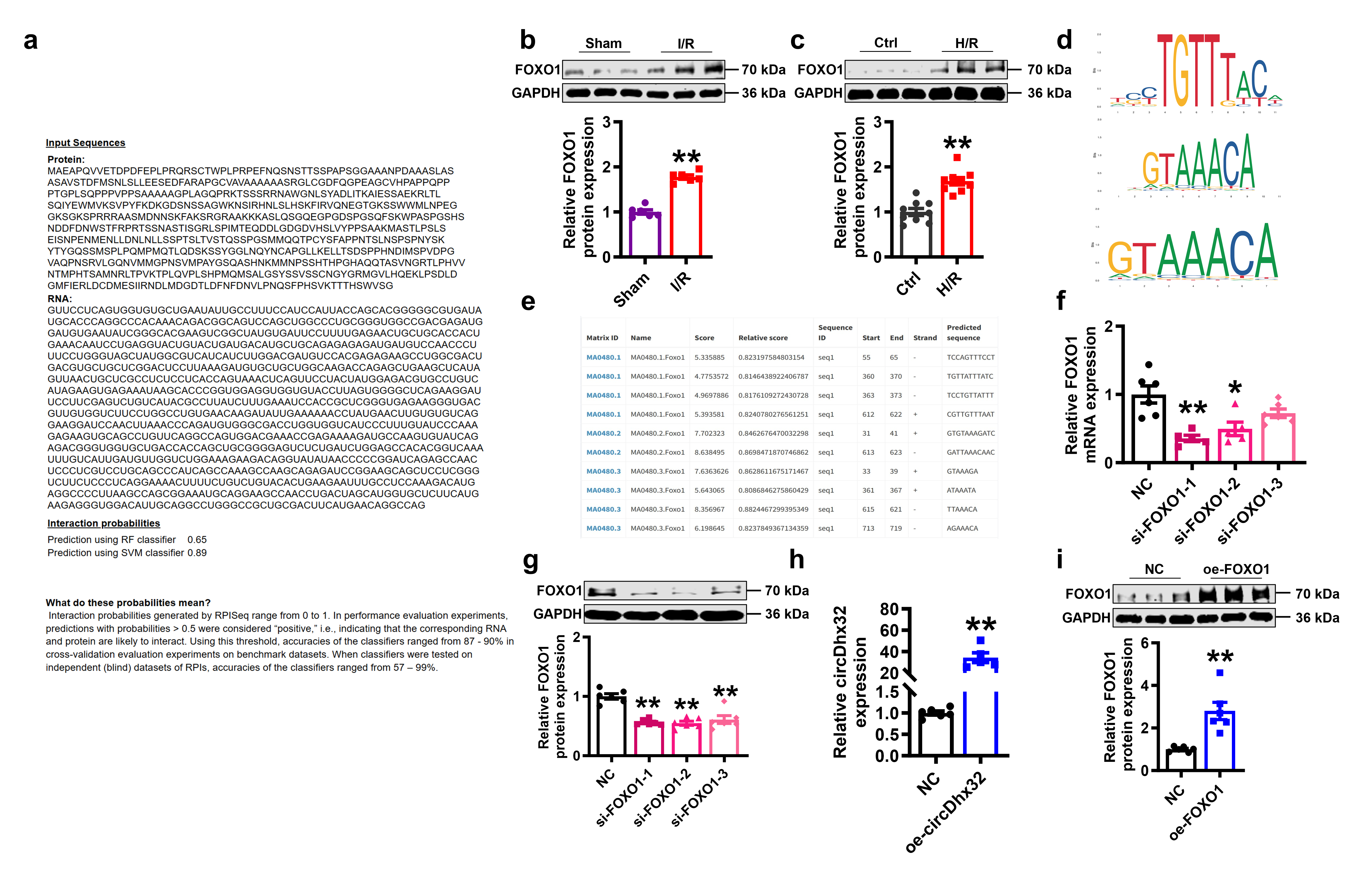

Supplement: Supplementary file 8 — Supplementary Figure 6 [file 41401_2025_1593_MOESM8_ESM.tif]
