# Supplementary material for: Circular RNA circDhx32 promotes cardiac inflammatory responses in mouse cardiac ischemia-reperfusion injury via binding to FOXO1 competed with AdipoR1
Source: Acta Pharmacol Sin. 2025 Jun 17;46(11):2924–37. doi: 10.1038/s41401-025-01593-9 (PMC12552442; doi:10.1038/s41401-025-01593-9)
Supplement: Supplementary file 9 — western blots raw data [file 41401_2025_1593_MOESM9_ESM.pptx]

## Slide 1
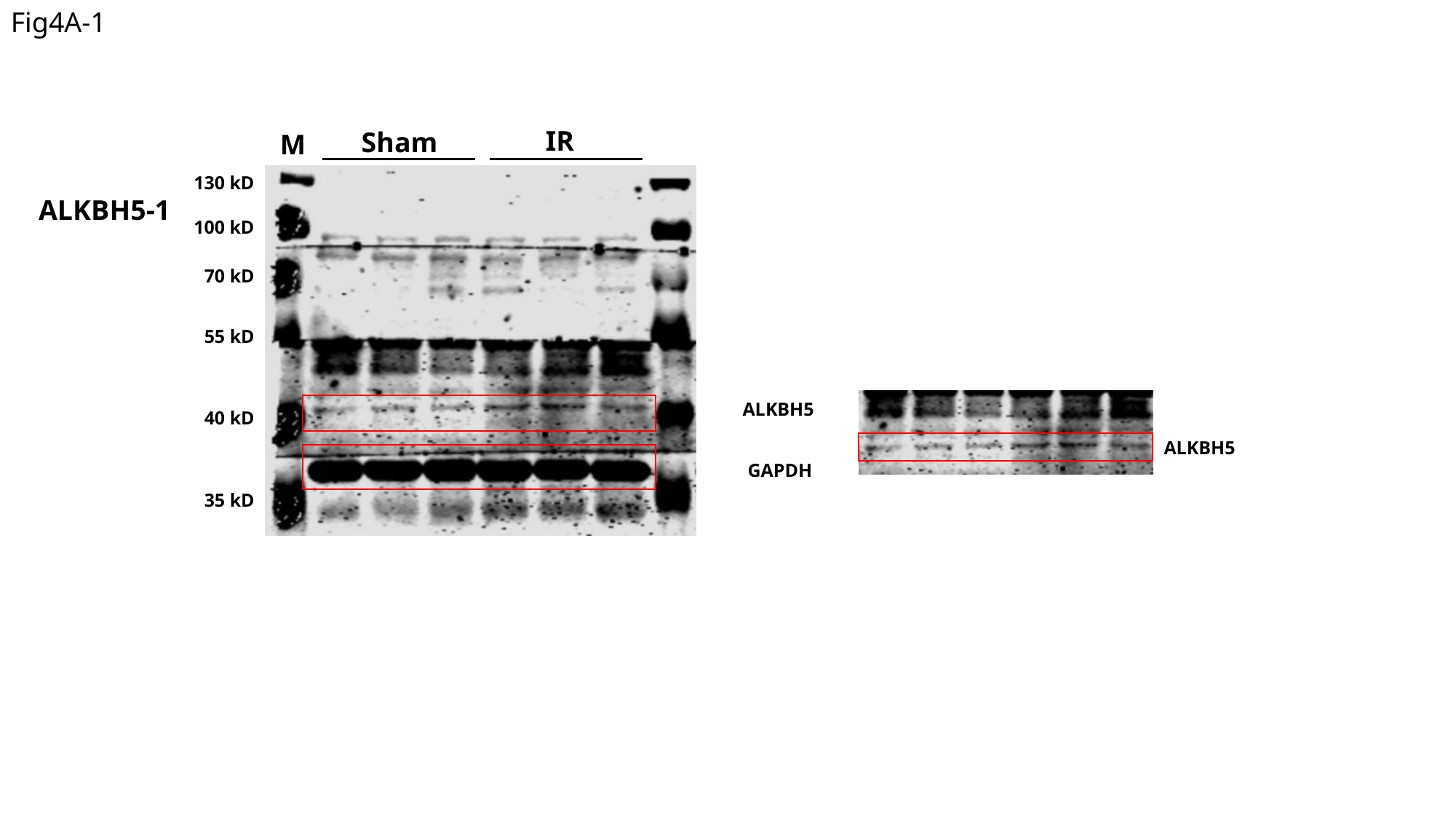

Fig4A-1
IR
Sham
M
130 kD
ALKBH5-1
100 kD
70 kD
55 kD
ALKBH5
40 kD
ALKBH5
GAPDH
35 kD

## Slide 2
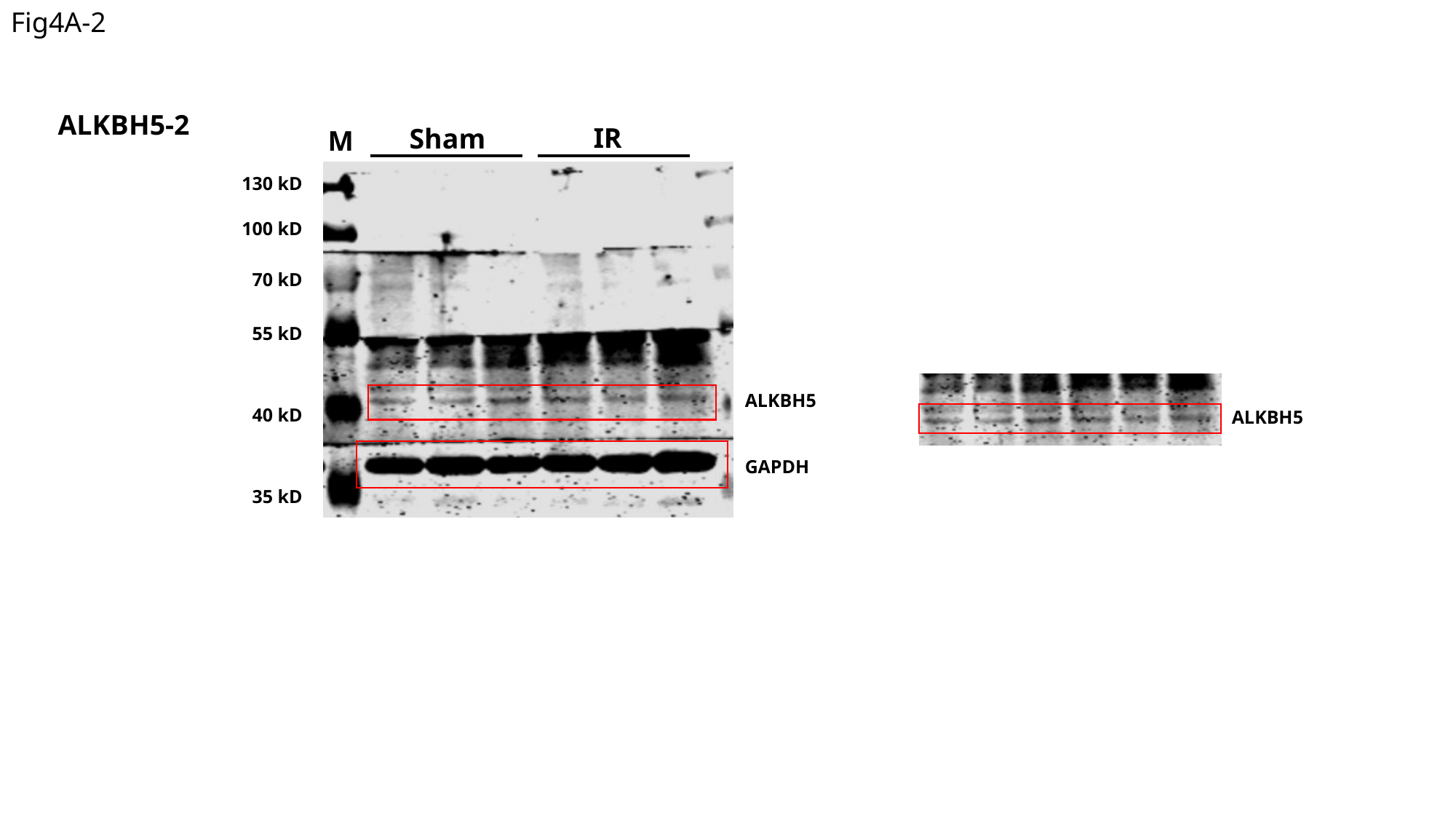

Fig4A-2
ALKBH5-2
IR
Sham
M
130 kD
100 kD
70 kD
55 kD
ALKBH5
40 kD
ALKBH5
GAPDH
35 kD

## Slide 3
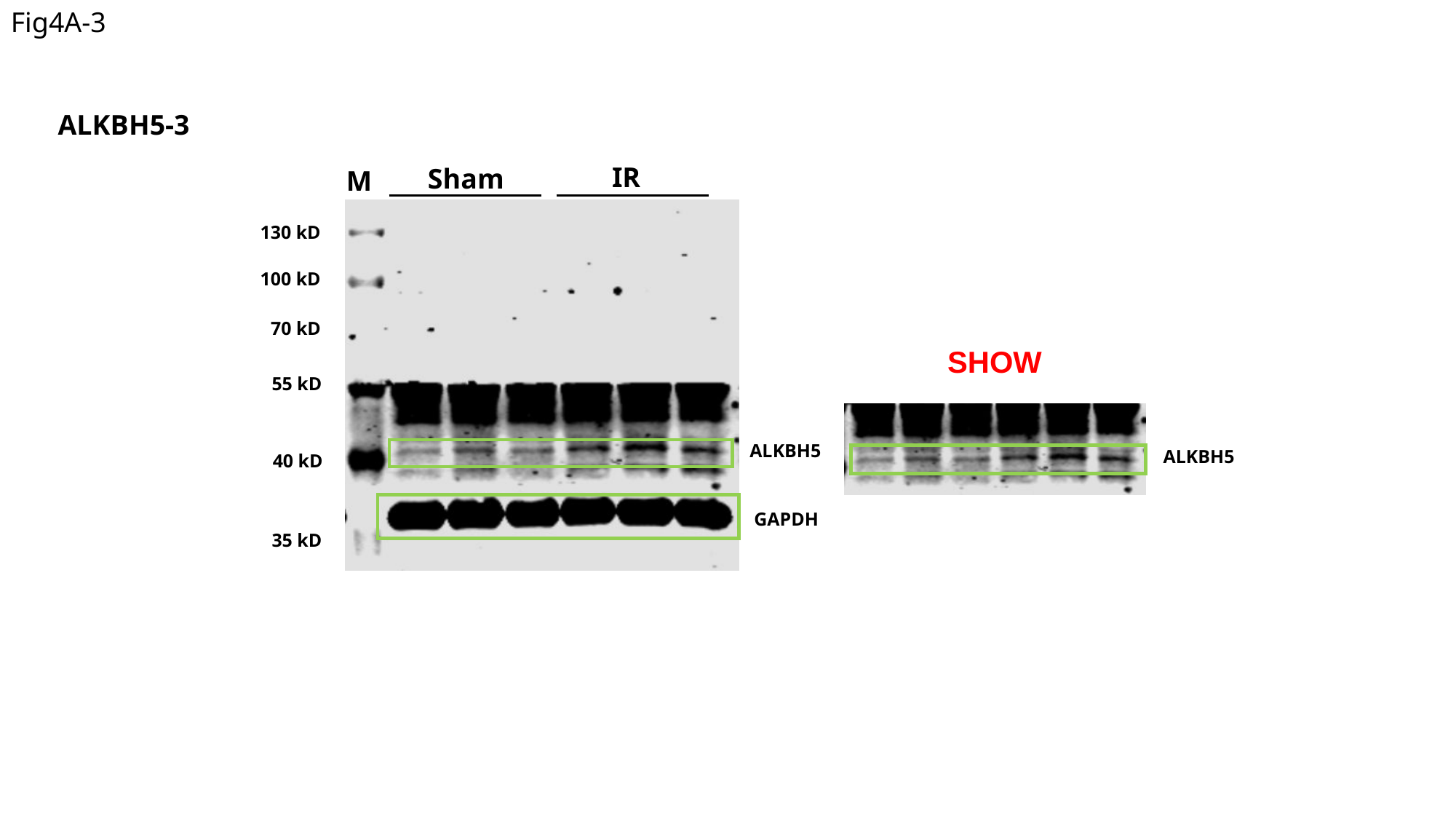

Fig4A-3
ALKBH5-3
IR
Sham
M
130 kD
100 kD
70 kD
SHOW
55 kD
ALKBH5
ALKBH5
40 kD
GAPDH
35 kD

## Slide 4
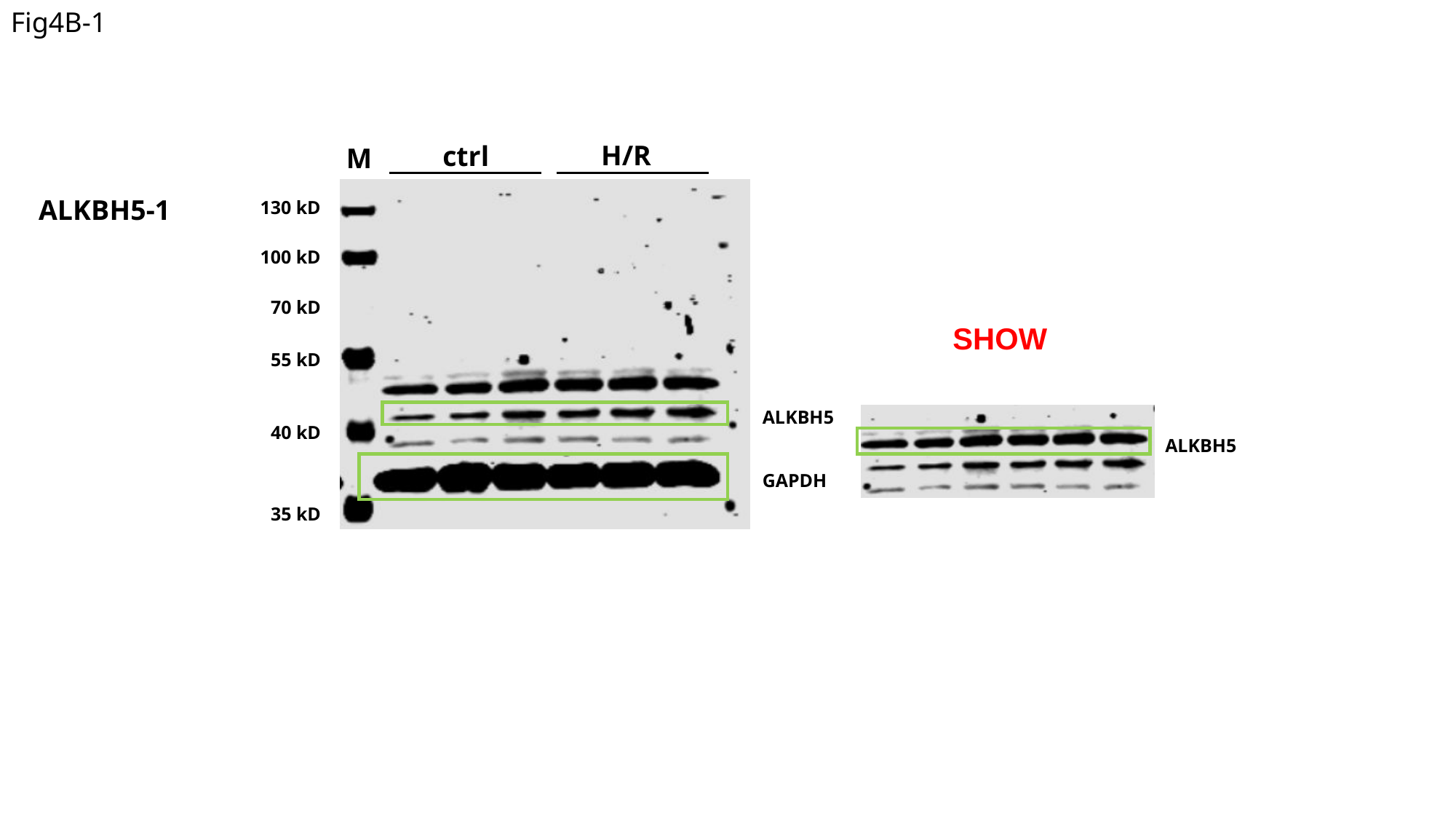

Fig4B-1
H/R
ctrl
M
ALKBH5-1
130 kD
100 kD
70 kD
SHOW
55 kD
ALKBH5
40 kD
ALKBH5
GAPDH
35 kD

## Slide 5
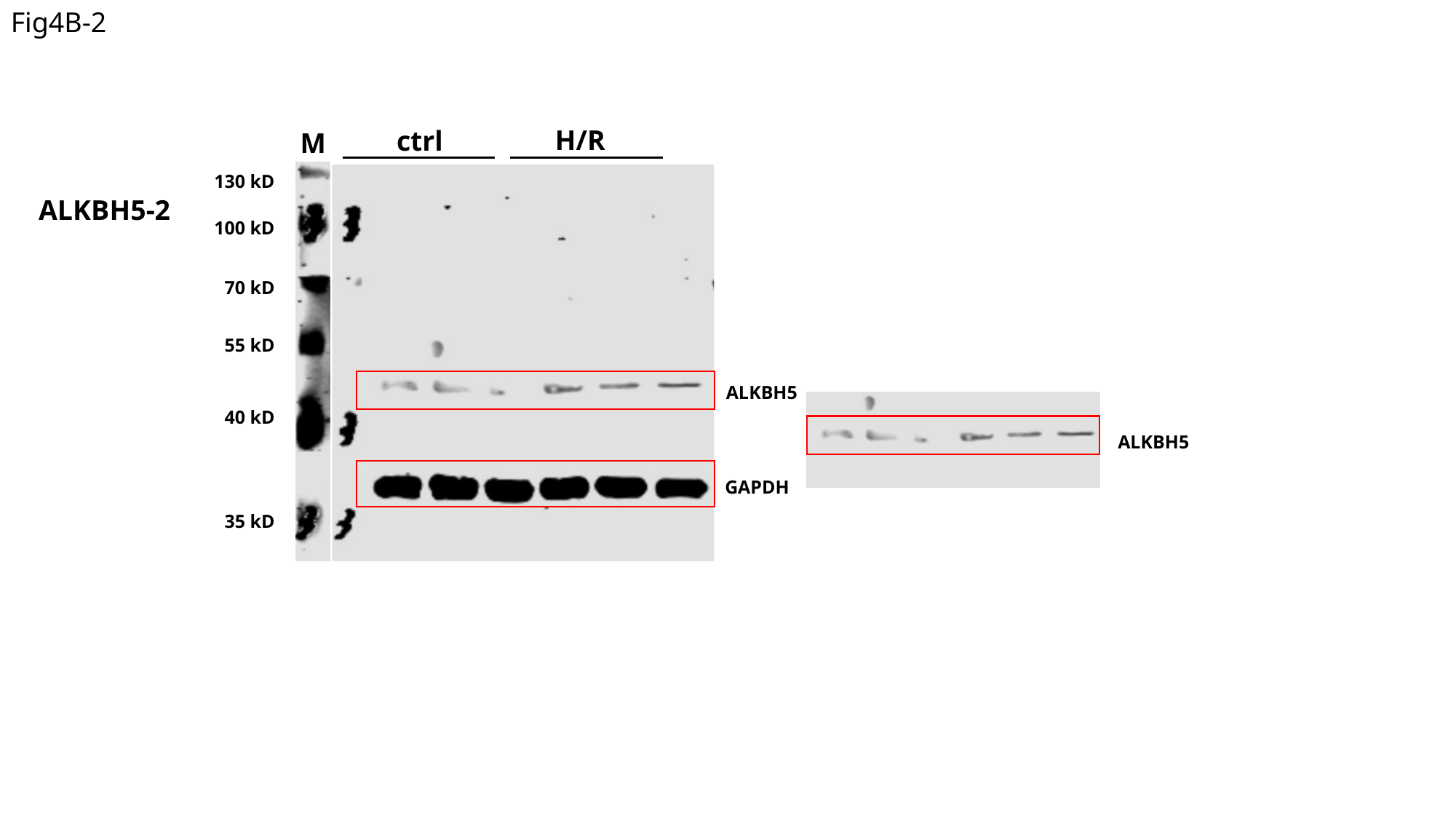

Fig4B-2
H/R
ctrl
M
130 kD
ALKBH5-2
100 kD
70 kD
55 kD
ALKBH5
40 kD
ALKBH5
GAPDH
35 kD

## Slide 6
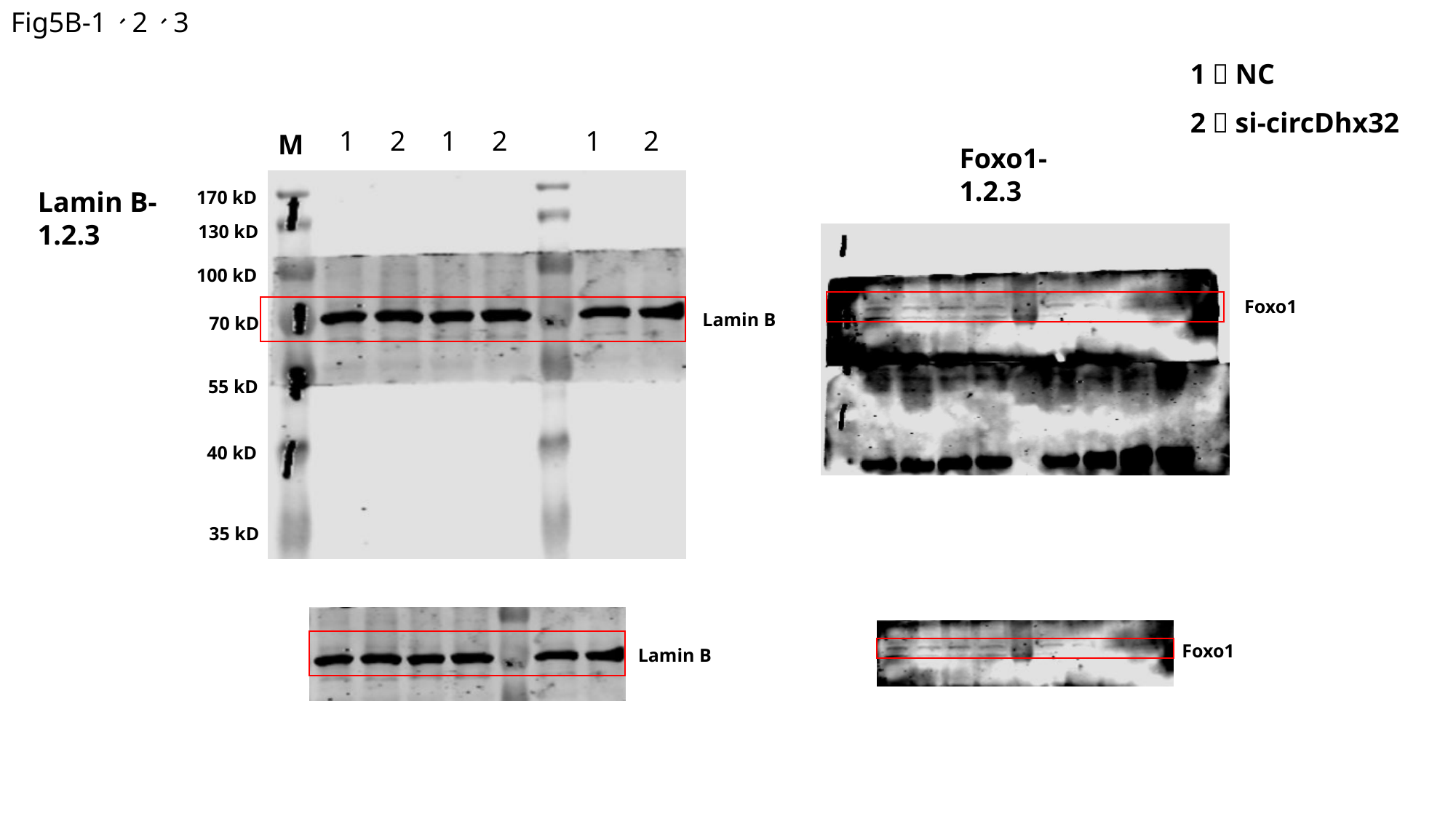

Fig5B-1、2、3
1：NC
2：si-circDhx32
 1 2 1 2 1 2
M
170 kD
130 kD
100 kD
Lamin B
70 kD
55 kD
40 kD
35 kD
Foxo1-1.2.3
Lamin B-1.2.3
Foxo1
Foxo1
Lamin B

## Slide 7
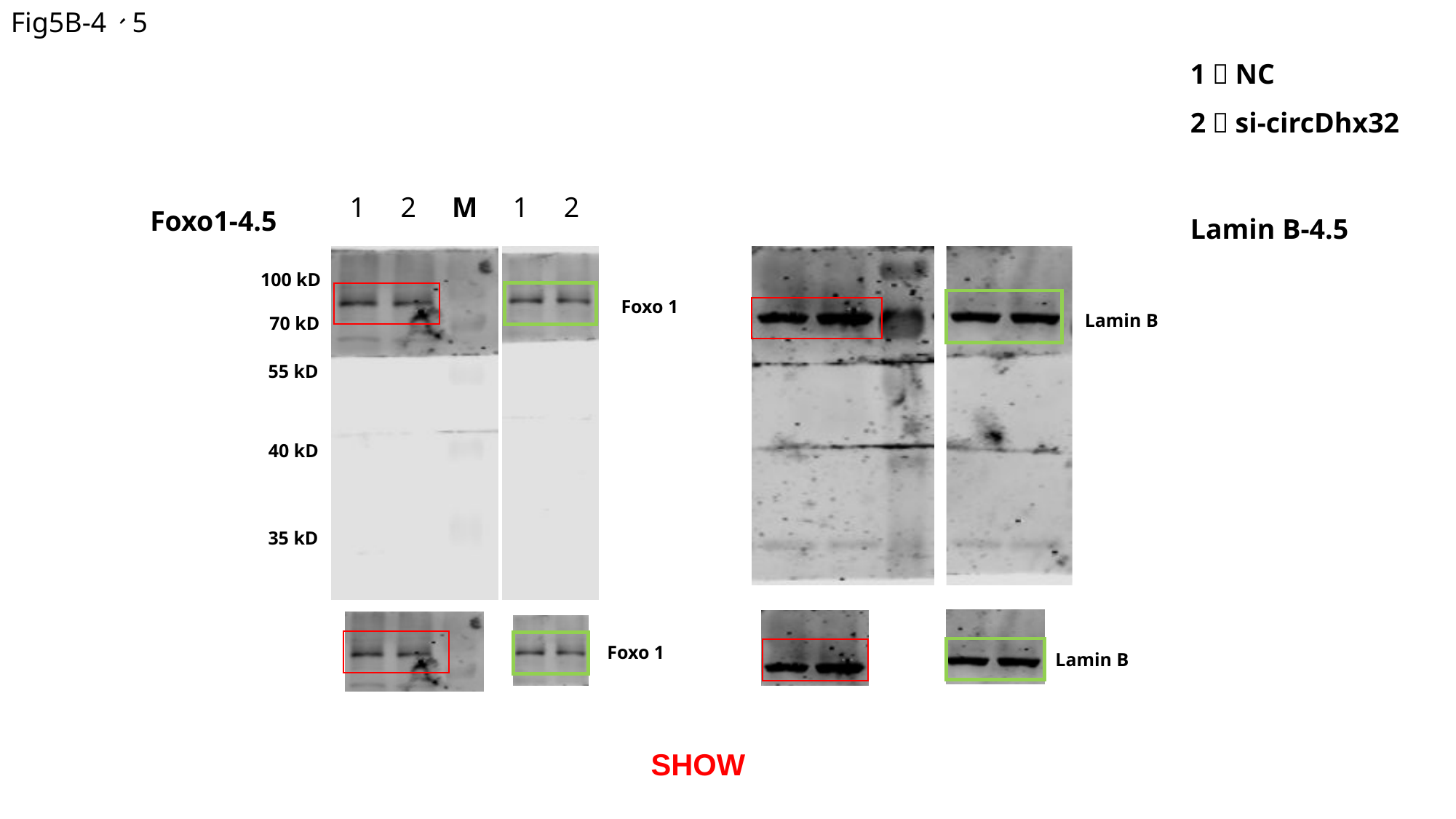

Fig5B-4、5
1：NC
2：si-circDhx32
 1 2 M 1 2
Foxo1-4.5
Lamin B-4.5
100 kD
Foxo 1
Lamin B
70 kD
55 kD
40 kD
35 kD
Foxo 1
Lamin B
SHOW

## Slide 8
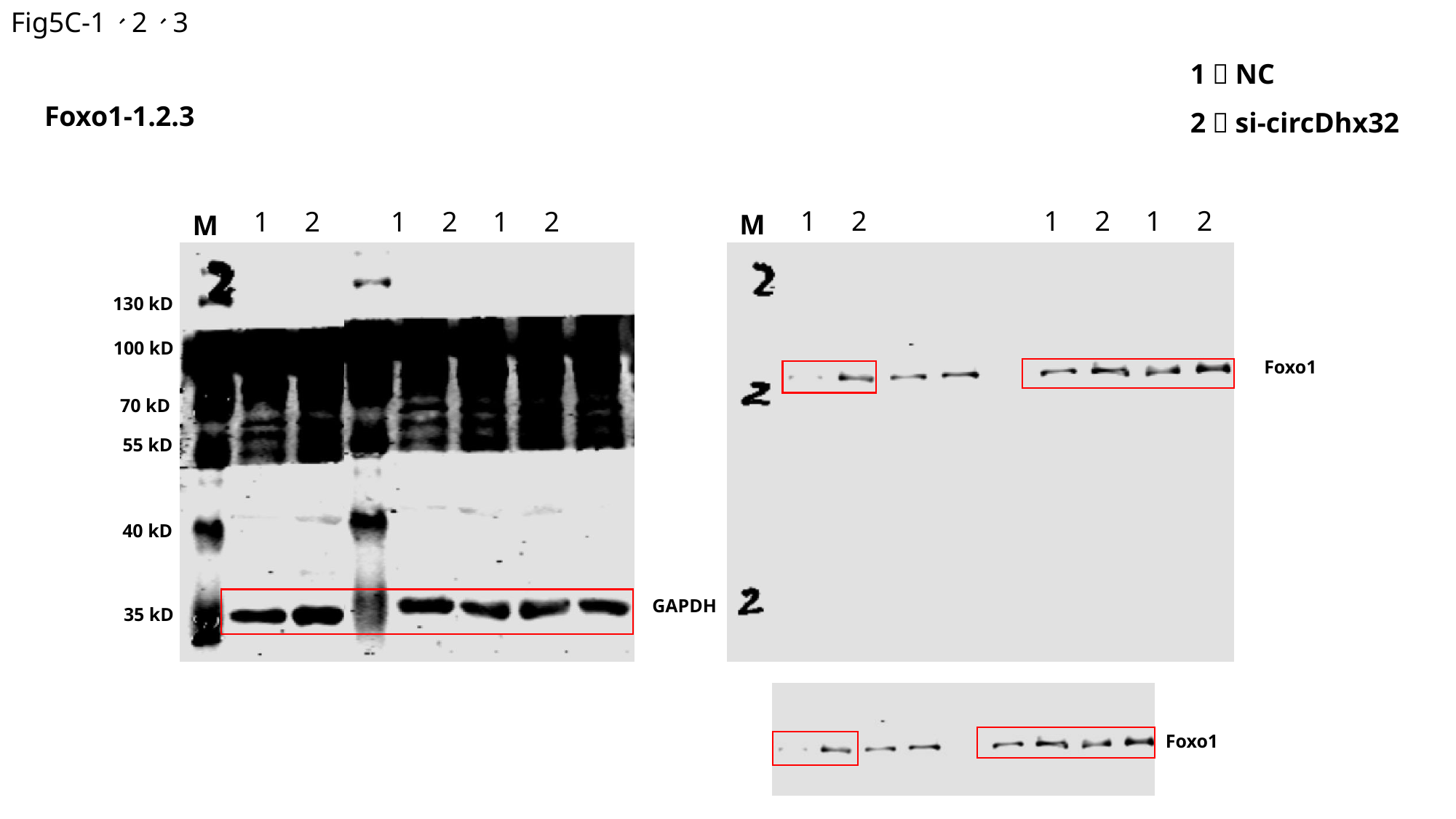

Fig5C-1、2、3
1：NC
2：si-circDhx32
Foxo1-1.2.3
 1 2 1 2 1 2
 1 2 1 2 1 2
M
M
130 kD
100 kD
Foxo1
70 kD
55 kD
40 kD
GAPDH
35 kD
Foxo1

## Slide 9
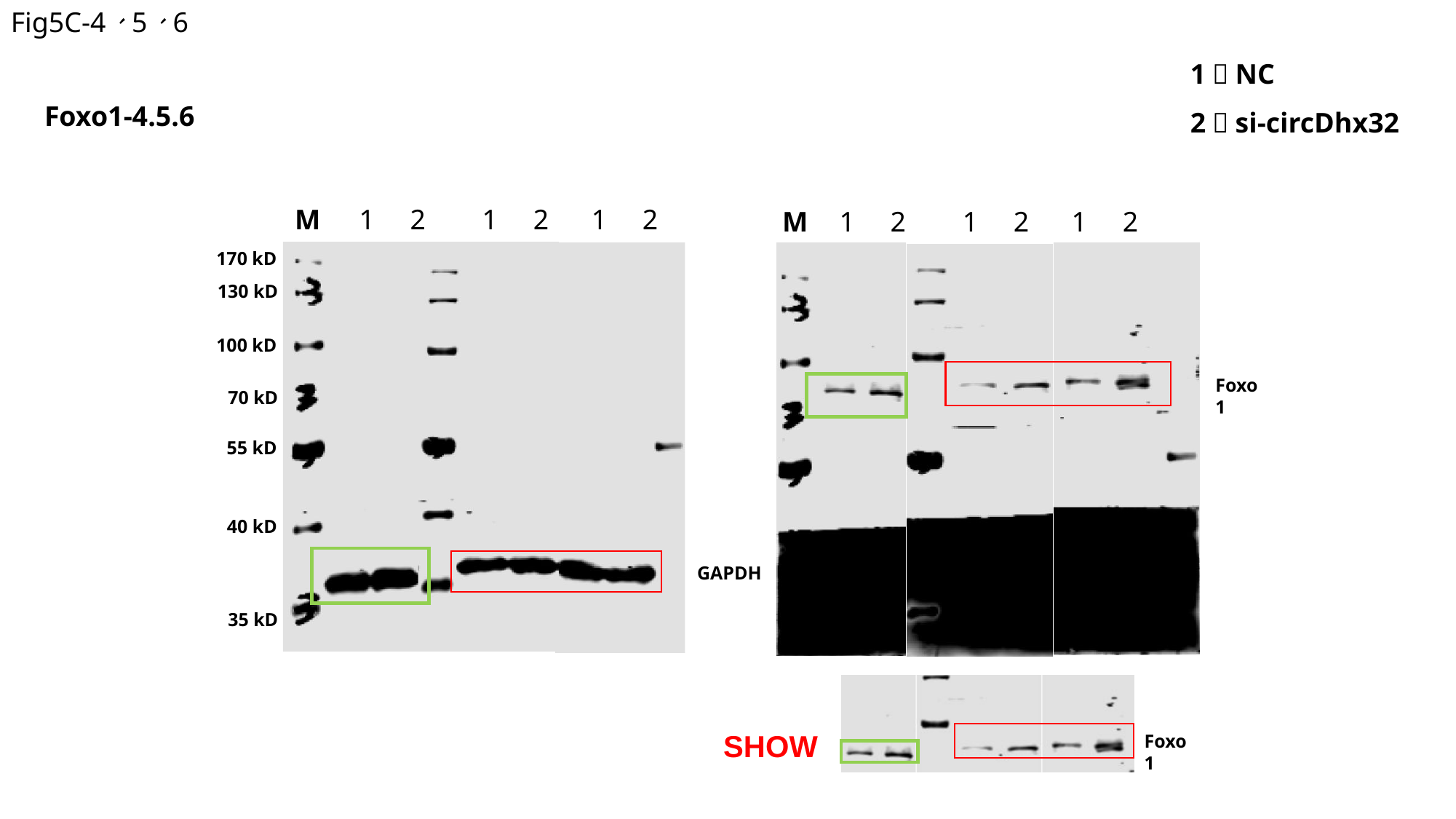

Fig5C-4、5、6
1：NC
2：si-circDhx32
Foxo1-4.5.6
 1 2 1 2 1 2
M
 1 2 1 2 1 2
M
170 kD
130 kD
100 kD
Foxo1
70 kD
55 kD
40 kD
GAPDH
35 kD
SHOW
Foxo1

## Slide 10
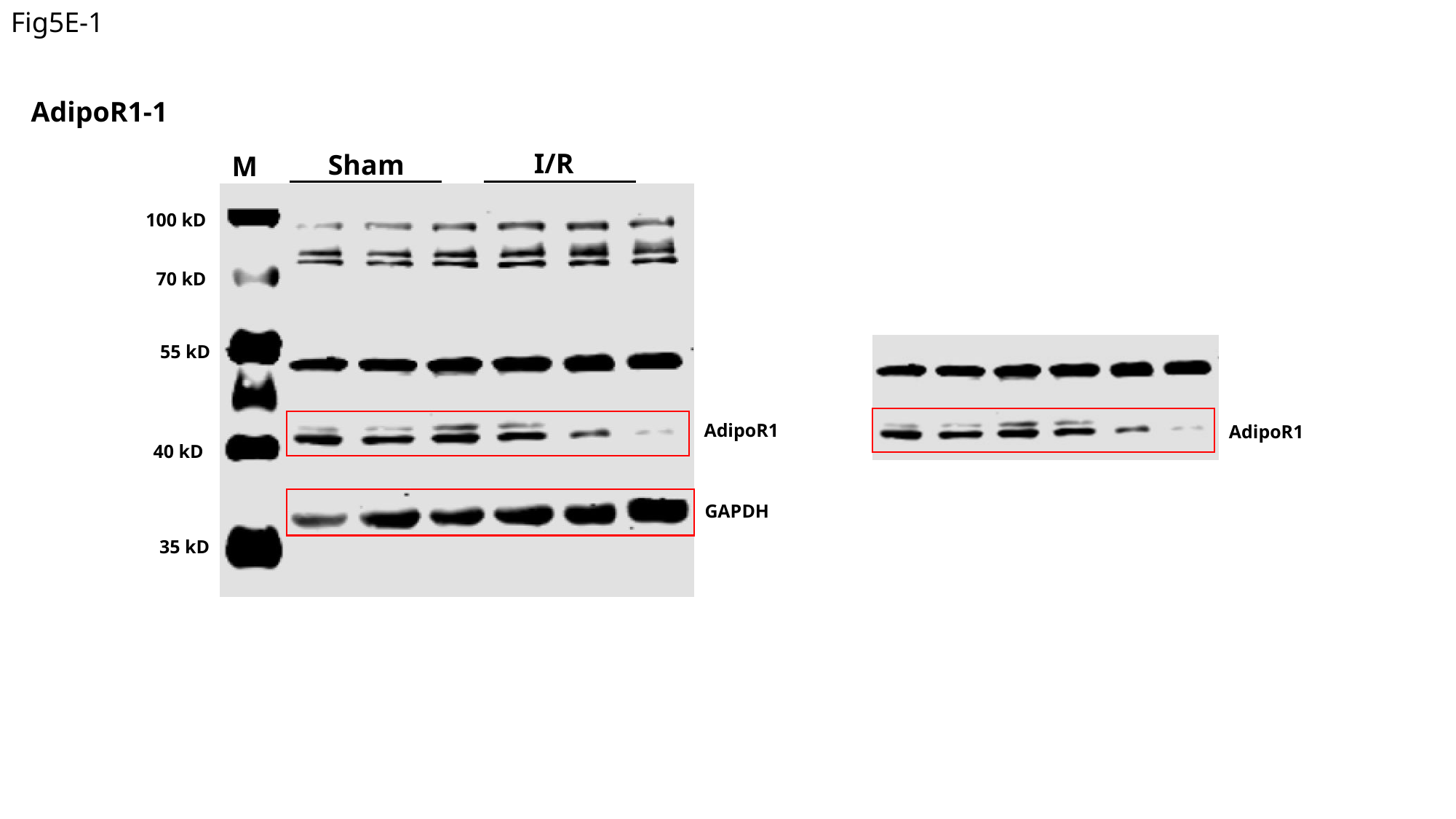

Fig5E-1
AdipoR1-1
I/R
Sham
M
100 kD
70 kD
55 kD
AdipoR1
AdipoR1
40 kD
GAPDH
35 kD

## Slide 11
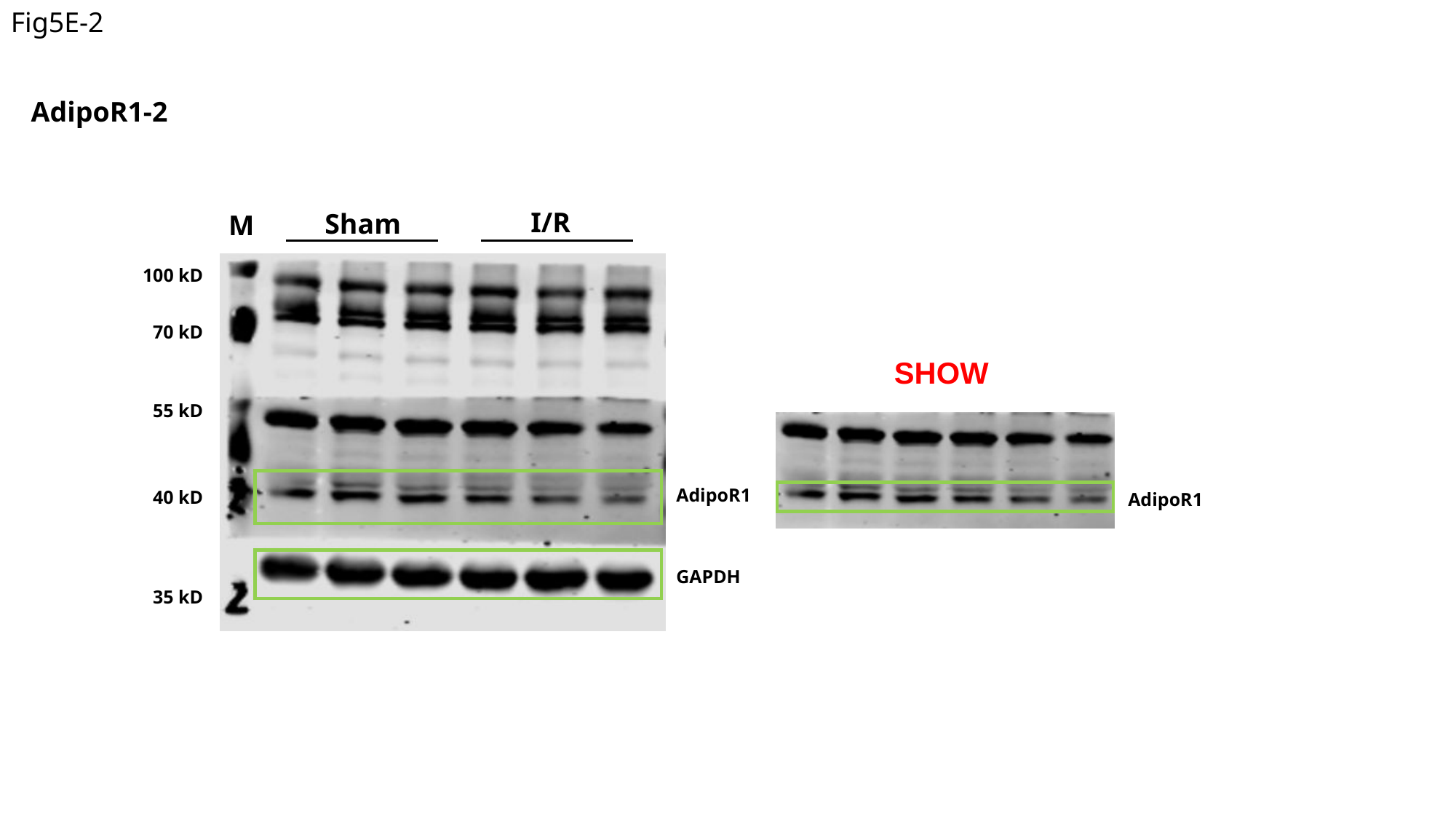

Fig5E-2
AdipoR1-2
I/R
Sham
M
100 kD
70 kD
SHOW
55 kD
AdipoR1
40 kD
AdipoR1
GAPDH
35 kD

## Slide 12
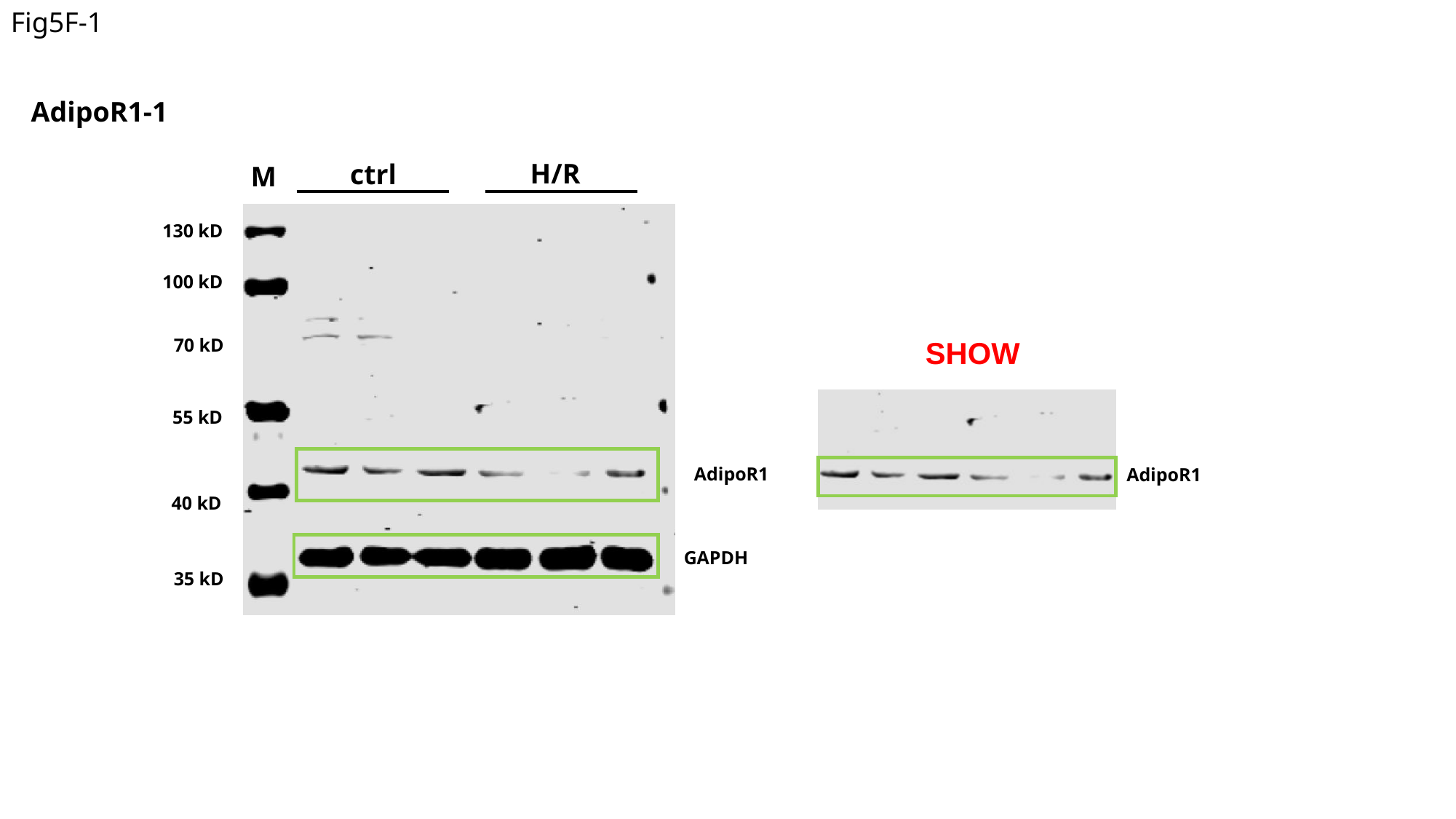

Fig5F-1
AdipoR1-1
H/R
ctrl
M
130 kD
100 kD
70 kD
SHOW
55 kD
AdipoR1
AdipoR1
40 kD
GAPDH
35 kD

## Slide 13
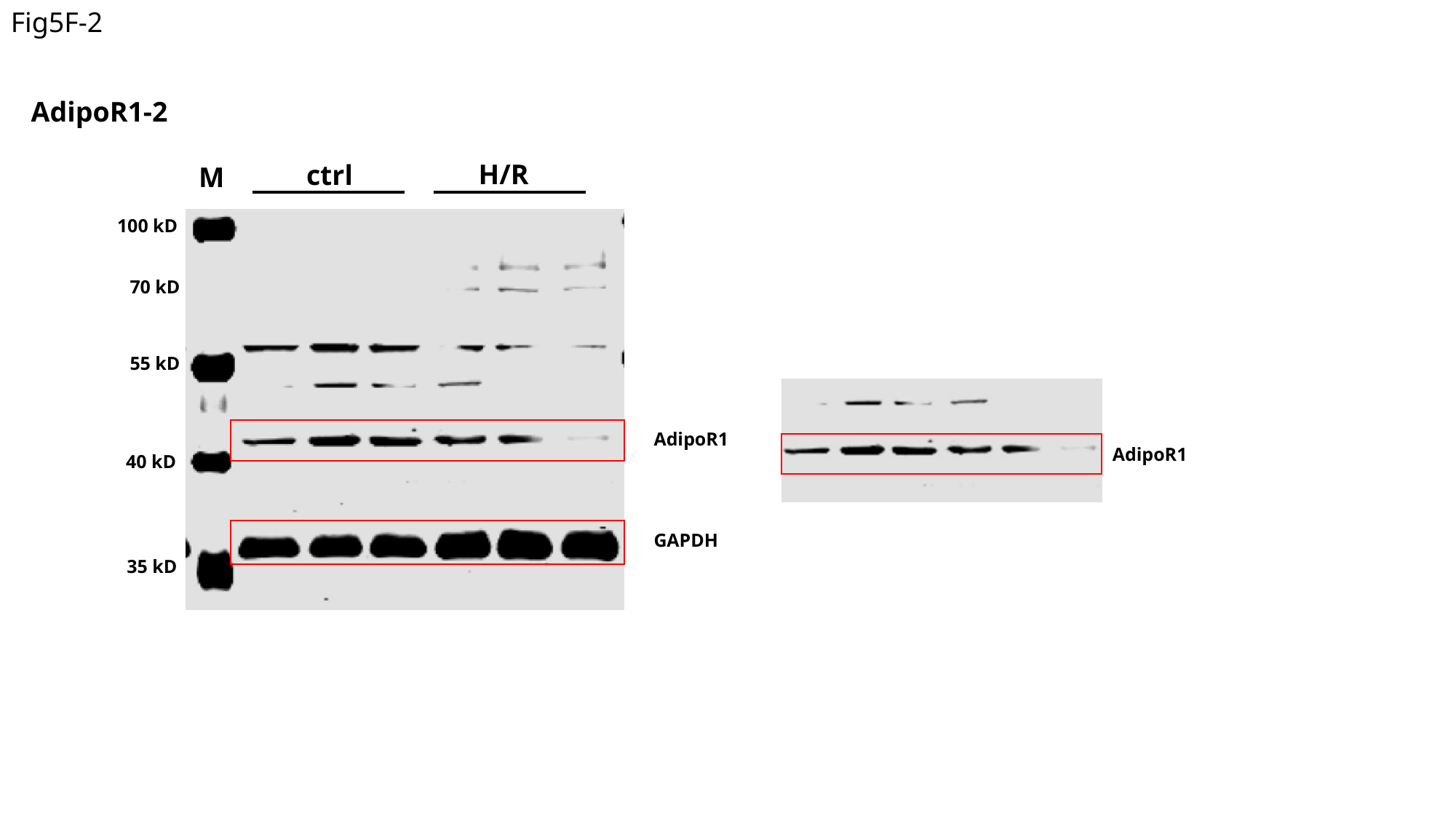

Fig5F-2
AdipoR1-2
H/R
ctrl
M
100 kD
70 kD
55 kD
AdipoR1
AdipoR1
40 kD
GAPDH
35 kD

## Slide 14
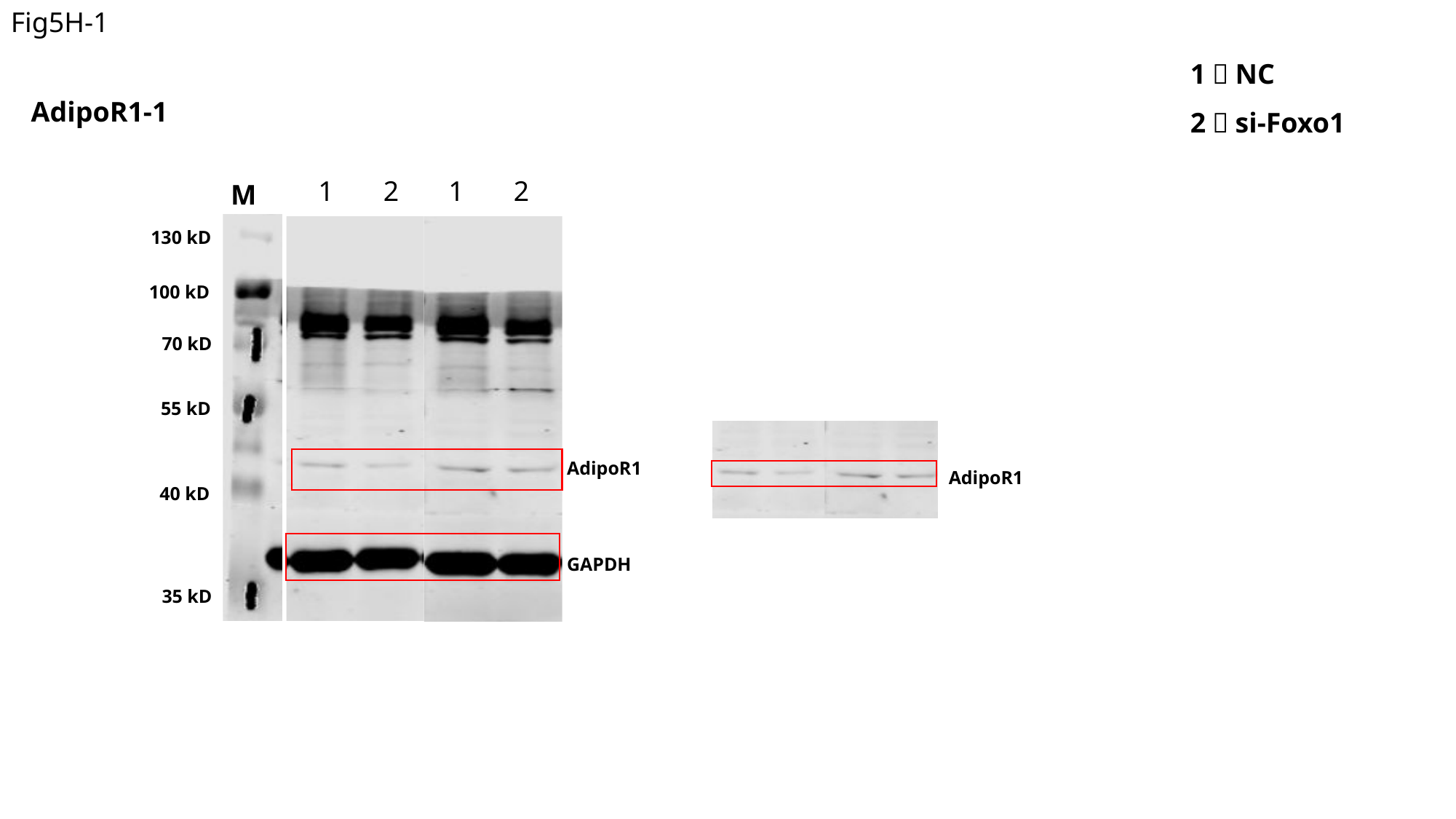

Fig5H-1
1：NC
2：si-Foxo1
AdipoR1-1
 1 2 1 2
M
130 kD
100 kD
70 kD
55 kD
AdipoR1
AdipoR1
40 kD
GAPDH
35 kD

## Slide 15
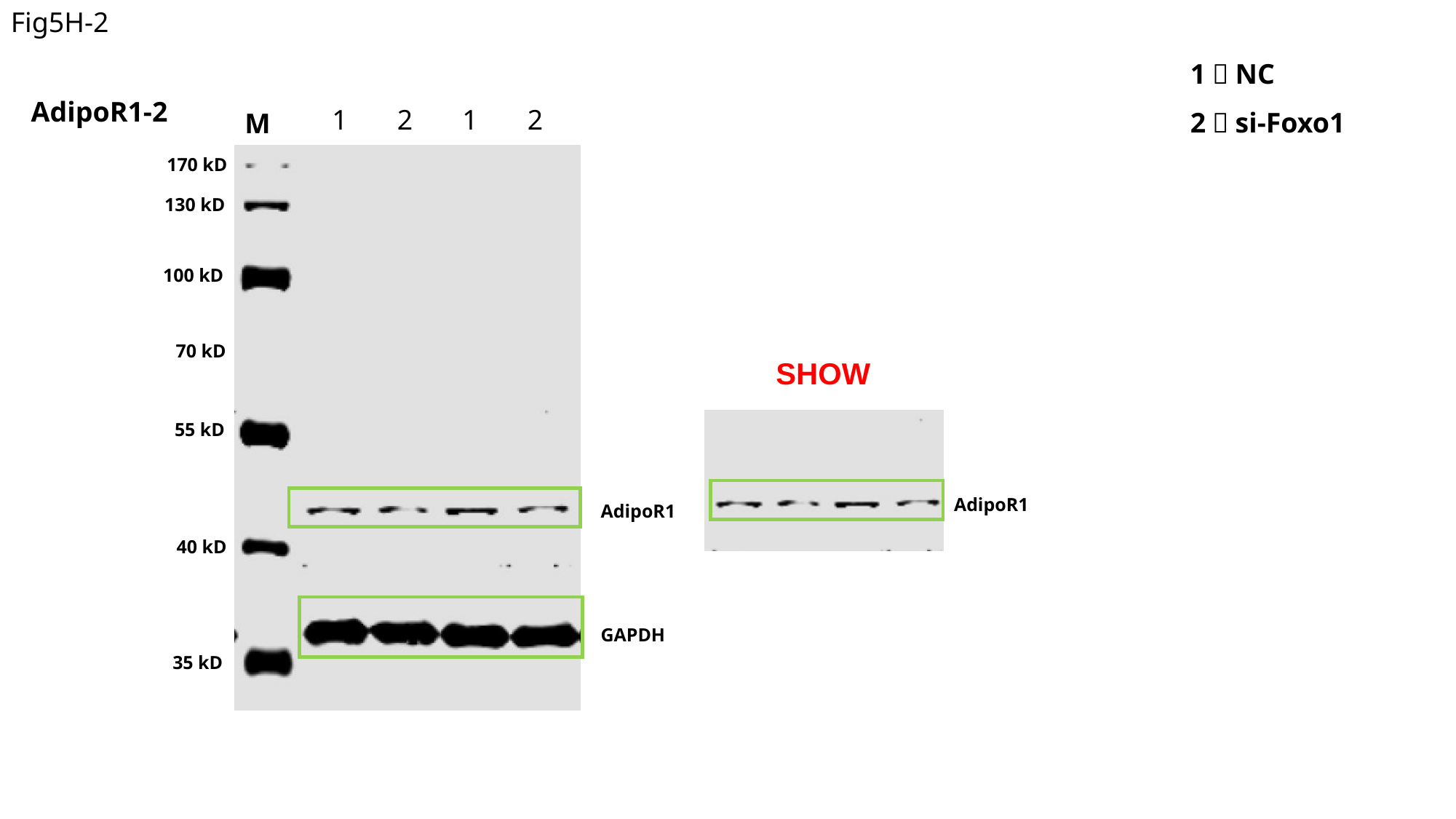

Fig5H-2
1：NC
2：si-Foxo1
AdipoR1-2
 1 2 1 2
M
170 kD
130 kD
100 kD
70 kD
SHOW
55 kD
AdipoR1
AdipoR1
40 kD
GAPDH
35 kD

## Slide 16
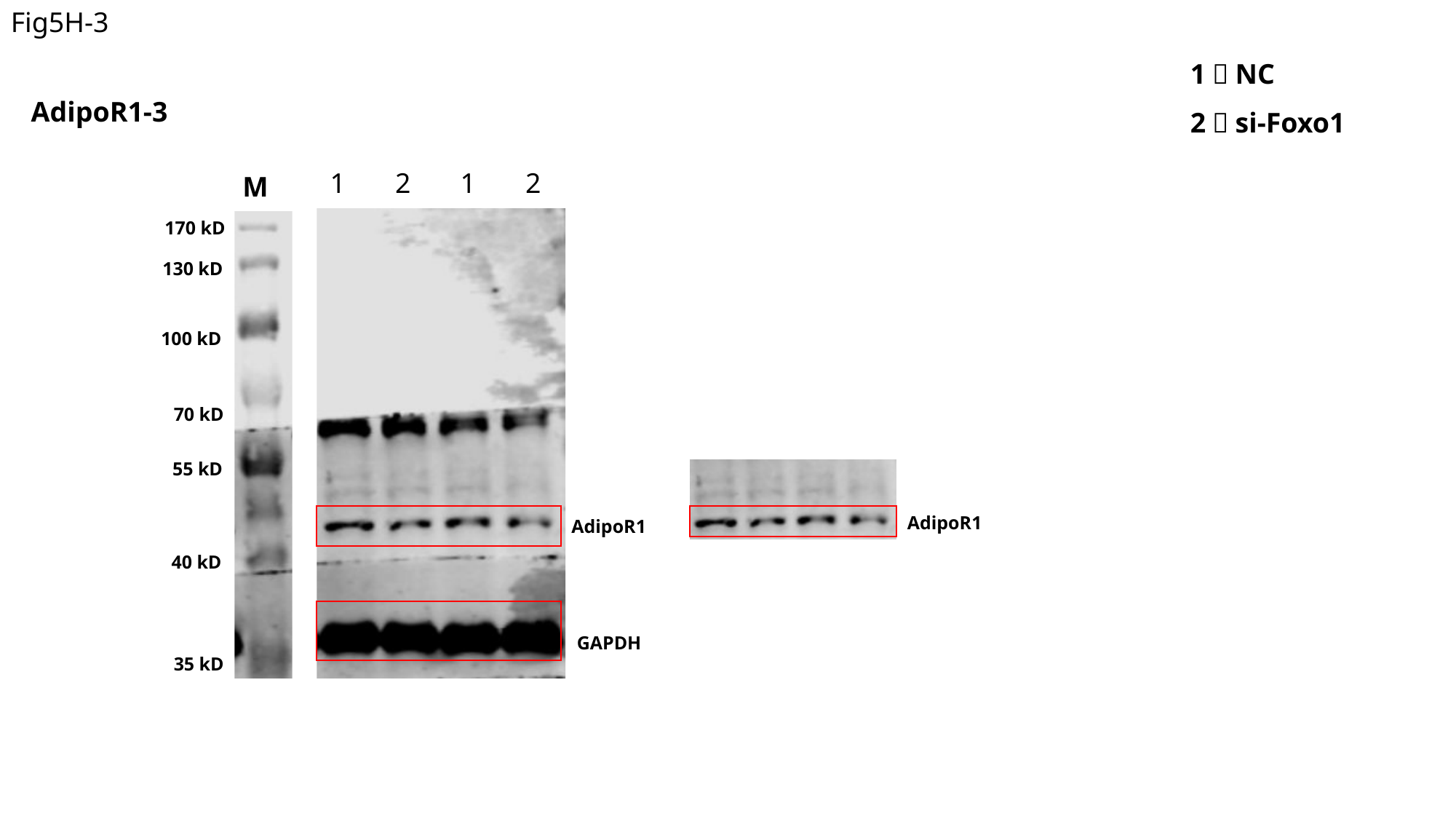

Fig5H-3
1：NC
2：si-Foxo1
AdipoR1-3
 1 2 1 2
M
170 kD
130 kD
100 kD
70 kD
55 kD
AdipoR1
AdipoR1
40 kD
GAPDH
35 kD

## Slide 17
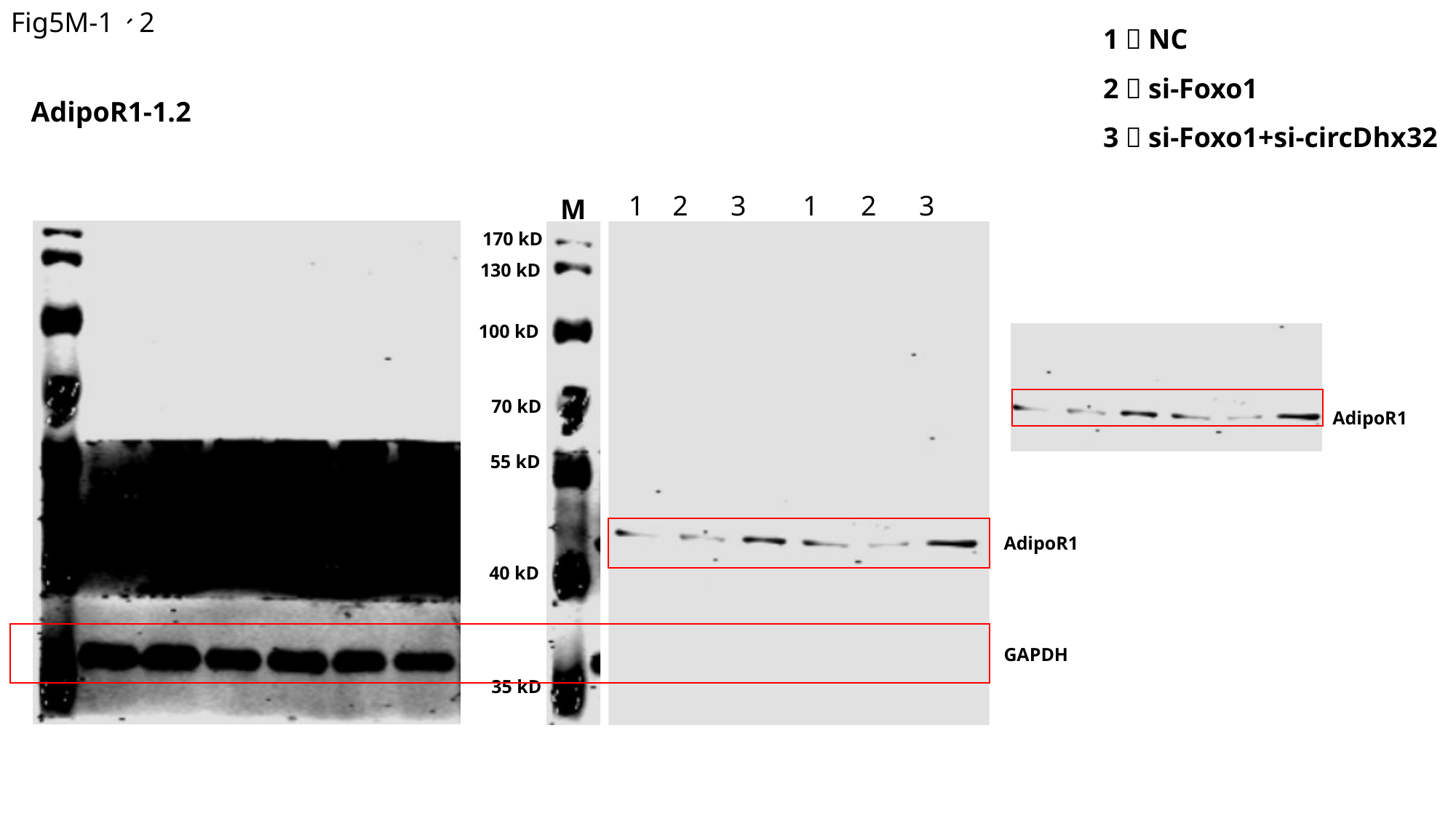

Fig5M-1、2
1：NC
2：si-Foxo1
3：si-Foxo1+si-circDhx32
AdipoR1-1.2
 1 2 3 1 2 3
M
170 kD
130 kD
100 kD
70 kD
AdipoR1
55 kD
AdipoR1
40 kD
GAPDH
35 kD

## Slide 18
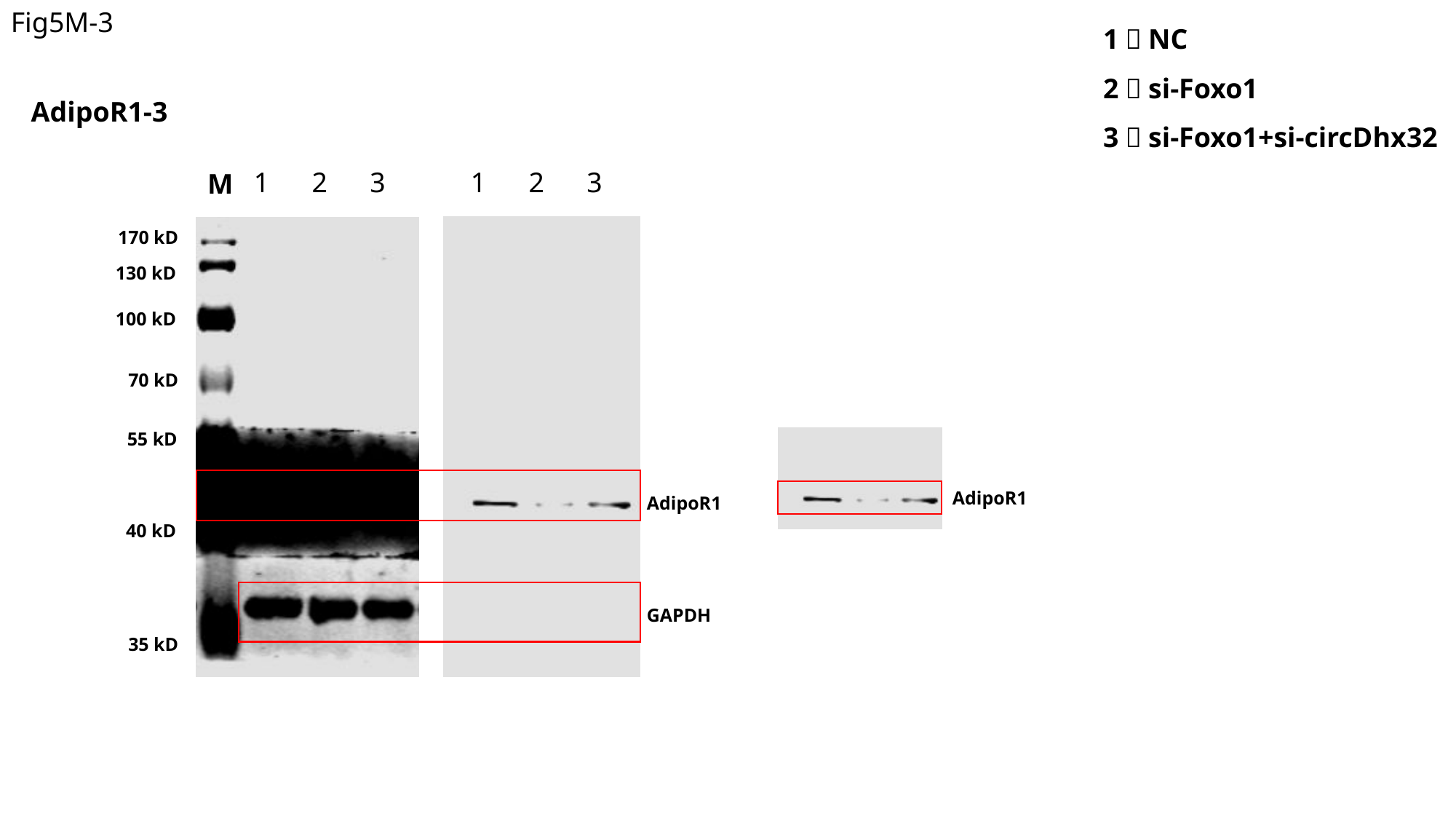

Fig5M-3
1：NC
2：si-Foxo1
3：si-Foxo1+si-circDhx32
AdipoR1-3
 1 2 3
 1 2 3
M
170 kD
130 kD
100 kD
70 kD
55 kD
AdipoR1
AdipoR1
40 kD
GAPDH
35 kD

## Slide 19
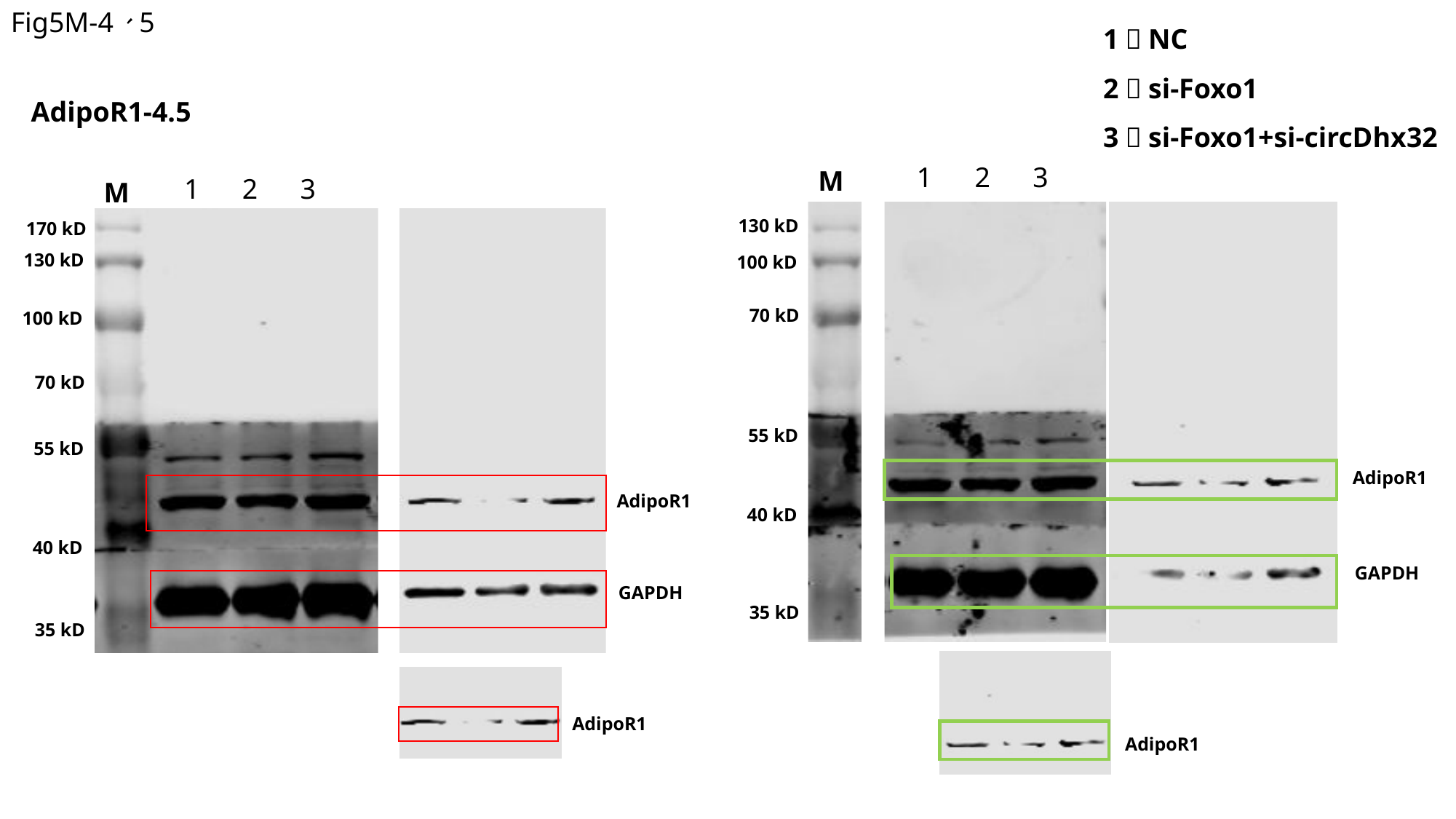

Fig5M-4、5
1：NC
2：si-Foxo1
3：si-Foxo1+si-circDhx32
AdipoR1-4.5
 1 2 3
M
 1 2 3
M
170 kD
130 kD
100 kD
70 kD
55 kD
AdipoR1
40 kD
GAPDH
35 kD
130 kD
100 kD
70 kD
55 kD
AdipoR1
40 kD
GAPDH
35 kD
AdipoR1
AdipoR1

## Slide 20
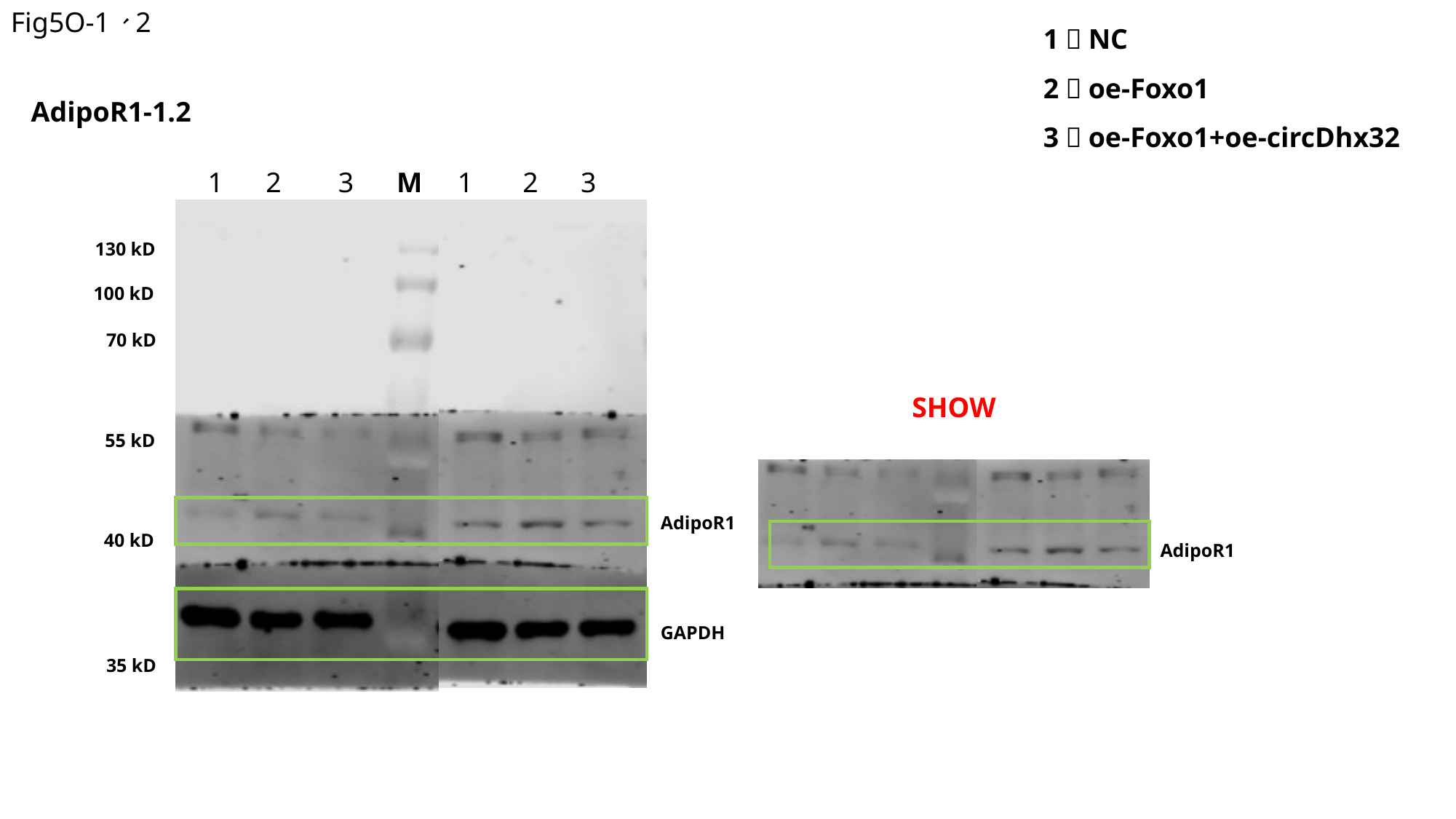

Fig5O-1、2
1：NC
2：oe-Foxo1
3：oe-Foxo1+oe-circDhx32
AdipoR1-1.2
 1 2 3 M 1 2 3
130 kD
100 kD
70 kD
SHOW
55 kD
AdipoR1
40 kD
AdipoR1
GAPDH
35 kD

## Slide 21
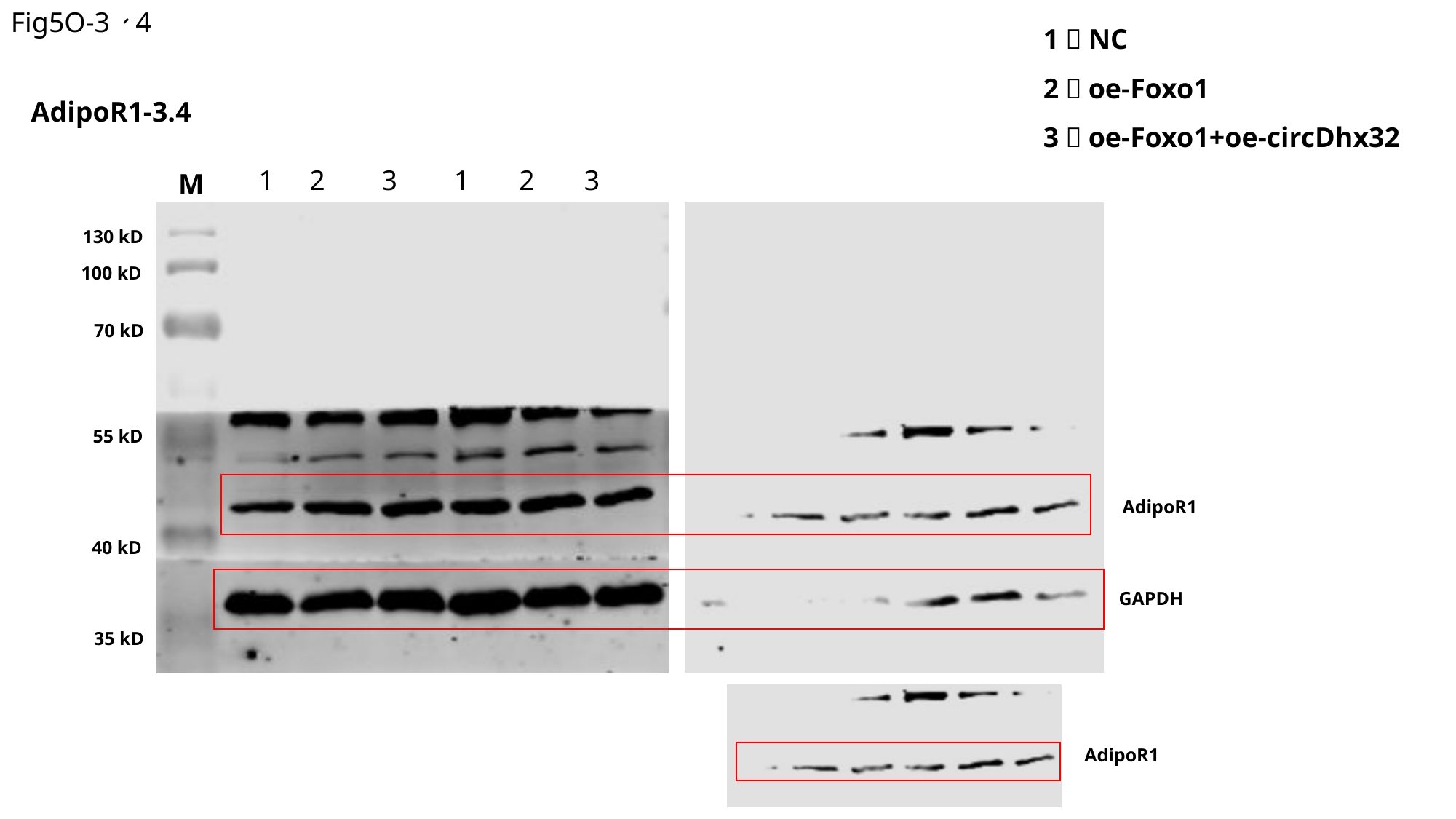

Fig5O-3、4
1：NC
2：oe-Foxo1
3：oe-Foxo1+oe-circDhx32
AdipoR1-3.4
 1 2 3 1 2 3
M
130 kD
100 kD
70 kD
55 kD
AdipoR1
40 kD
GAPDH
35 kD
AdipoR1

## Slide 22
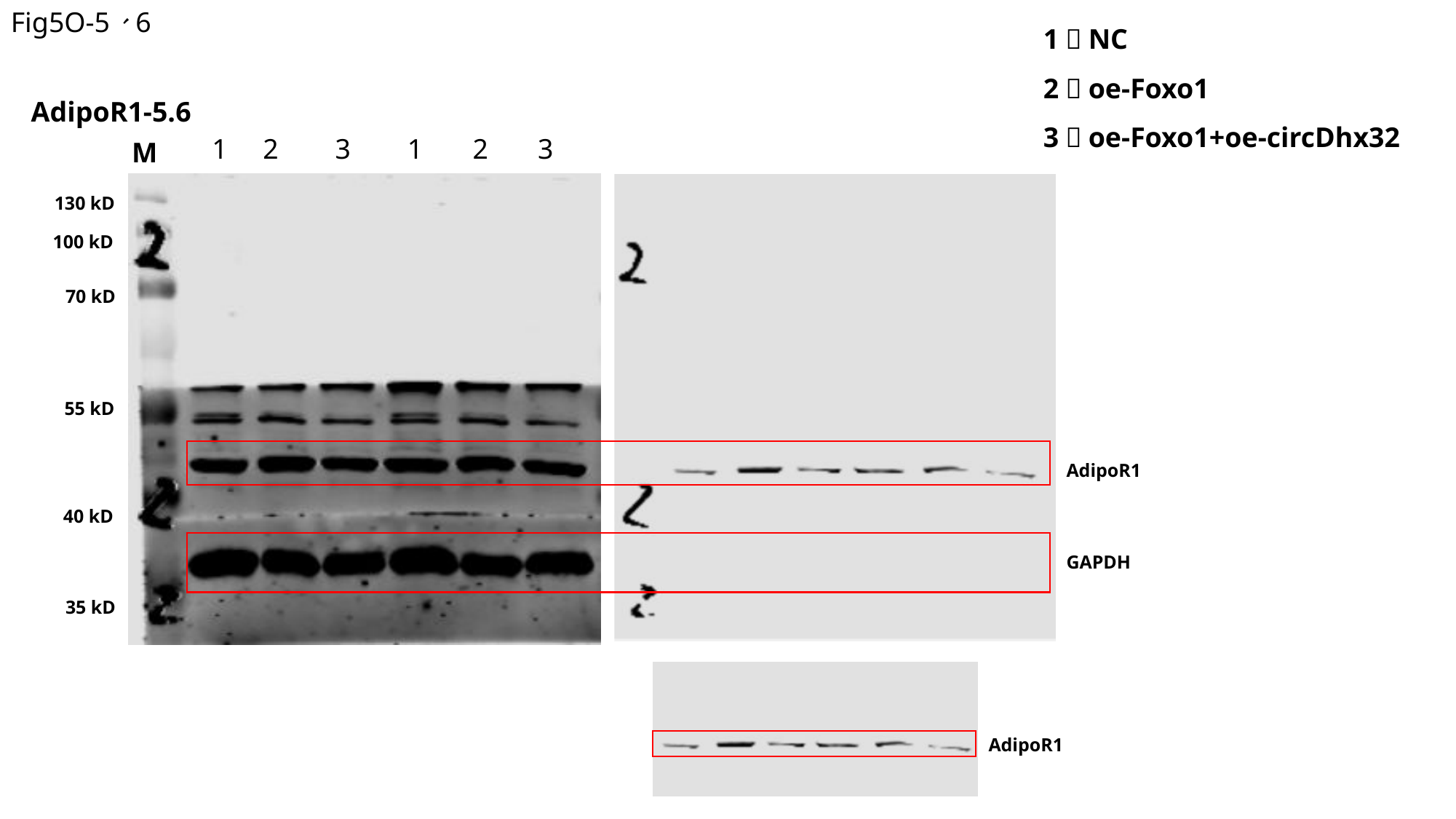

Fig5O-5、6
1：NC
2：oe-Foxo1
3：oe-Foxo1+oe-circDhx32
AdipoR1-5.6
 1 2 3 1 2 3
M
130 kD
100 kD
70 kD
55 kD
AdipoR1
40 kD
GAPDH
35 kD
AdipoR1

## Slide 23
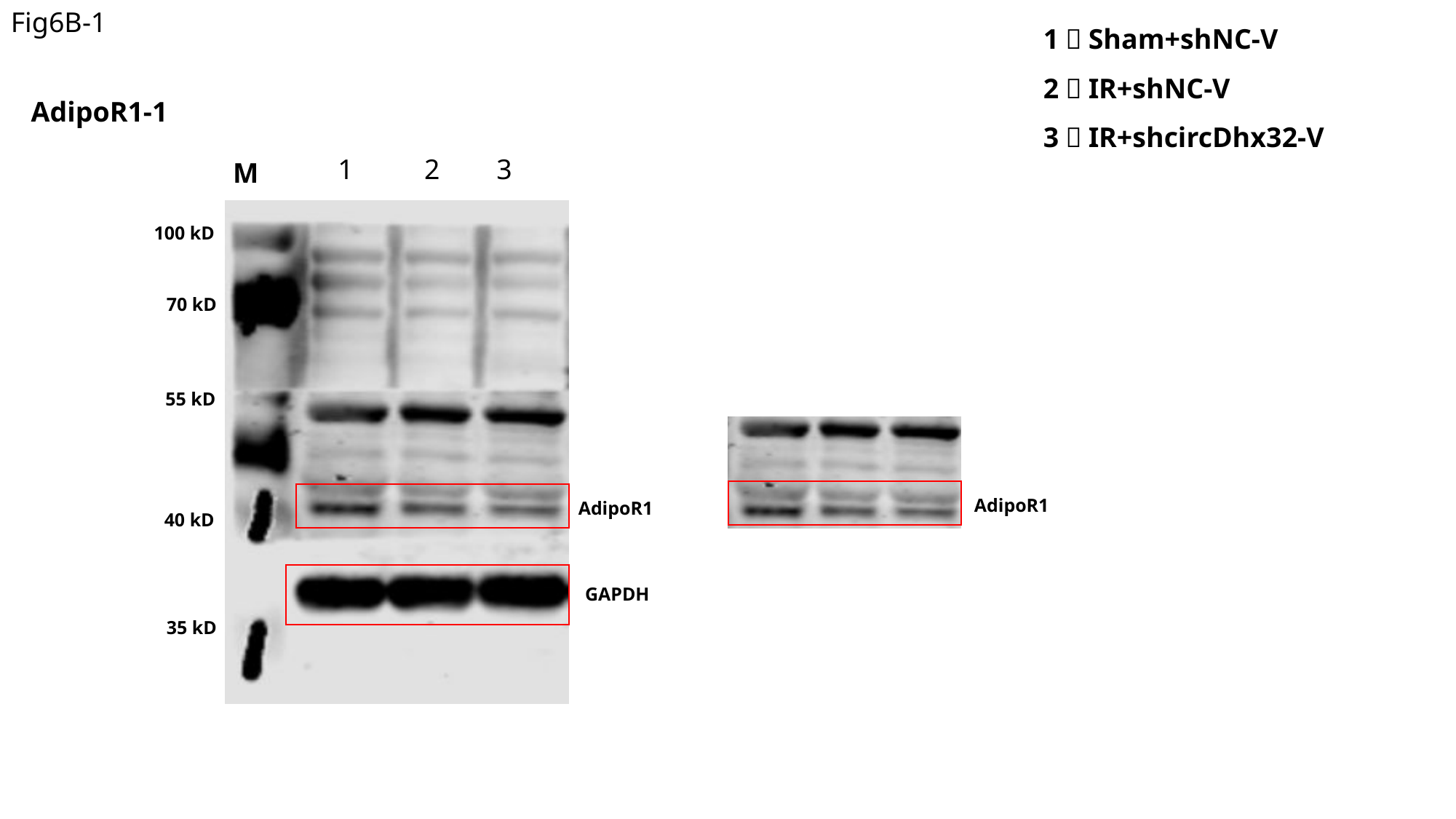

Fig6B-1
1：Sham+shNC-V
2：IR+shNC-V
3：IR+shcircDhx32-V
AdipoR1-1
 1 2 3
M
100 kD
70 kD
55 kD
AdipoR1
AdipoR1
40 kD
GAPDH
35 kD

## Slide 24
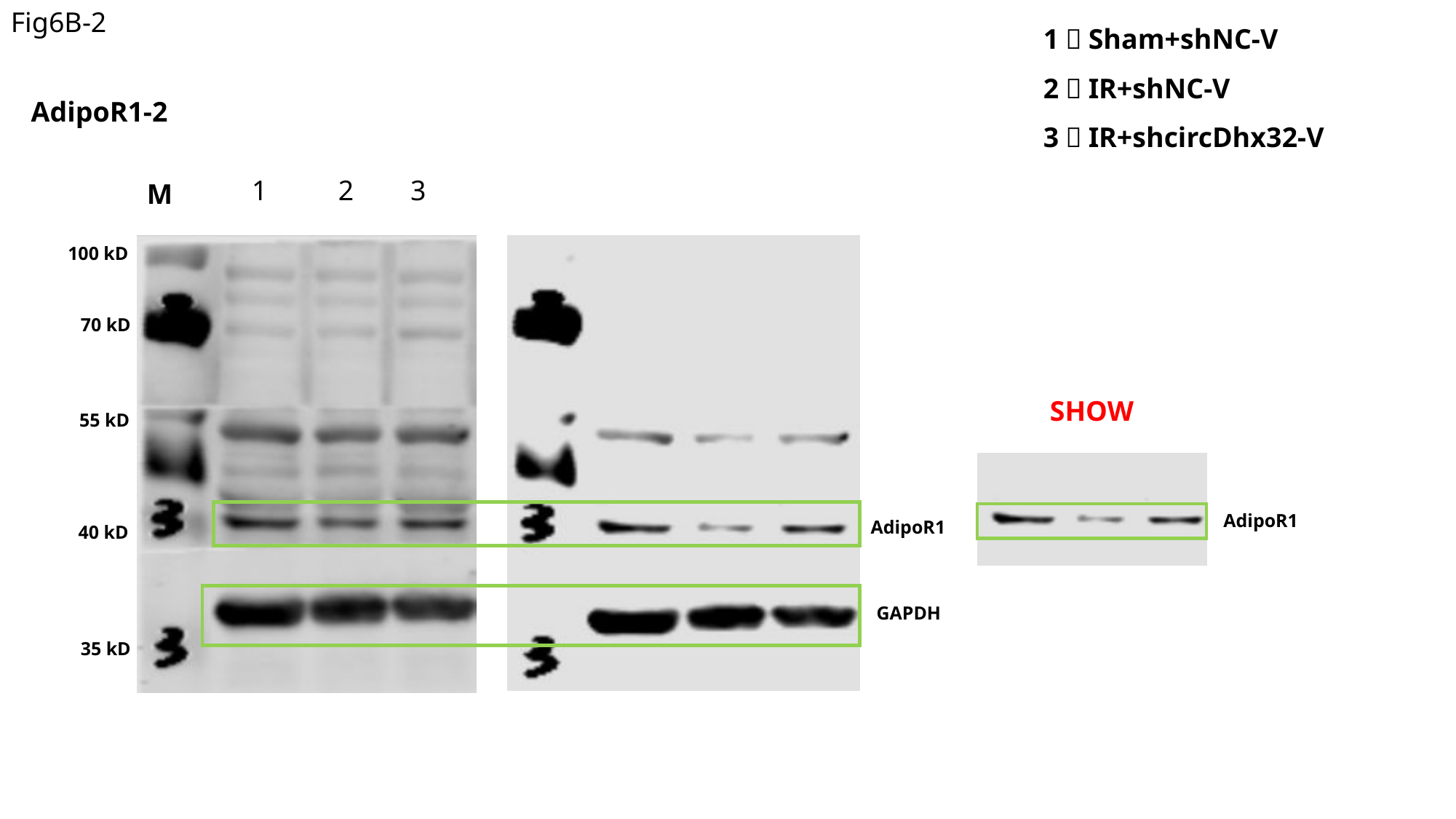

Fig6B-2
1：Sham+shNC-V
2：IR+shNC-V
3：IR+shcircDhx32-V
AdipoR1-2
 1 2 3
M
100 kD
70 kD
SHOW
55 kD
AdipoR1
AdipoR1
40 kD
GAPDH
35 kD

## Slide 25
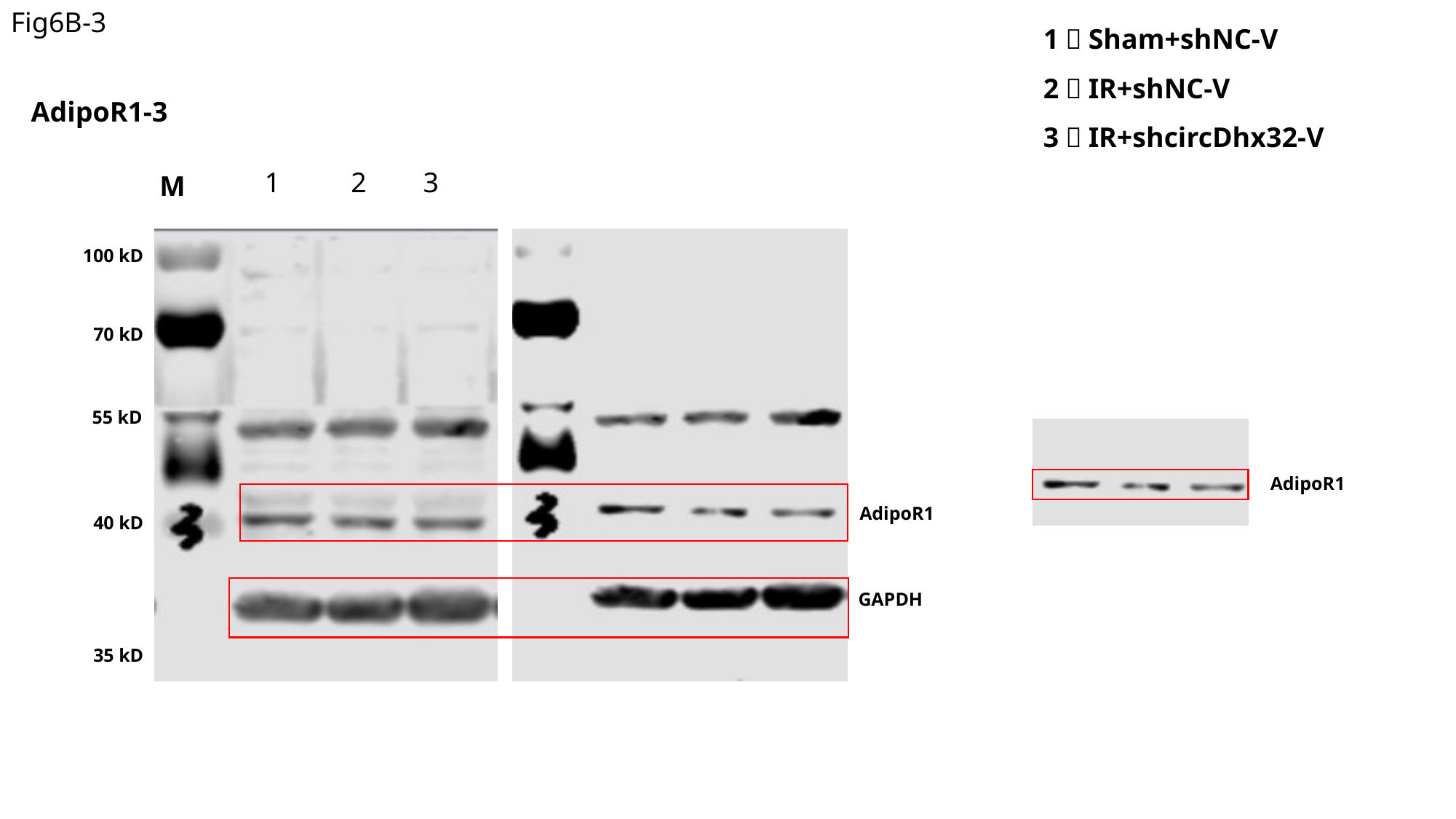

Fig6B-3
1：Sham+shNC-V
2：IR+shNC-V
3：IR+shcircDhx32-V
AdipoR1-3
 1 2 3
M
100 kD
70 kD
55 kD
AdipoR1
AdipoR1
40 kD
GAPDH
35 kD

## Slide 26
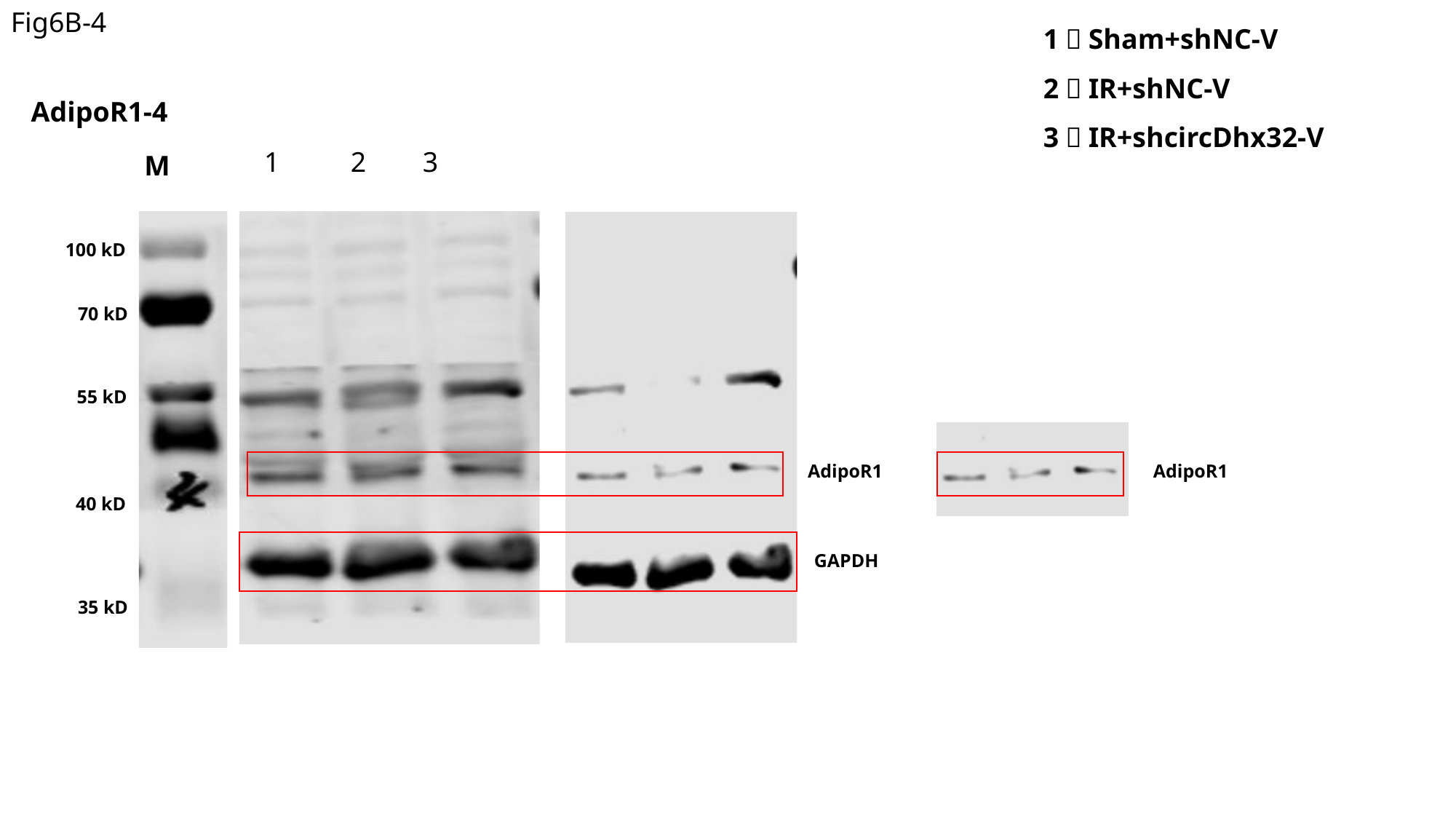

Fig6B-4
1：Sham+shNC-V
2：IR+shNC-V
3：IR+shcircDhx32-V
AdipoR1-4
 1 2 3
M
100 kD
70 kD
55 kD
AdipoR1
AdipoR1
40 kD
GAPDH
35 kD

## Slide 27
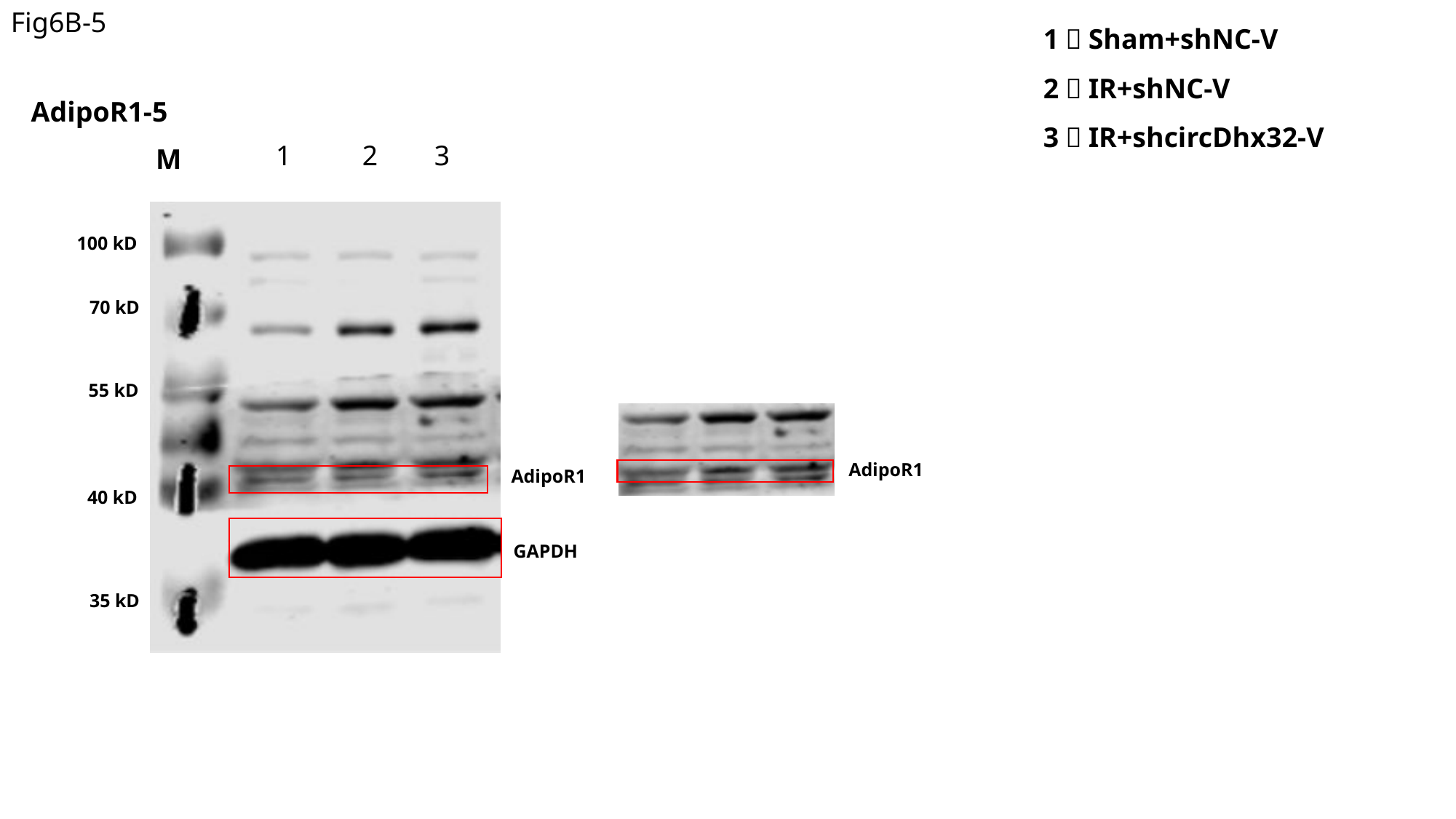

Fig6B-5
1：Sham+shNC-V
2：IR+shNC-V
3：IR+shcircDhx32-V
AdipoR1-5
 1 2 3
M
100 kD
70 kD
55 kD
AdipoR1
AdipoR1
40 kD
GAPDH
35 kD

## Slide 28
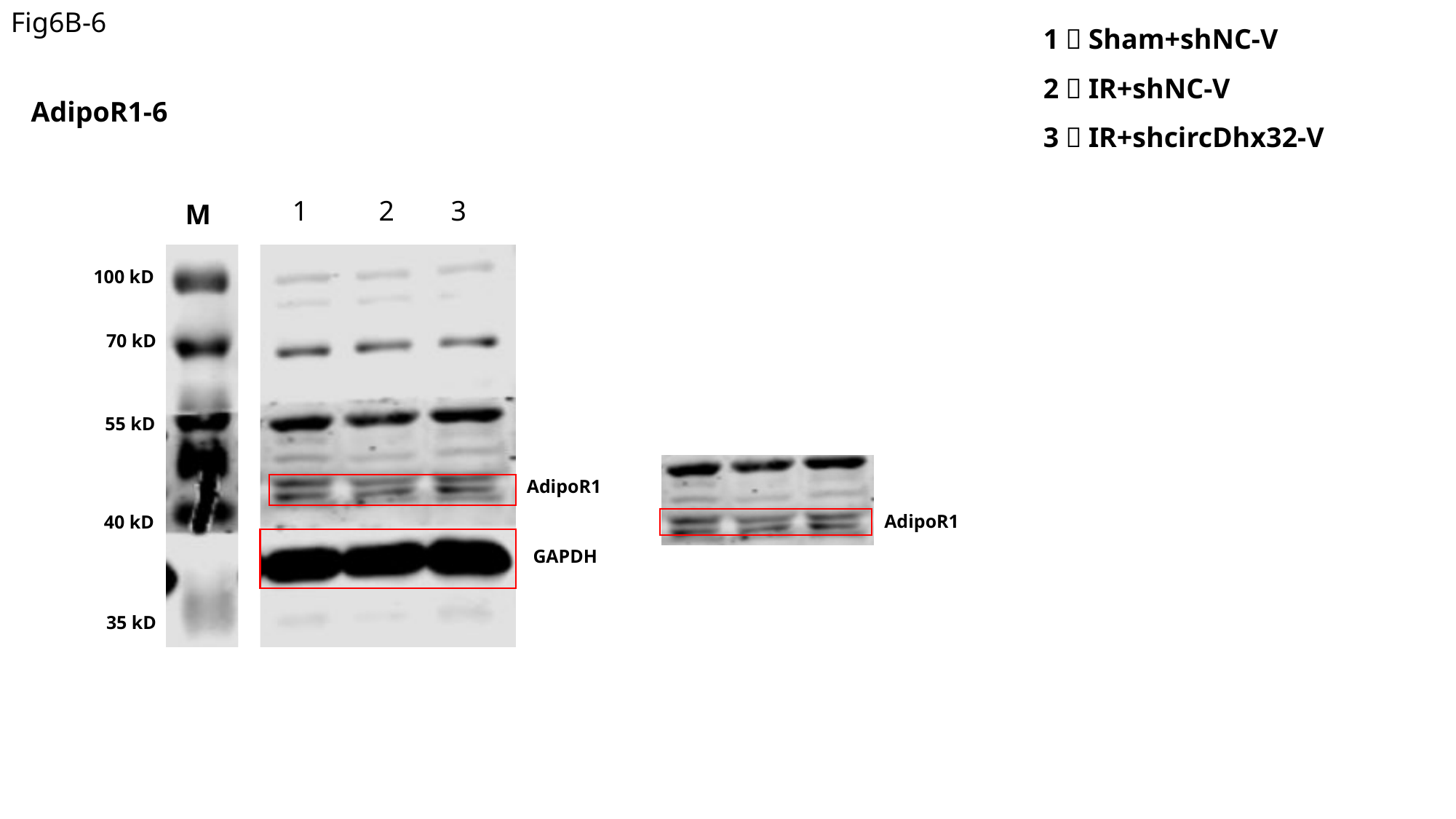

Fig6B-6
1：Sham+shNC-V
2：IR+shNC-V
3：IR+shcircDhx32-V
AdipoR1-6
 1 2 3
M
100 kD
70 kD
55 kD
AdipoR1
AdipoR1
40 kD
GAPDH
35 kD

## Slide 29
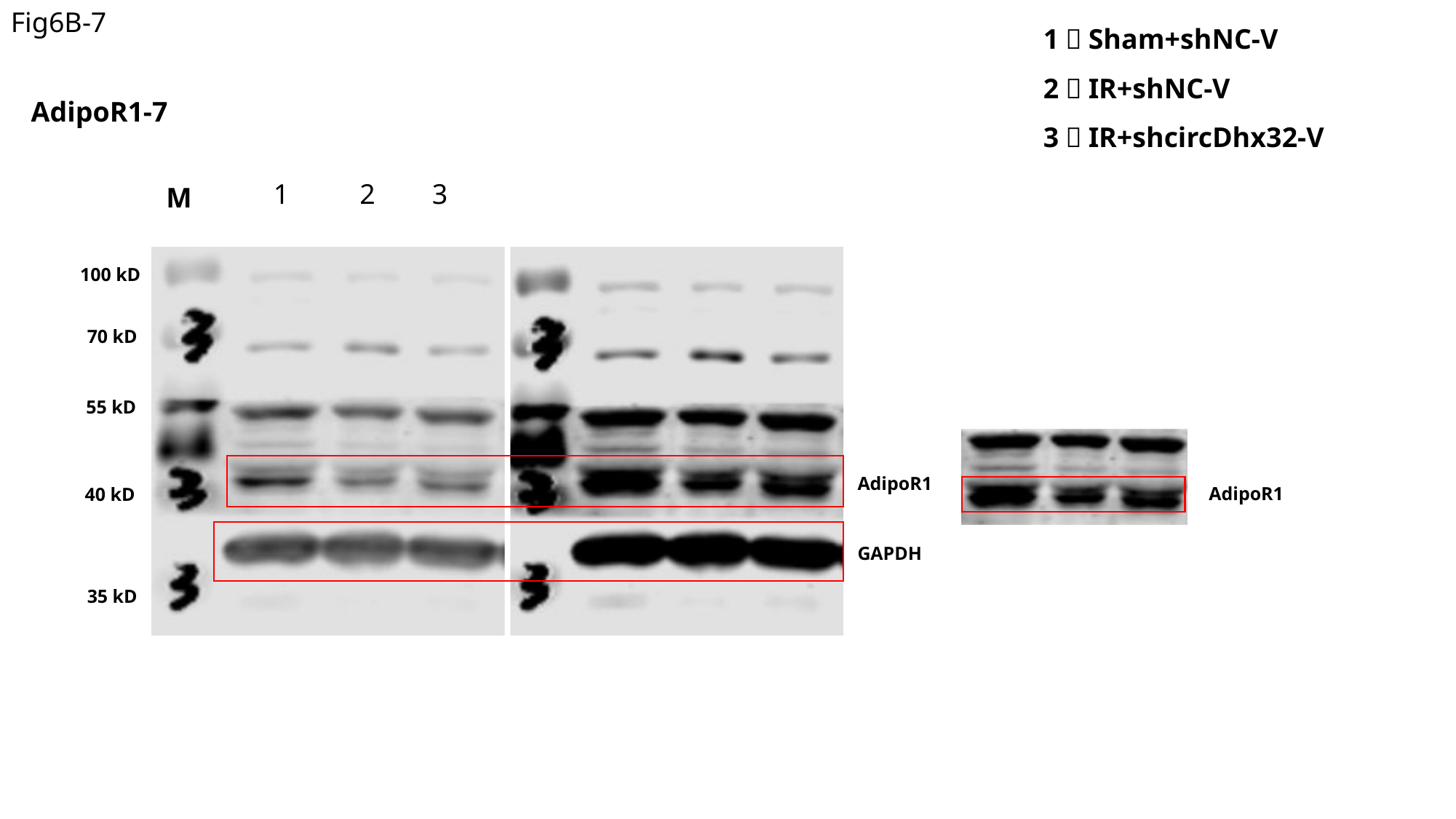

Fig6B-7
1：Sham+shNC-V
2：IR+shNC-V
3：IR+shcircDhx32-V
AdipoR1-7
 1 2 3
M
100 kD
70 kD
55 kD
AdipoR1
AdipoR1
40 kD
GAPDH
35 kD

## Slide 30
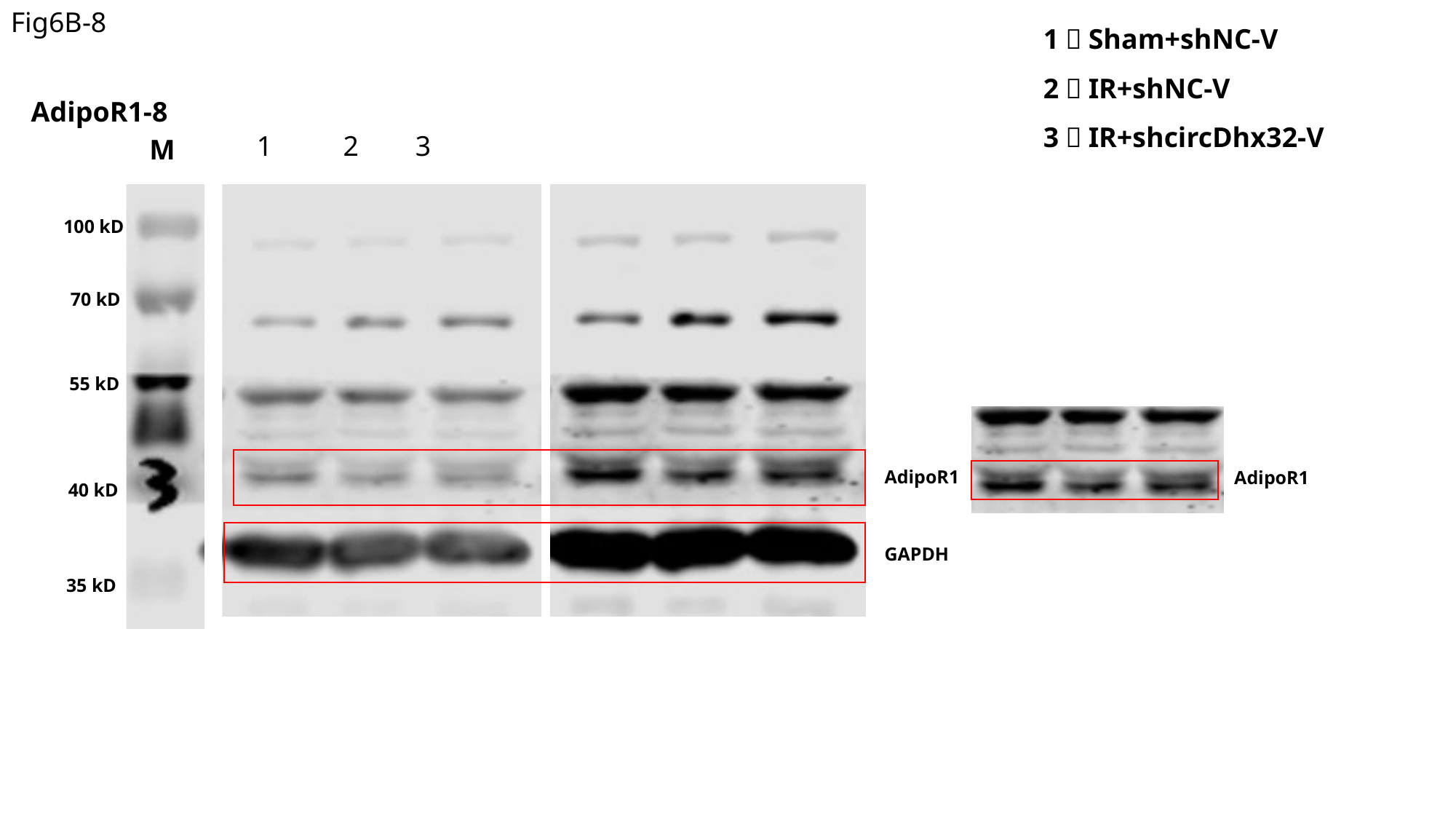

Fig6B-8
1：Sham+shNC-V
2：IR+shNC-V
3：IR+shcircDhx32-V
AdipoR1-8
 1 2 3
M
100 kD
70 kD
55 kD
AdipoR1
AdipoR1
40 kD
GAPDH
35 kD

## Slide 31
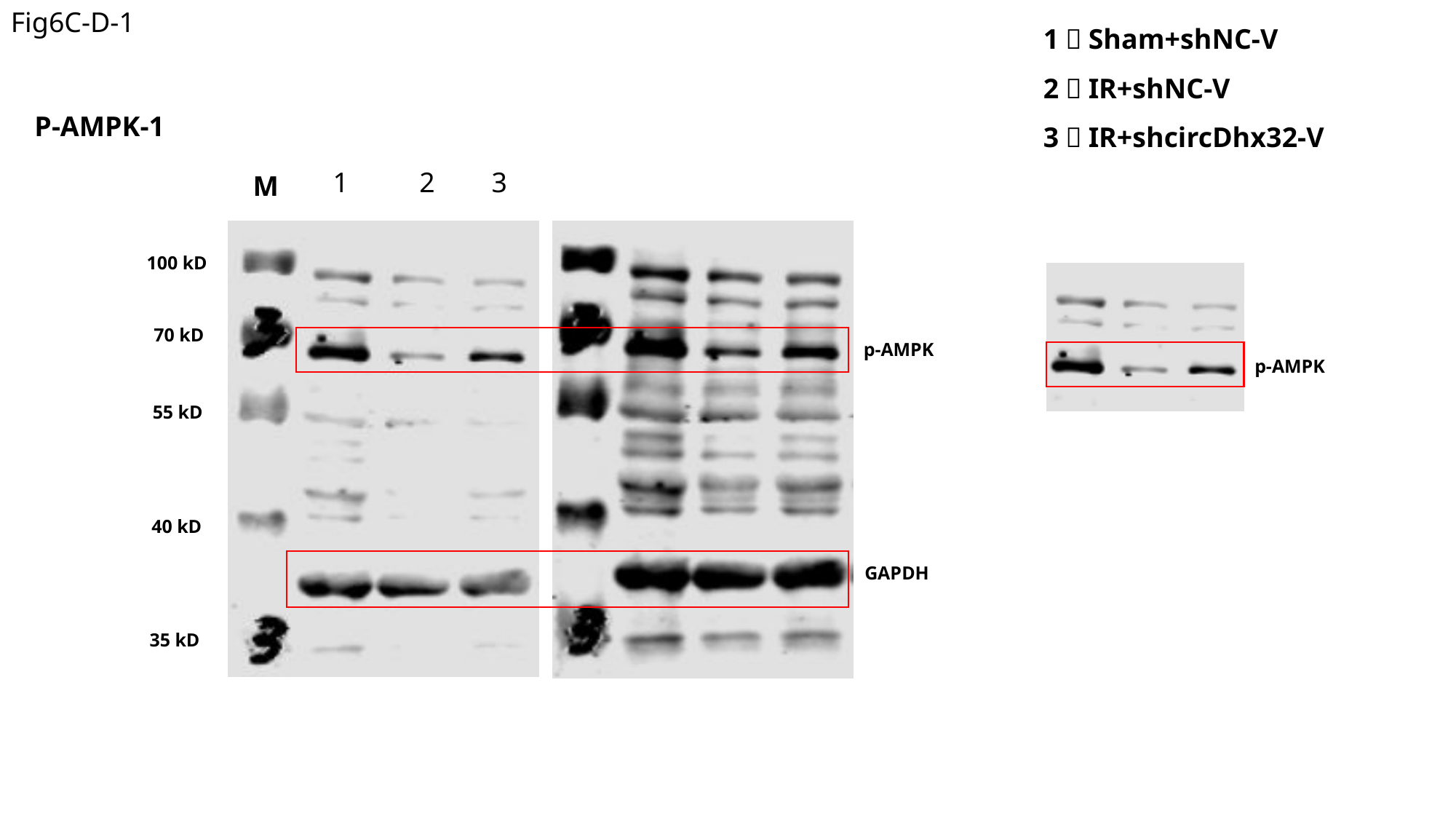

Fig6C-D-1
1：Sham+shNC-V
2：IR+shNC-V
3：IR+shcircDhx32-V
P-AMPK-1
 1 2 3
M
100 kD
70 kD
p-AMPK
p-AMPK
55 kD
40 kD
GAPDH
35 kD

## Slide 32
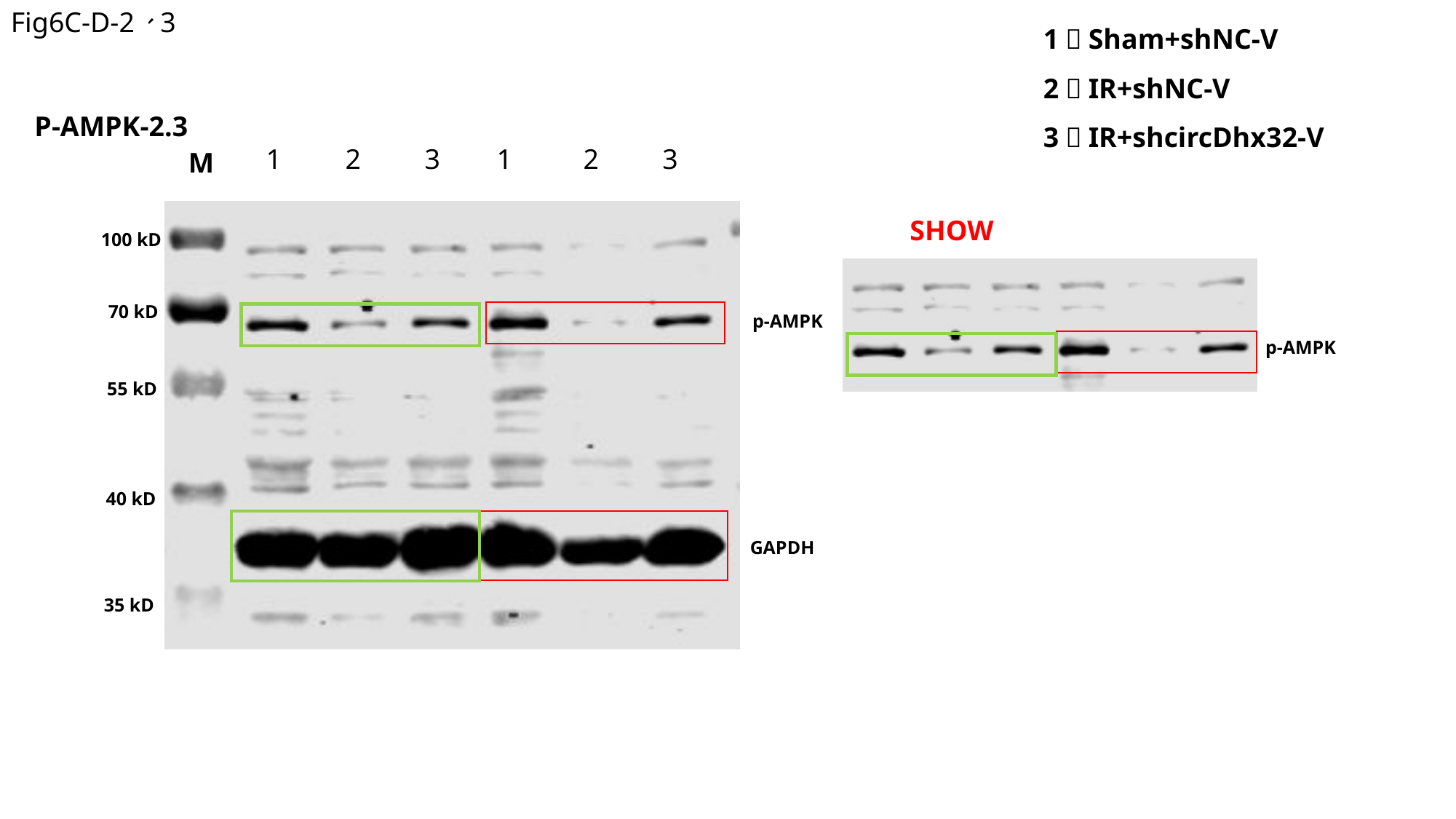

Fig6C-D-2、3
1：Sham+shNC-V
2：IR+shNC-V
3：IR+shcircDhx32-V
P-AMPK-2.3
 1 2 3 1 2 3
M
SHOW
100 kD
70 kD
p-AMPK
p-AMPK
55 kD
40 kD
GAPDH
35 kD

## Slide 33
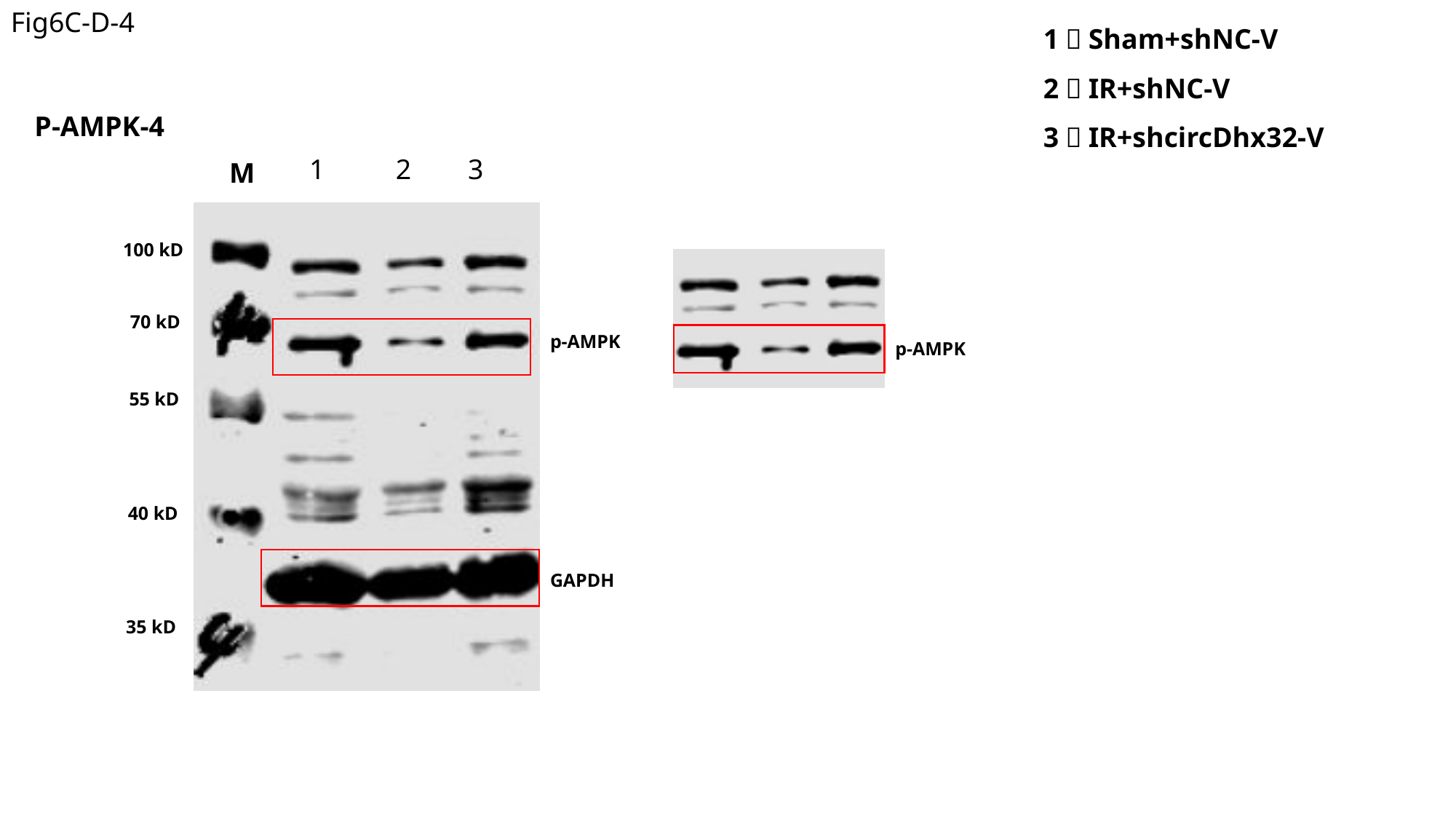

Fig6C-D-4
1：Sham+shNC-V
2：IR+shNC-V
3：IR+shcircDhx32-V
P-AMPK-4
 1 2 3
M
100 kD
70 kD
p-AMPK
p-AMPK
55 kD
40 kD
GAPDH
35 kD

## Slide 34
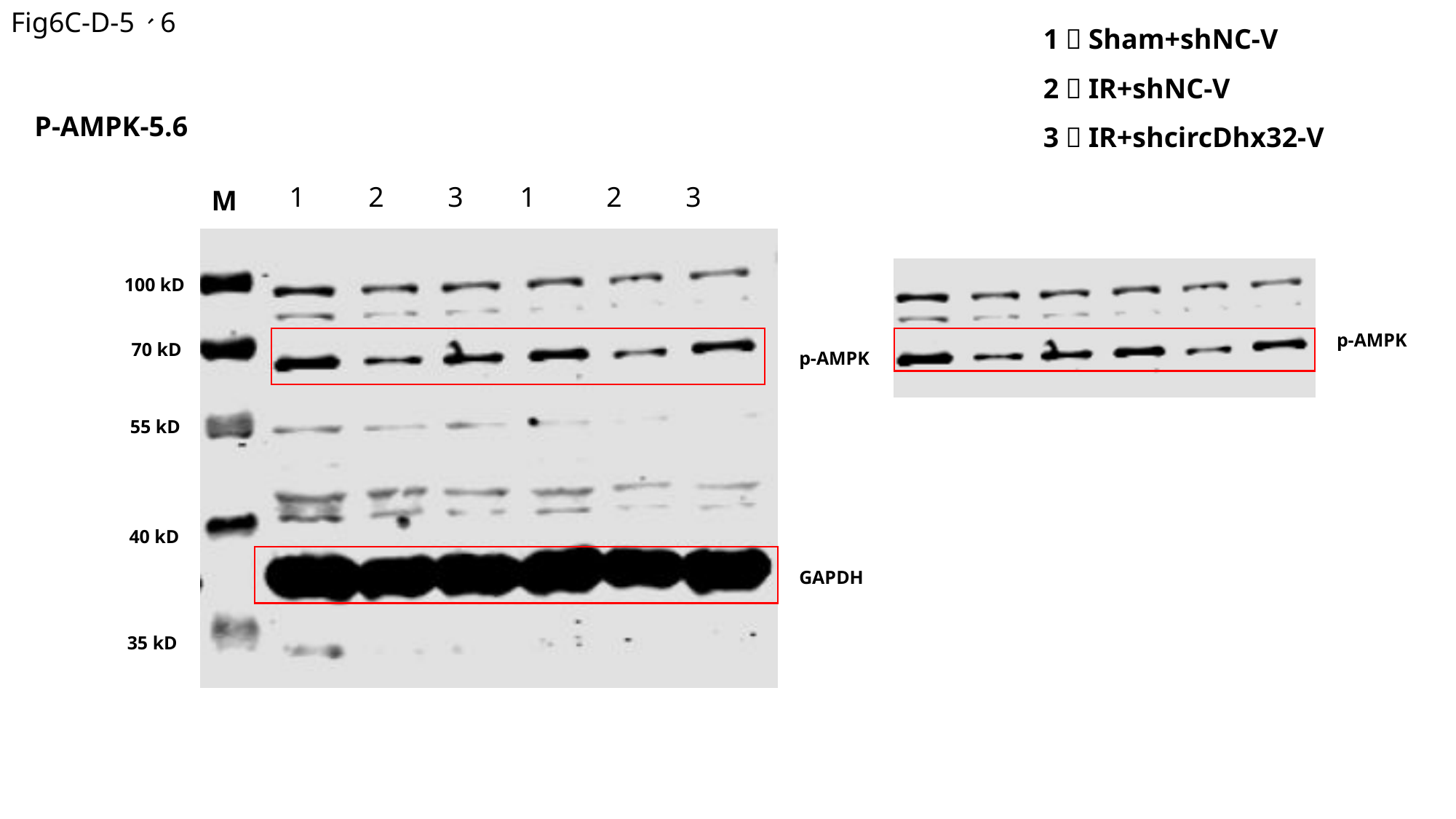

Fig6C-D-5、6
1：Sham+shNC-V
2：IR+shNC-V
3：IR+shcircDhx32-V
P-AMPK-5.6
 1 2 3 1 2 3
M
100 kD
p-AMPK
70 kD
p-AMPK
55 kD
40 kD
GAPDH
35 kD

## Slide 35
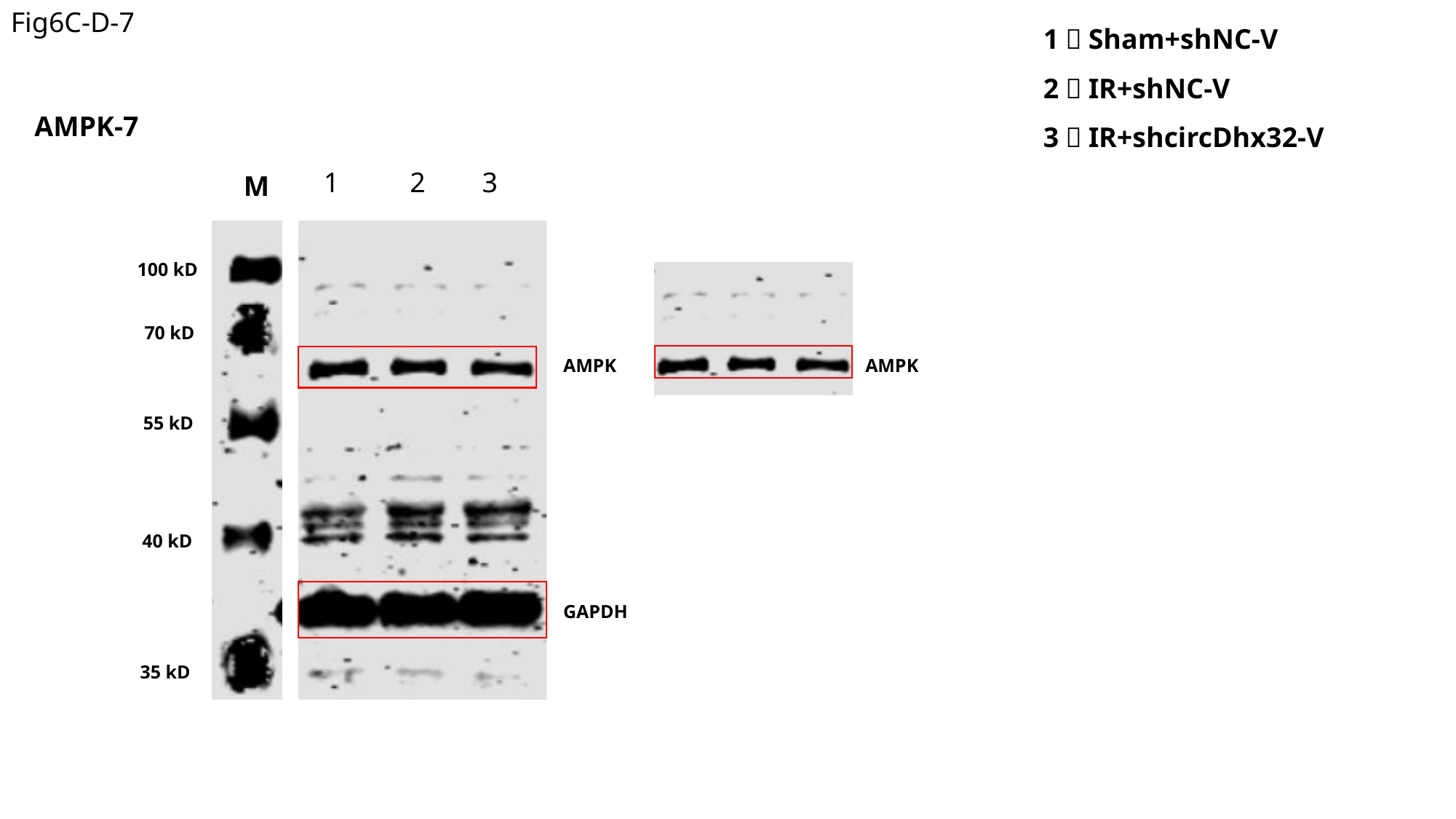

Fig6C-D-7
1：Sham+shNC-V
2：IR+shNC-V
3：IR+shcircDhx32-V
AMPK-7
 1 2 3
M
100 kD
70 kD
AMPK
AMPK
55 kD
40 kD
GAPDH
35 kD

## Slide 36
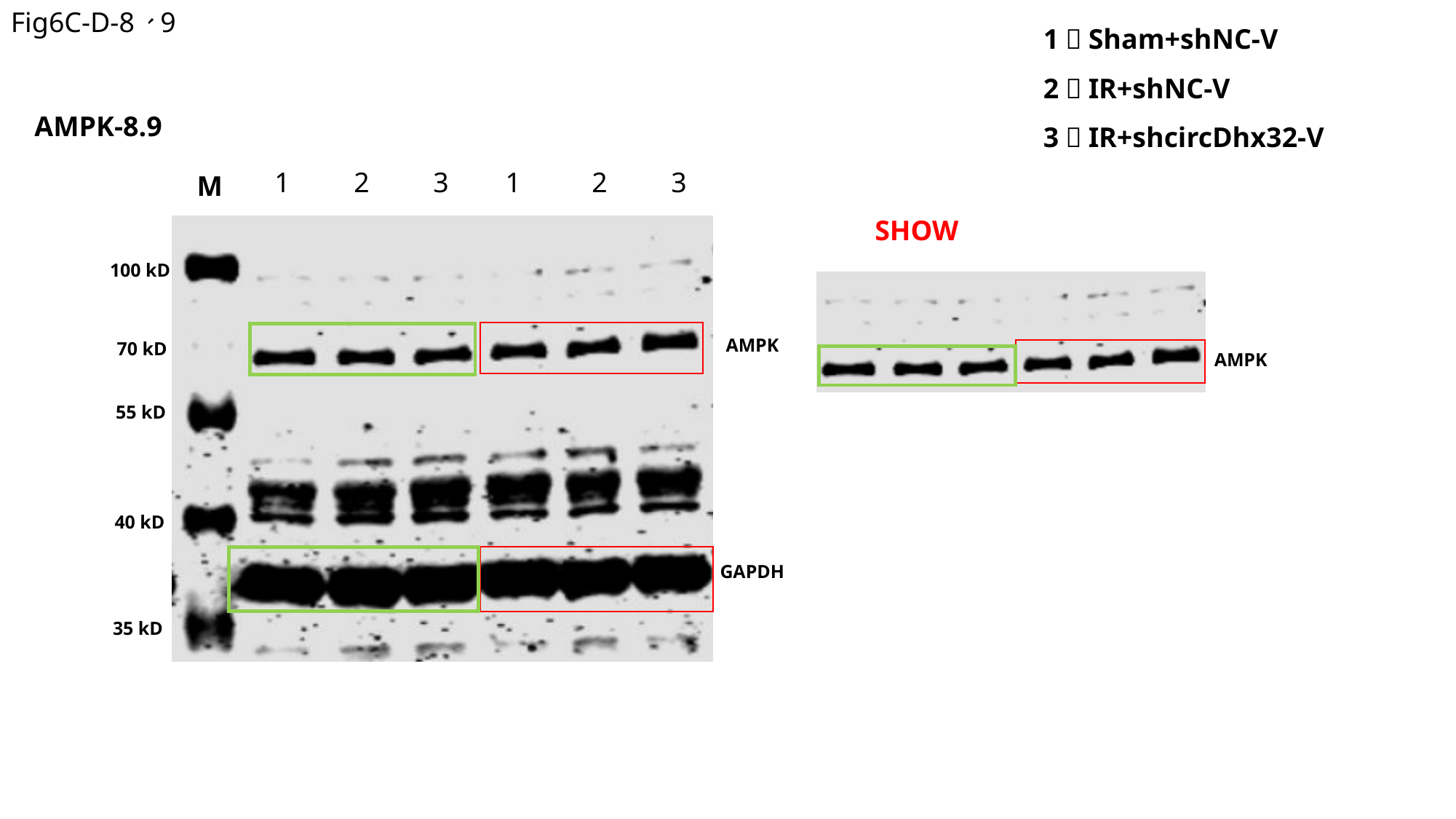

Fig6C-D-8、9
1：Sham+shNC-V
2：IR+shNC-V
3：IR+shcircDhx32-V
AMPK-8.9
 1 2 3 1 2 3
M
SHOW
100 kD
AMPK
70 kD
AMPK
55 kD
40 kD
GAPDH
35 kD

## Slide 37
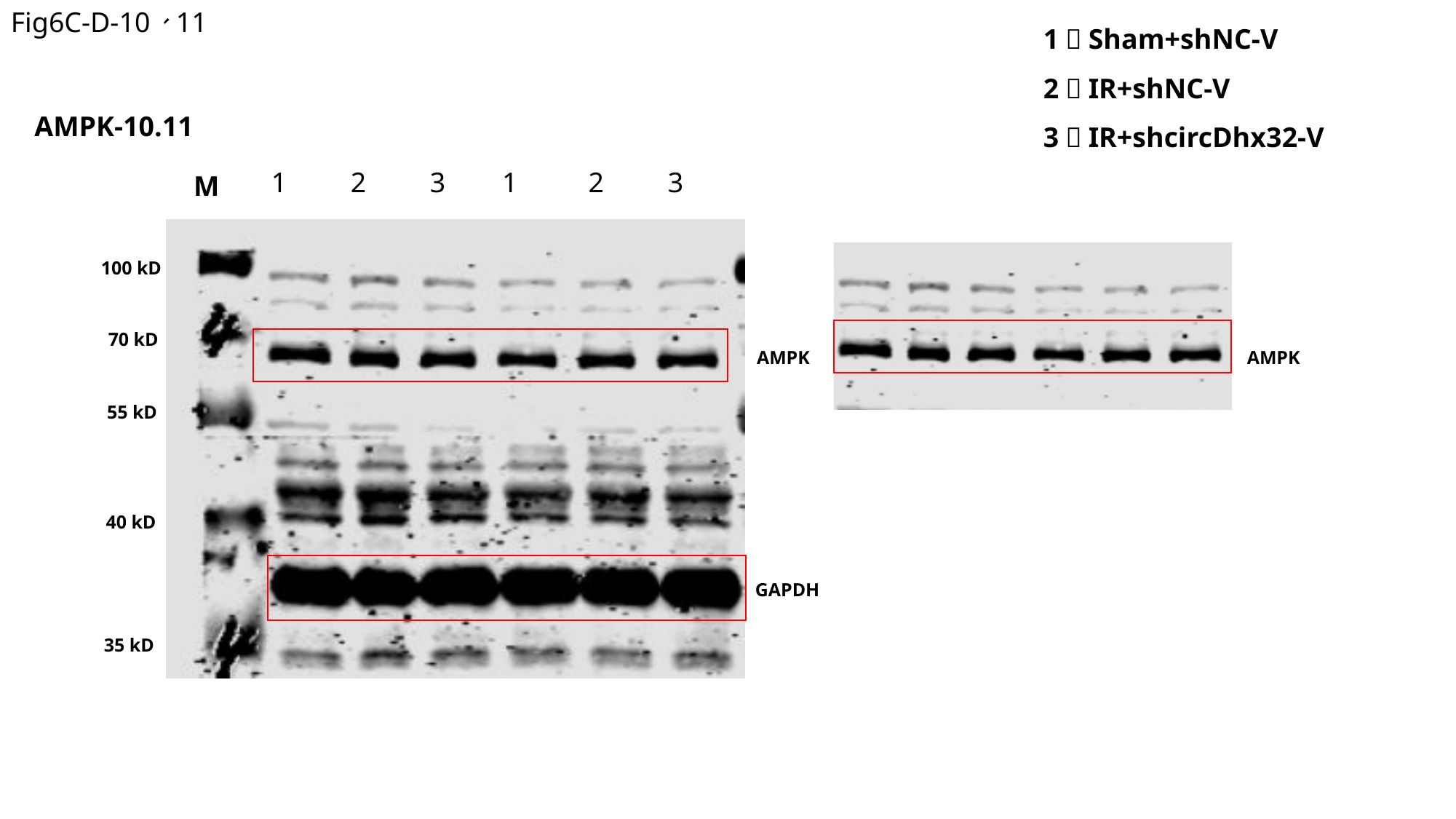

Fig6C-D-10、11
1：Sham+shNC-V
2：IR+shNC-V
3：IR+shcircDhx32-V
AMPK-10.11
 1 2 3 1 2 3
M
100 kD
70 kD
AMPK
AMPK
55 kD
40 kD
GAPDH
35 kD

## Slide 38
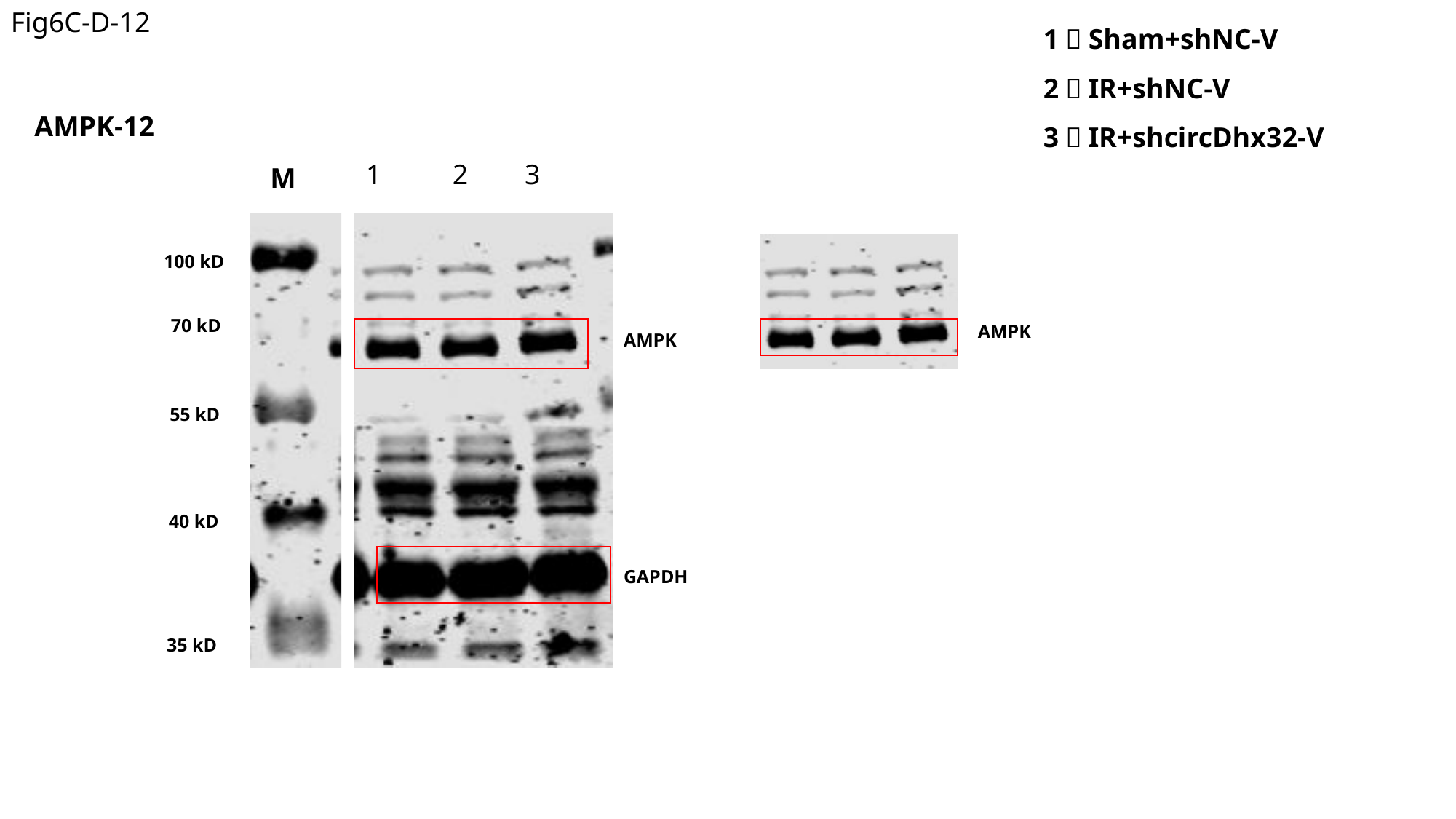

Fig6C-D-12
1：Sham+shNC-V
2：IR+shNC-V
3：IR+shcircDhx32-V
AMPK-12
 1 2 3
M
100 kD
70 kD
AMPK
AMPK
55 kD
40 kD
GAPDH
35 kD

## Slide 39
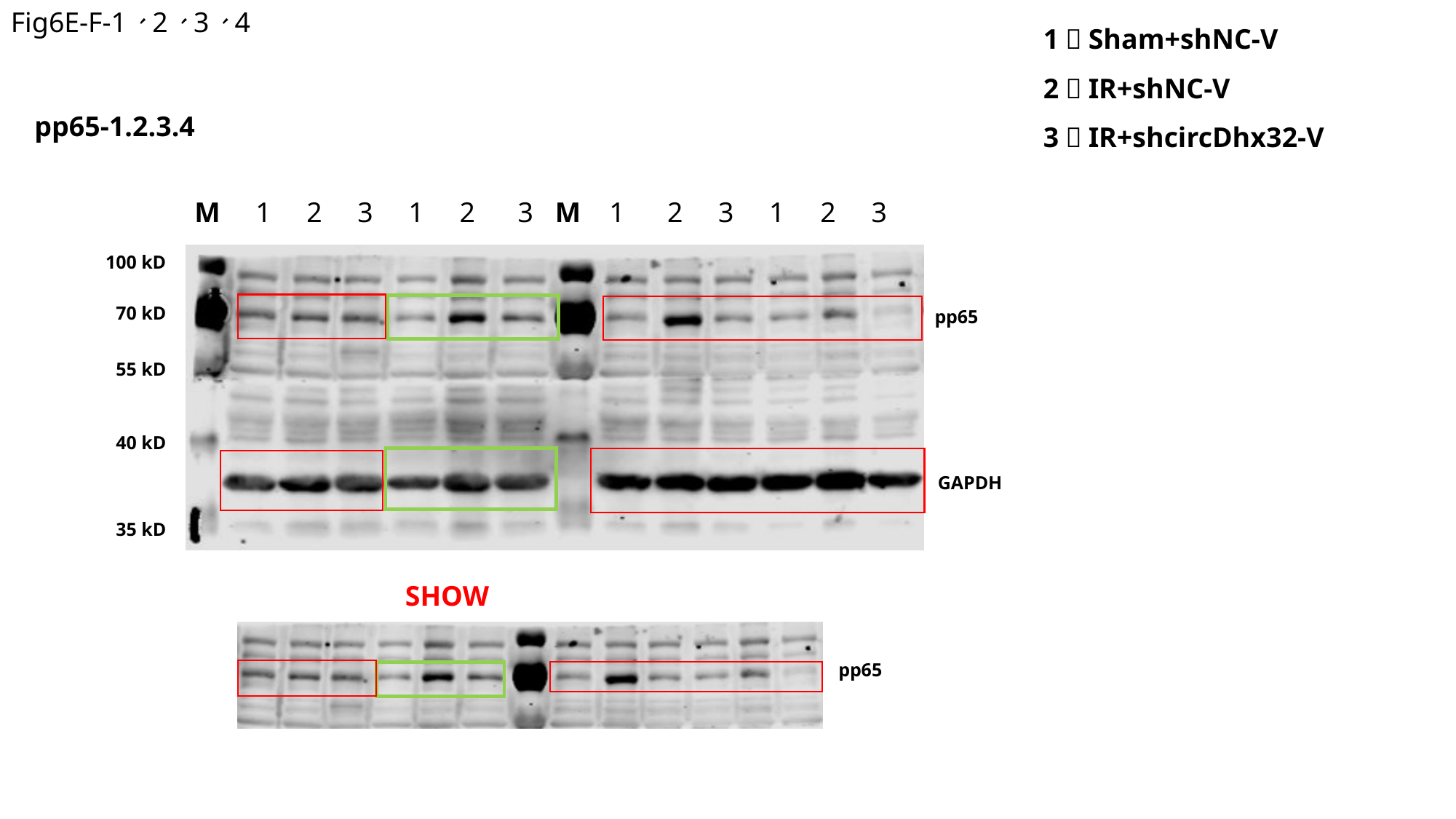

Fig6E-F-1、2、3、4
1：Sham+shNC-V
2：IR+shNC-V
3：IR+shcircDhx32-V
pp65-1.2.3.4
M
 1 2 3 1 2 3 M 1 2 3 1 2 3
100 kD
70 kD
pp65
55 kD
40 kD
GAPDH
35 kD
SHOW
pp65

## Slide 40
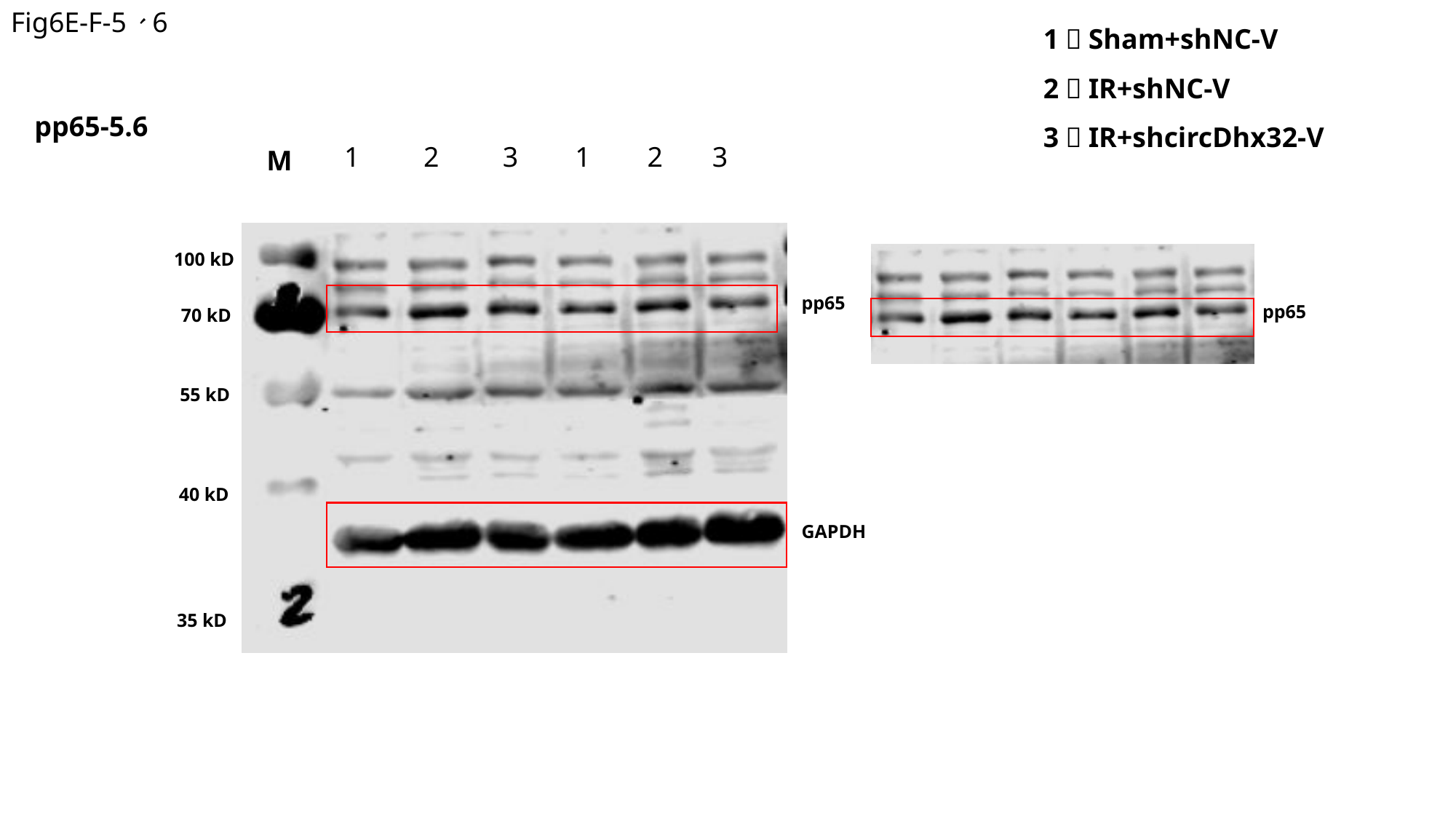

Fig6E-F-5、6
1：Sham+shNC-V
2：IR+shNC-V
3：IR+shcircDhx32-V
pp65-5.6
 1 2 3 1 2 3
M
100 kD
pp65
pp65
70 kD
55 kD
40 kD
GAPDH
35 kD

## Slide 41
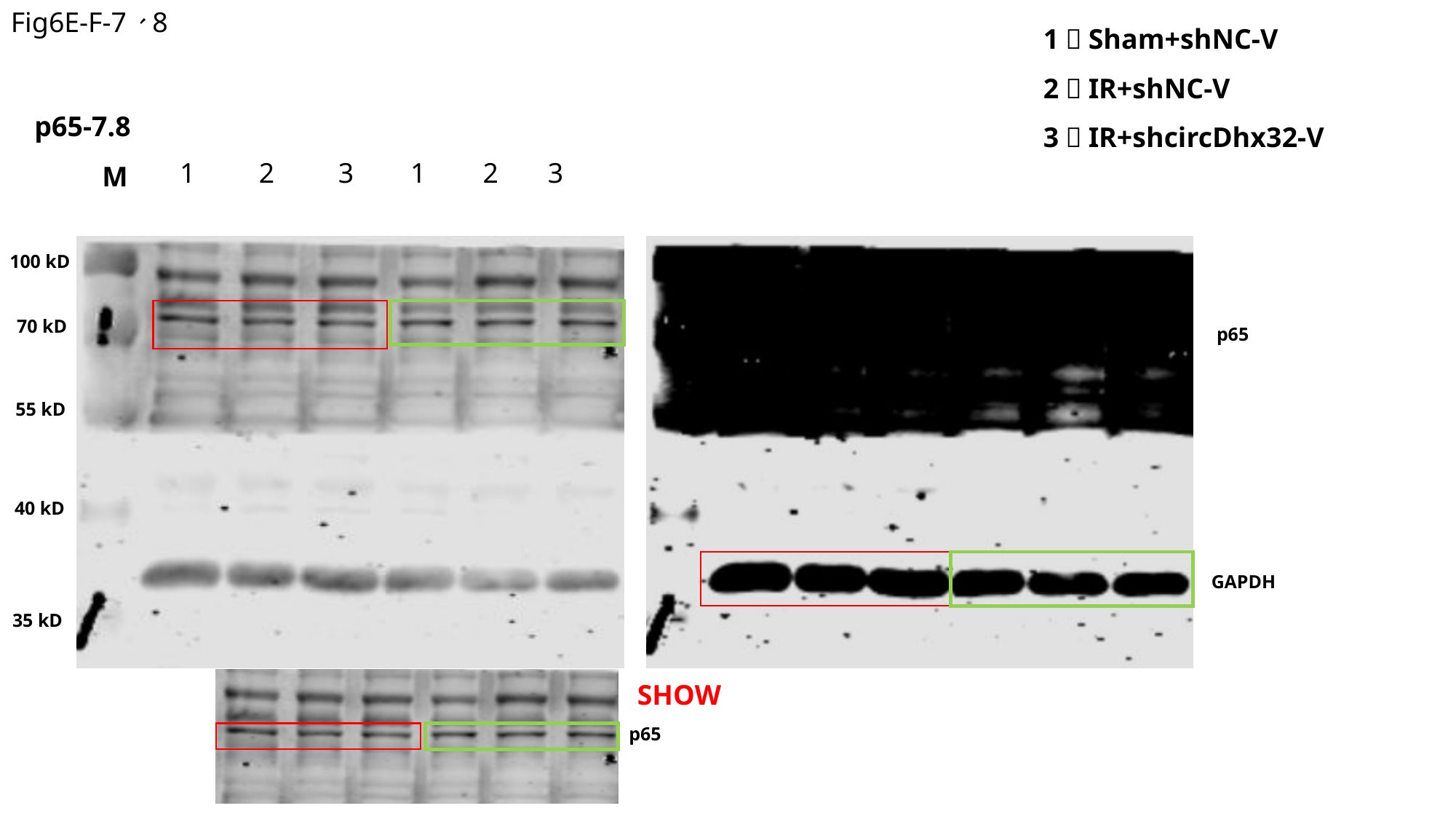

Fig6E-F-7、8
1：Sham+shNC-V
2：IR+shNC-V
3：IR+shcircDhx32-V
p65-7.8
 1 2 3 1 2 3
M
100 kD
70 kD
p65
55 kD
40 kD
GAPDH
35 kD
SHOW
p65

## Slide 42
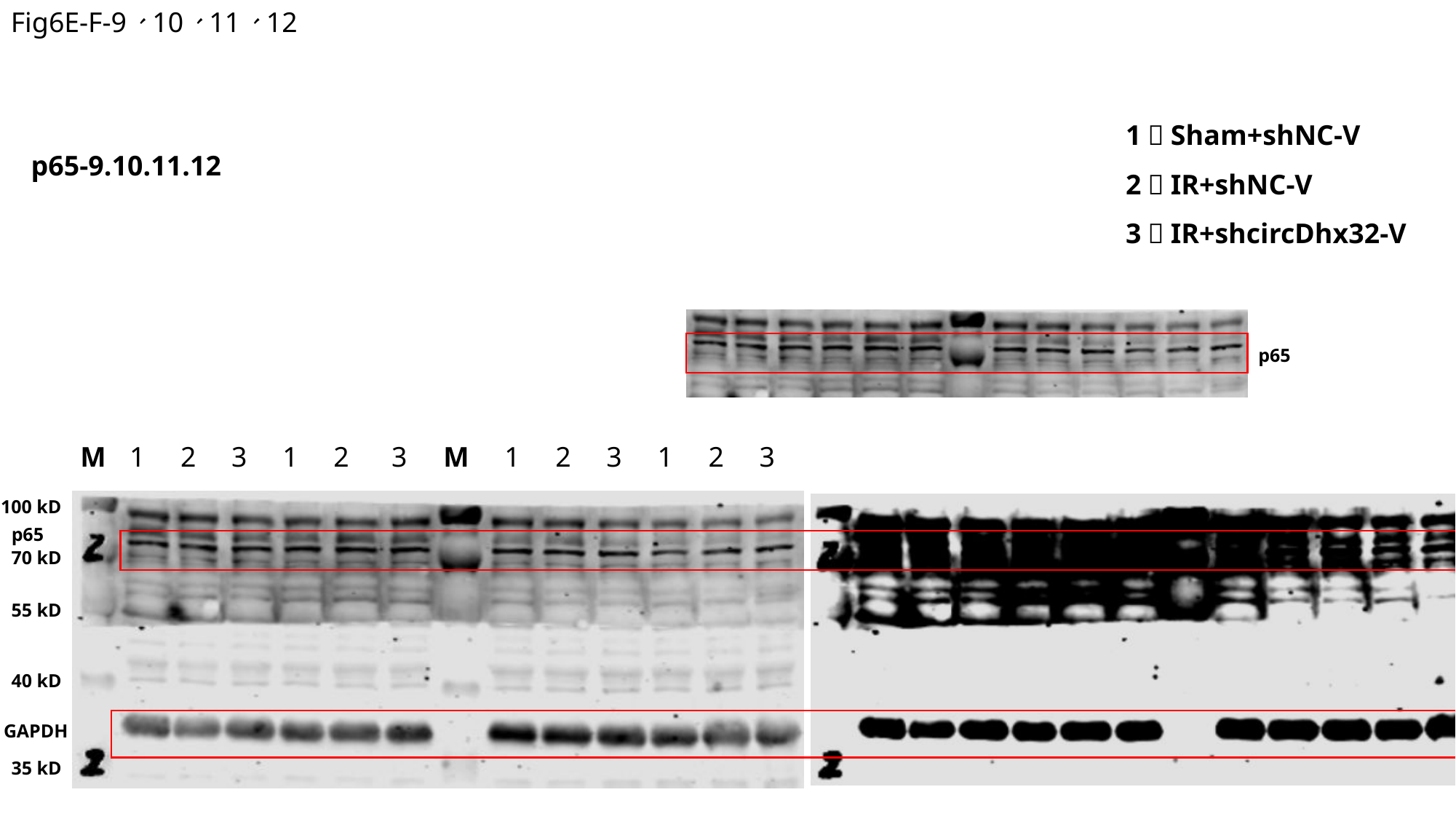

Fig6E-F-9、10、11、12
1：Sham+shNC-V
2：IR+shNC-V
3：IR+shcircDhx32-V
p65-9.10.11.12
p65
M
 1 2 3 1 2 3 M 1 2 3 1 2 3
100 kD
p65
70 kD
55 kD
40 kD
GAPDH
35 kD

## Slide 43
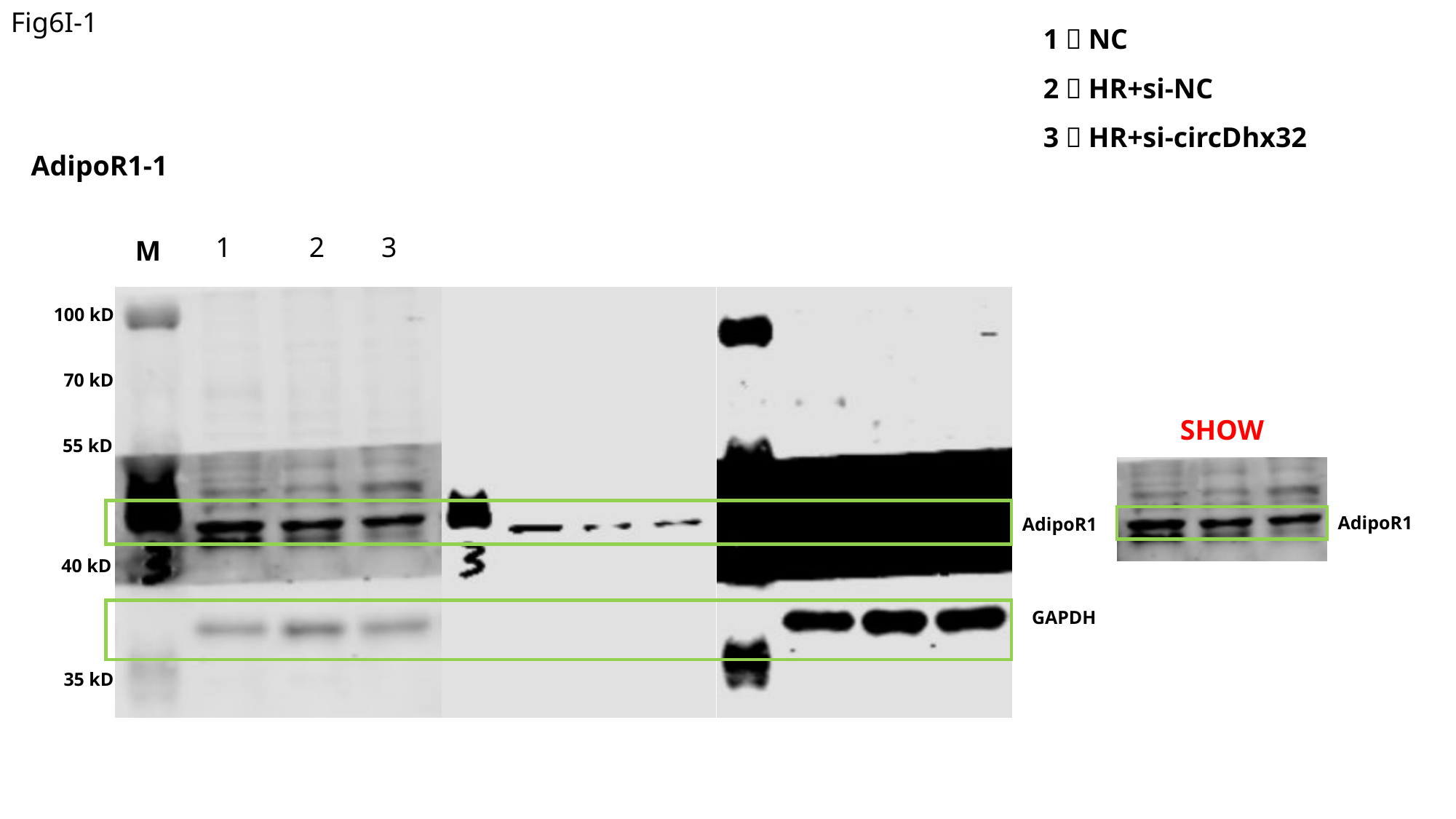

Fig6I-1
1：NC
2：HR+si-NC
3：HR+si-circDhx32
AdipoR1-1
 1 2 3
M
100 kD
70 kD
SHOW
55 kD
AdipoR1
AdipoR1
40 kD
GAPDH
35 kD

## Slide 44
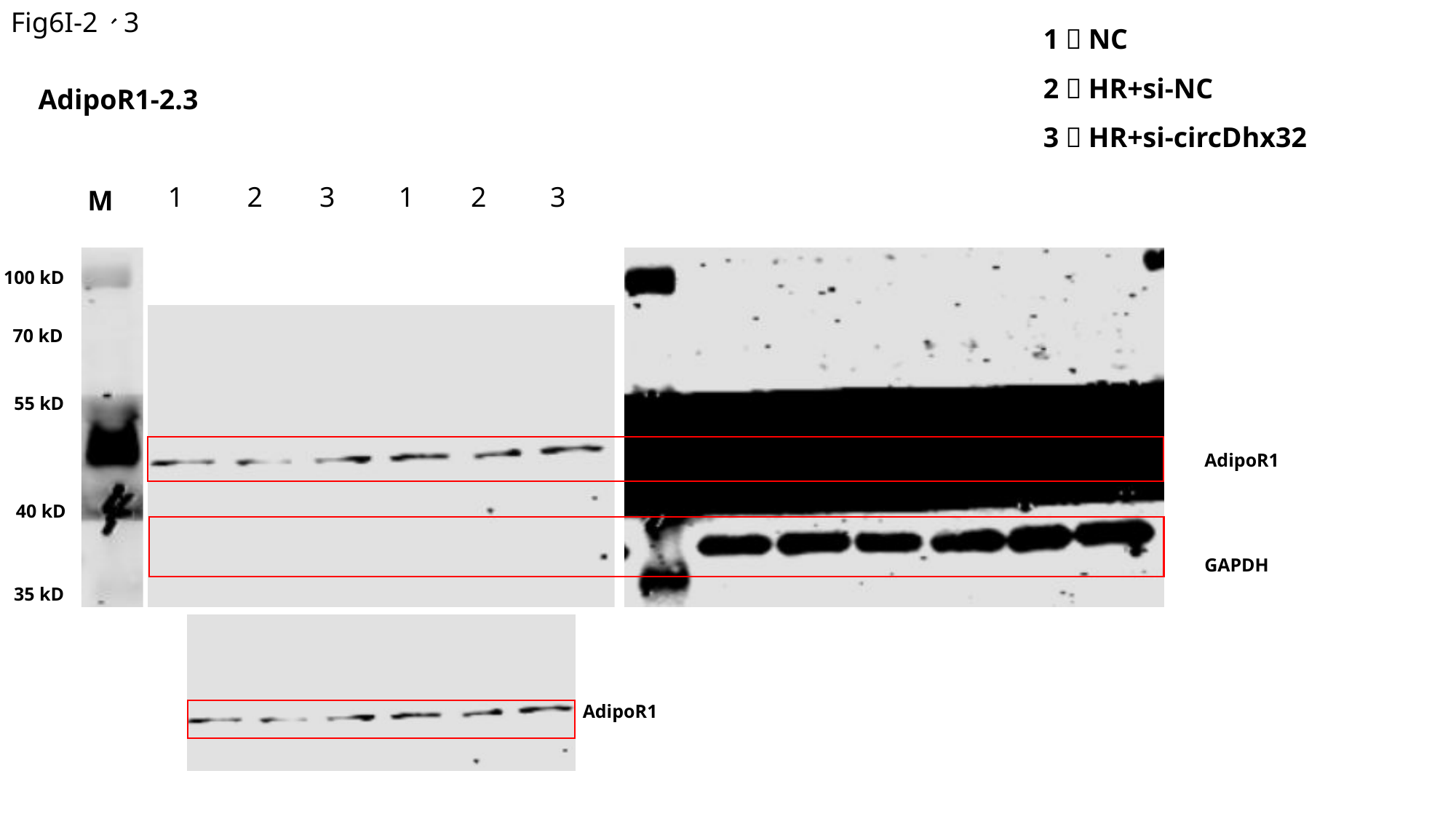

Fig6I-2、3
1：NC
2：HR+si-NC
3：HR+si-circDhx32
AdipoR1-2.3
 1 2 3 1 2 3
M
100 kD
70 kD
55 kD
AdipoR1
40 kD
GAPDH
35 kD
AdipoR1

## Slide 45
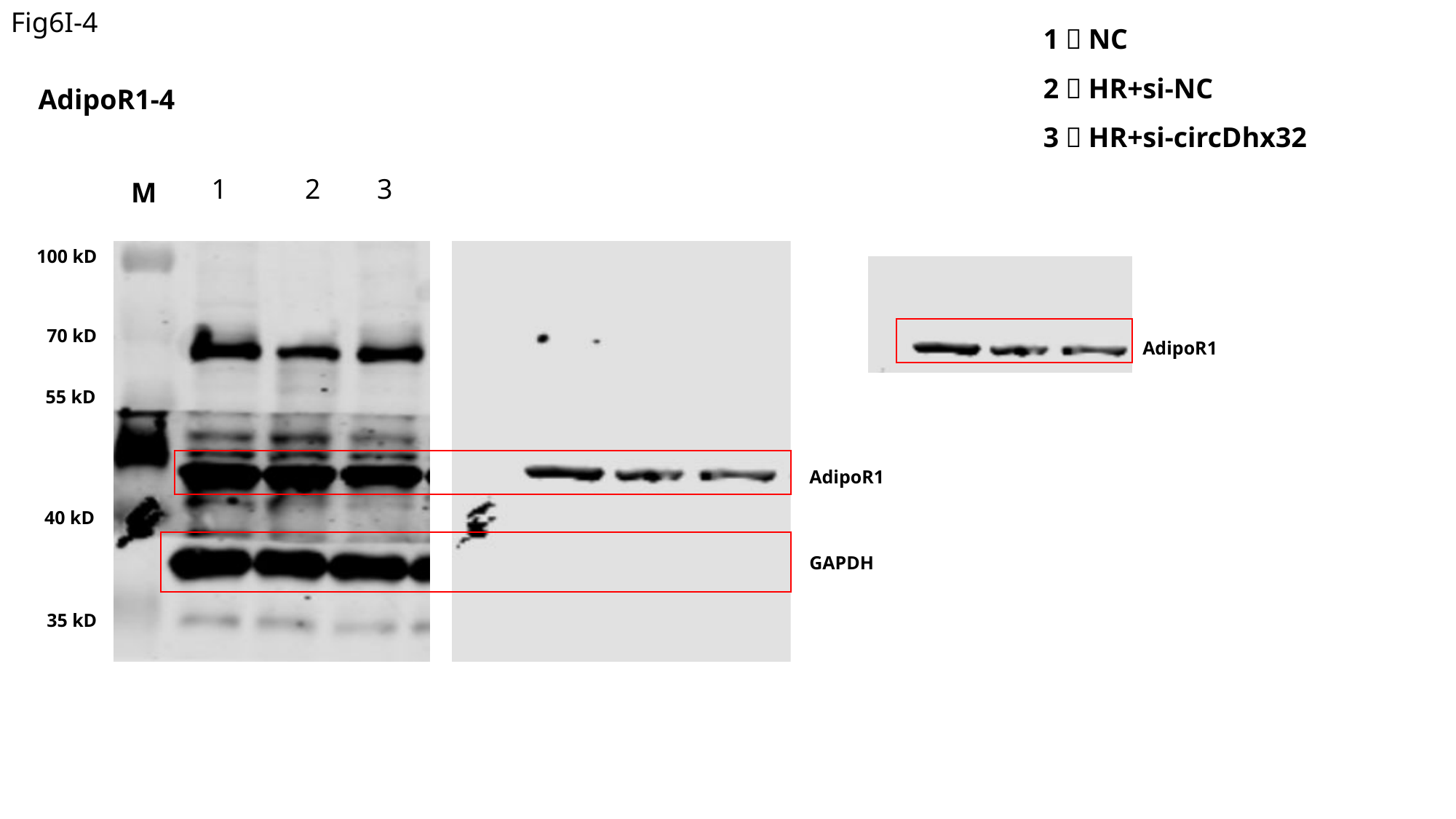

Fig6I-4
1：NC
2：HR+si-NC
3：HR+si-circDhx32
AdipoR1-4
 1 2 3
M
100 kD
70 kD
AdipoR1
55 kD
AdipoR1
40 kD
GAPDH
35 kD

## Slide 46
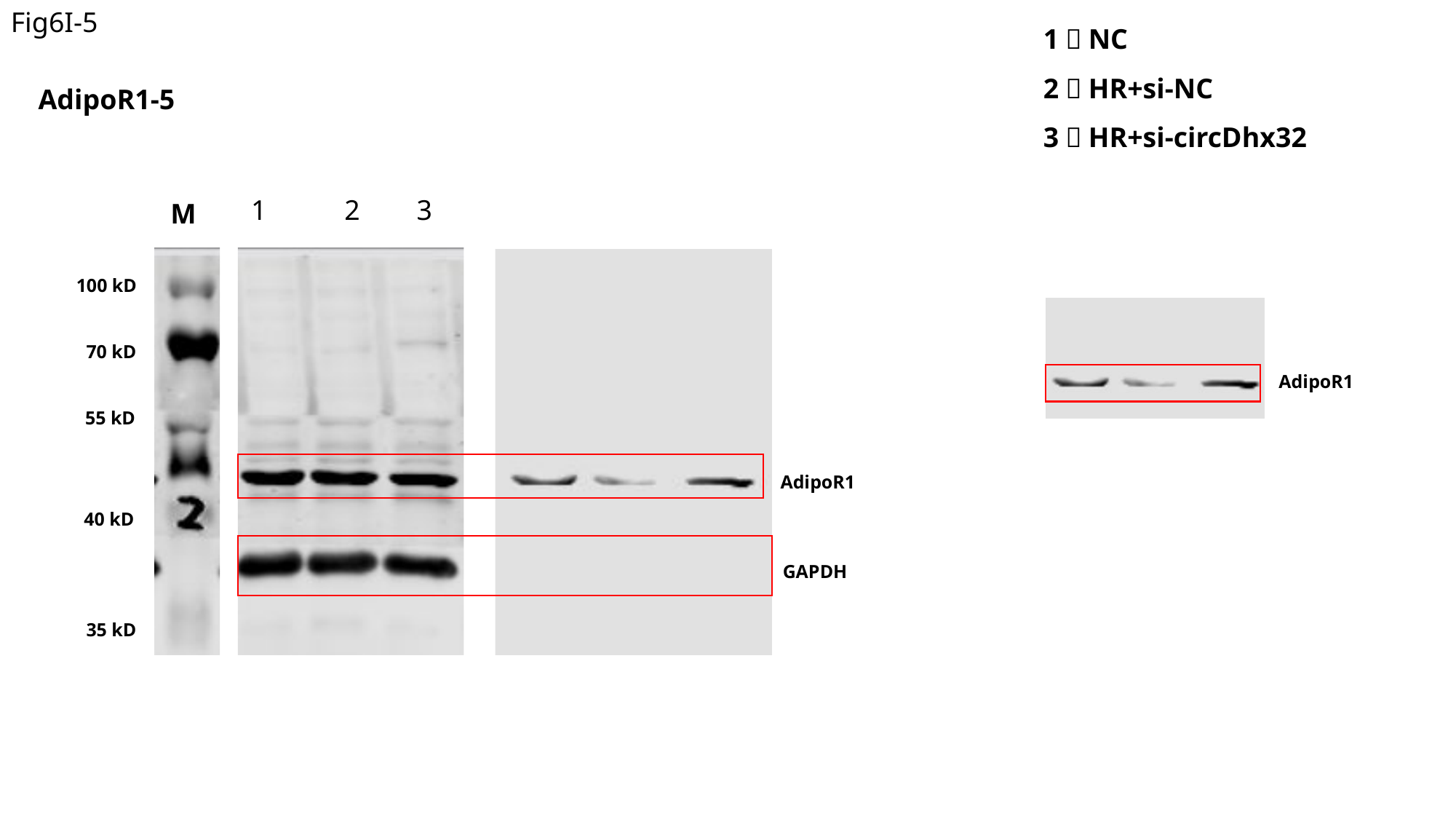

Fig6I-5
1：NC
2：HR+si-NC
3：HR+si-circDhx32
AdipoR1-5
 1 2 3
M
100 kD
70 kD
AdipoR1
55 kD
AdipoR1
40 kD
GAPDH
35 kD

## Slide 47
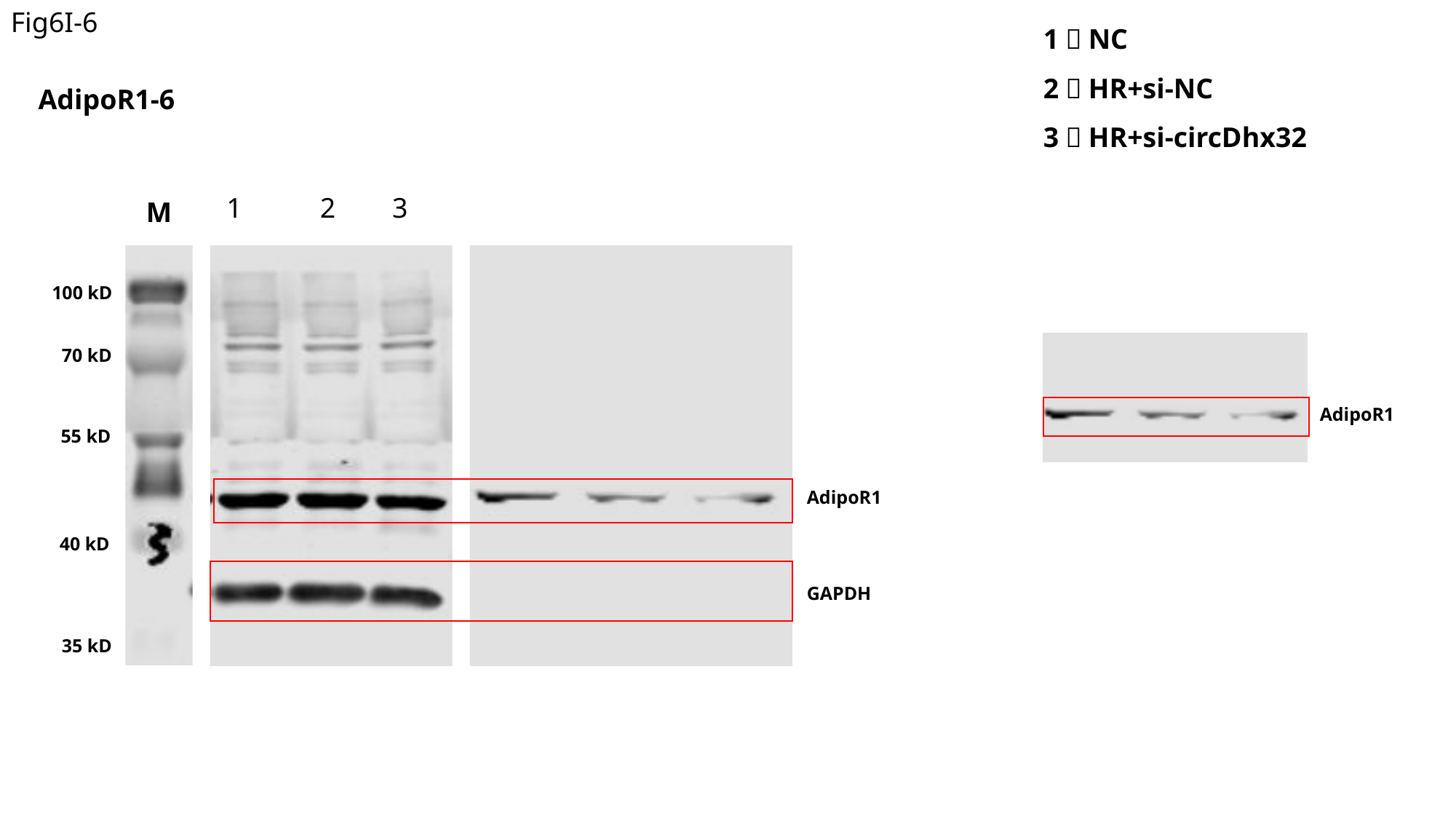

Fig6I-6
1：NC
2：HR+si-NC
3：HR+si-circDhx32
AdipoR1-6
 1 2 3
M
100 kD
70 kD
AdipoR1
55 kD
AdipoR1
40 kD
GAPDH
35 kD

## Slide 48
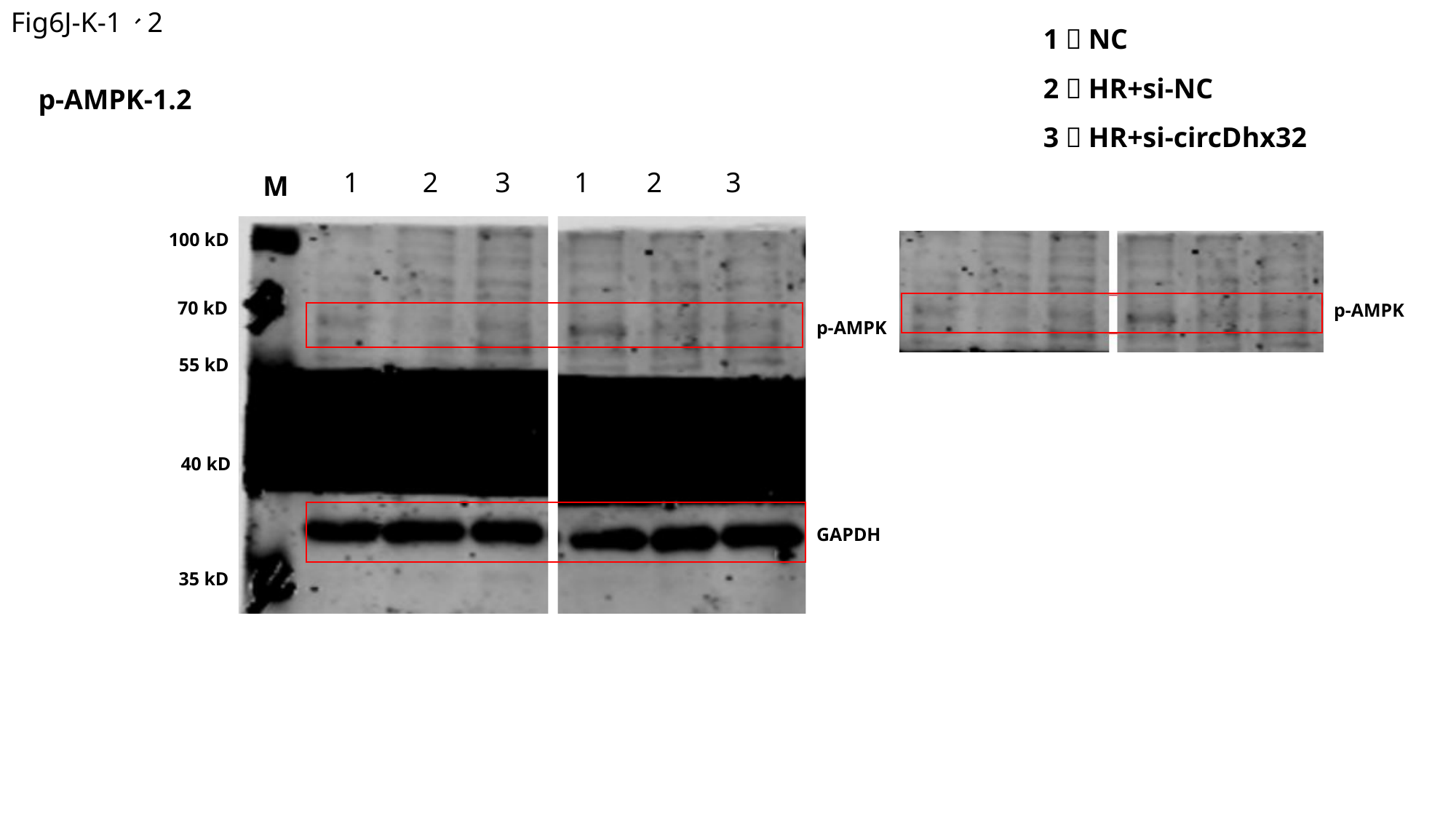

Fig6J-K-1、2
1：NC
2：HR+si-NC
3：HR+si-circDhx32
p-AMPK-1.2
 1 2 3 1 2 3
M
100 kD
70 kD
p-AMPK
p-AMPK
55 kD
40 kD
GAPDH
35 kD

## Slide 49
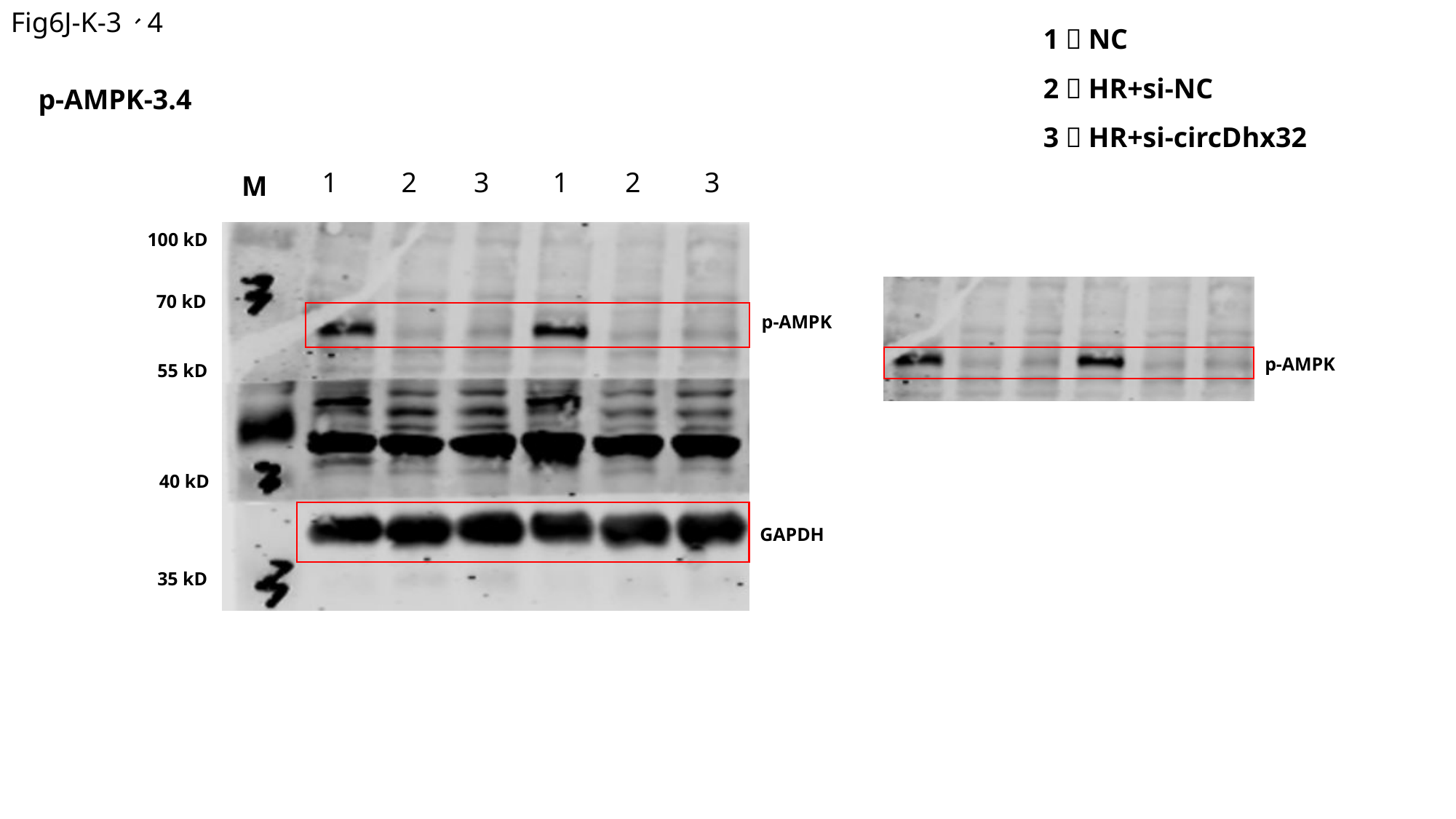

Fig6J-K-3、4
1：NC
2：HR+si-NC
3：HR+si-circDhx32
p-AMPK-3.4
 1 2 3 1 2 3
M
100 kD
70 kD
p-AMPK
p-AMPK
55 kD
40 kD
GAPDH
35 kD

## Slide 50
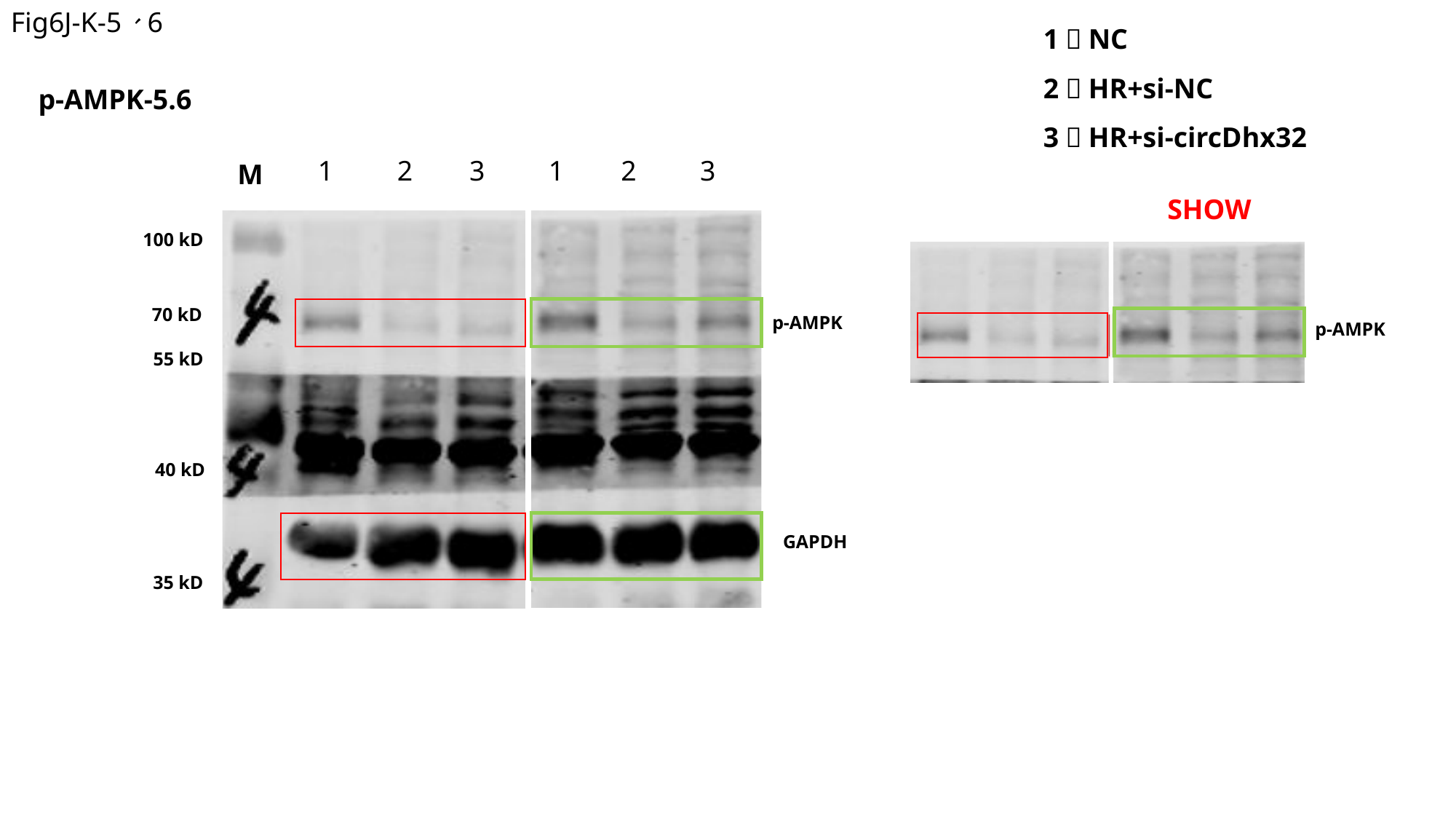

Fig6J-K-5、6
1：NC
2：HR+si-NC
3：HR+si-circDhx32
p-AMPK-5.6
 1 2 3 1 2 3
M
SHOW
100 kD
70 kD
p-AMPK
p-AMPK
55 kD
40 kD
GAPDH
35 kD

## Slide 51
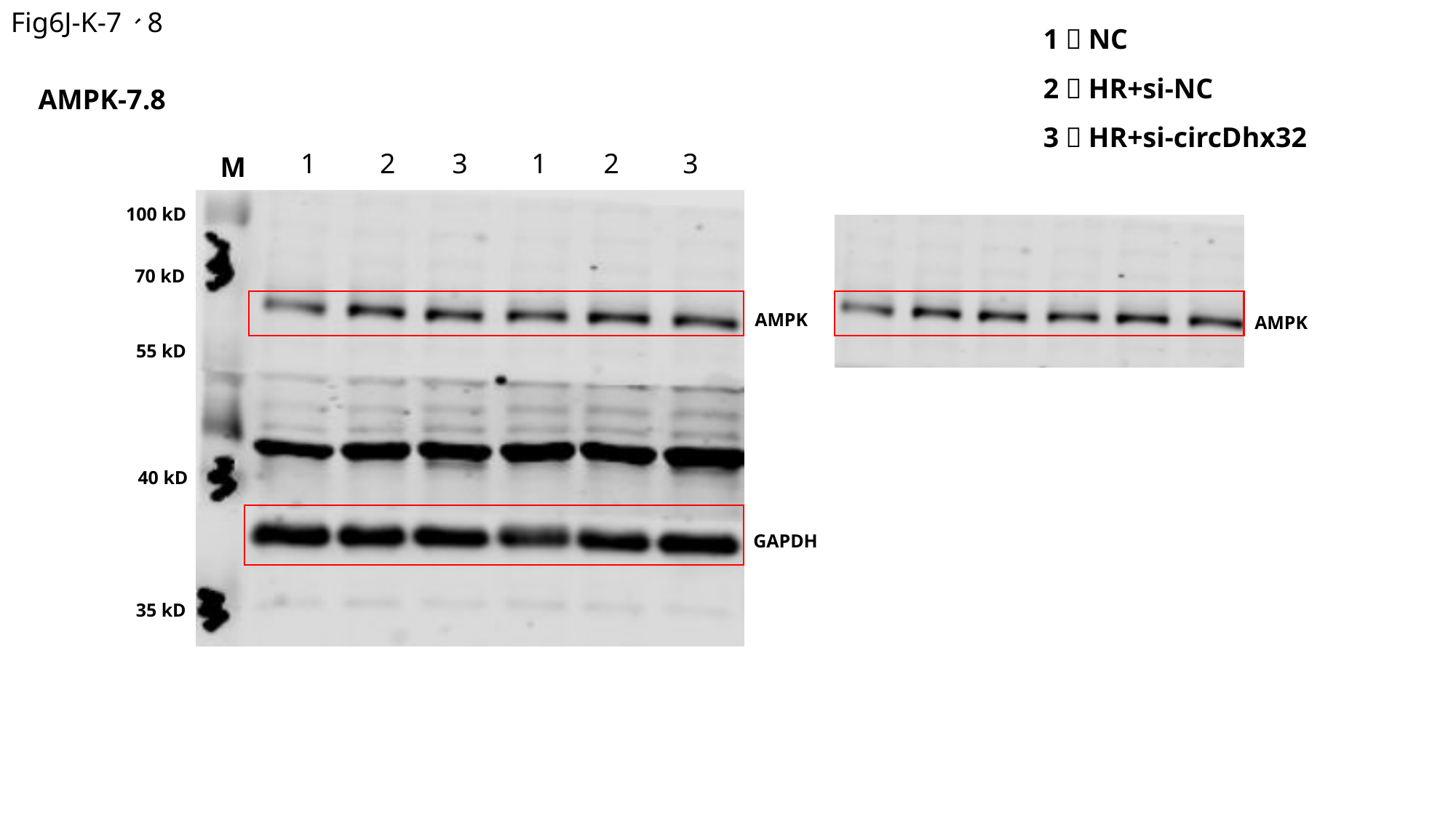

Fig6J-K-7、8
1：NC
2：HR+si-NC
3：HR+si-circDhx32
AMPK-7.8
 1 2 3 1 2 3
M
100 kD
70 kD
AMPK
AMPK
55 kD
40 kD
GAPDH
35 kD

## Slide 52
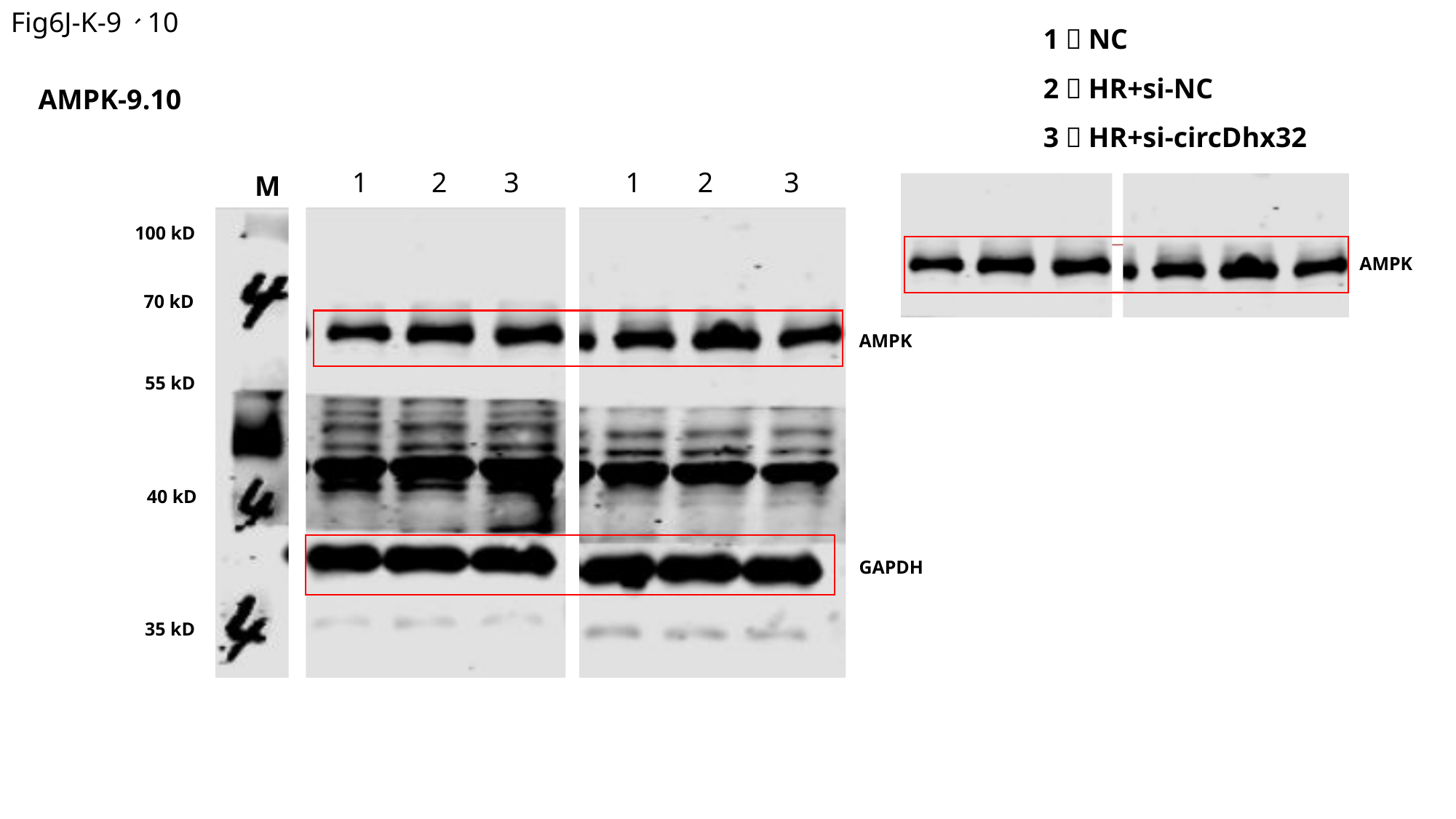

Fig6J-K-9、10
1：NC
2：HR+si-NC
3：HR+si-circDhx32
AMPK-9.10
 1 2 3 1 2 3
M
100 kD
AMPK
70 kD
AMPK
55 kD
40 kD
GAPDH
35 kD

## Slide 53
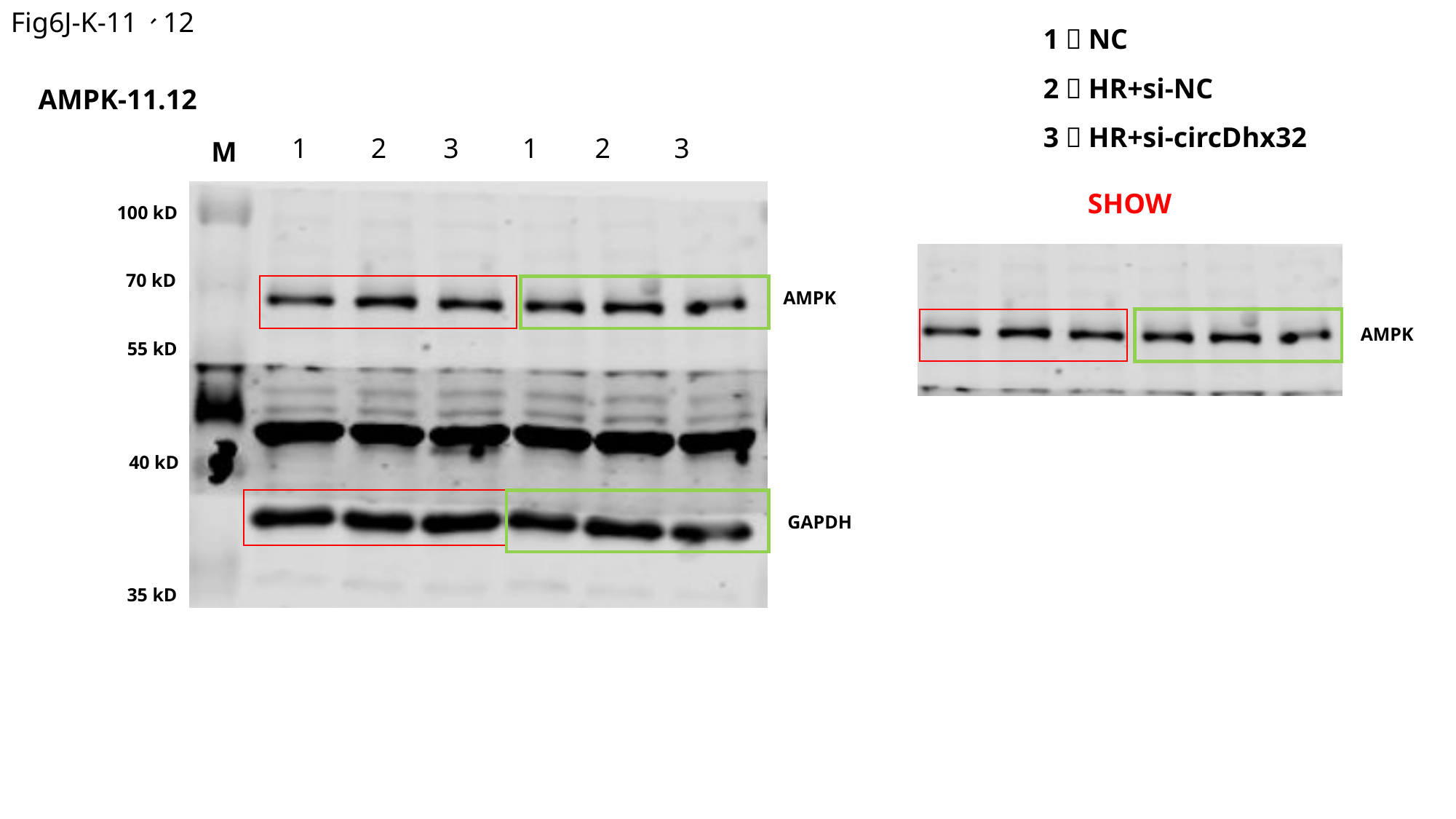

Fig6J-K-11、12
1：NC
2：HR+si-NC
3：HR+si-circDhx32
AMPK-11.12
 1 2 3 1 2 3
M
SHOW
100 kD
70 kD
AMPK
AMPK
55 kD
40 kD
GAPDH
35 kD

## Slide 54
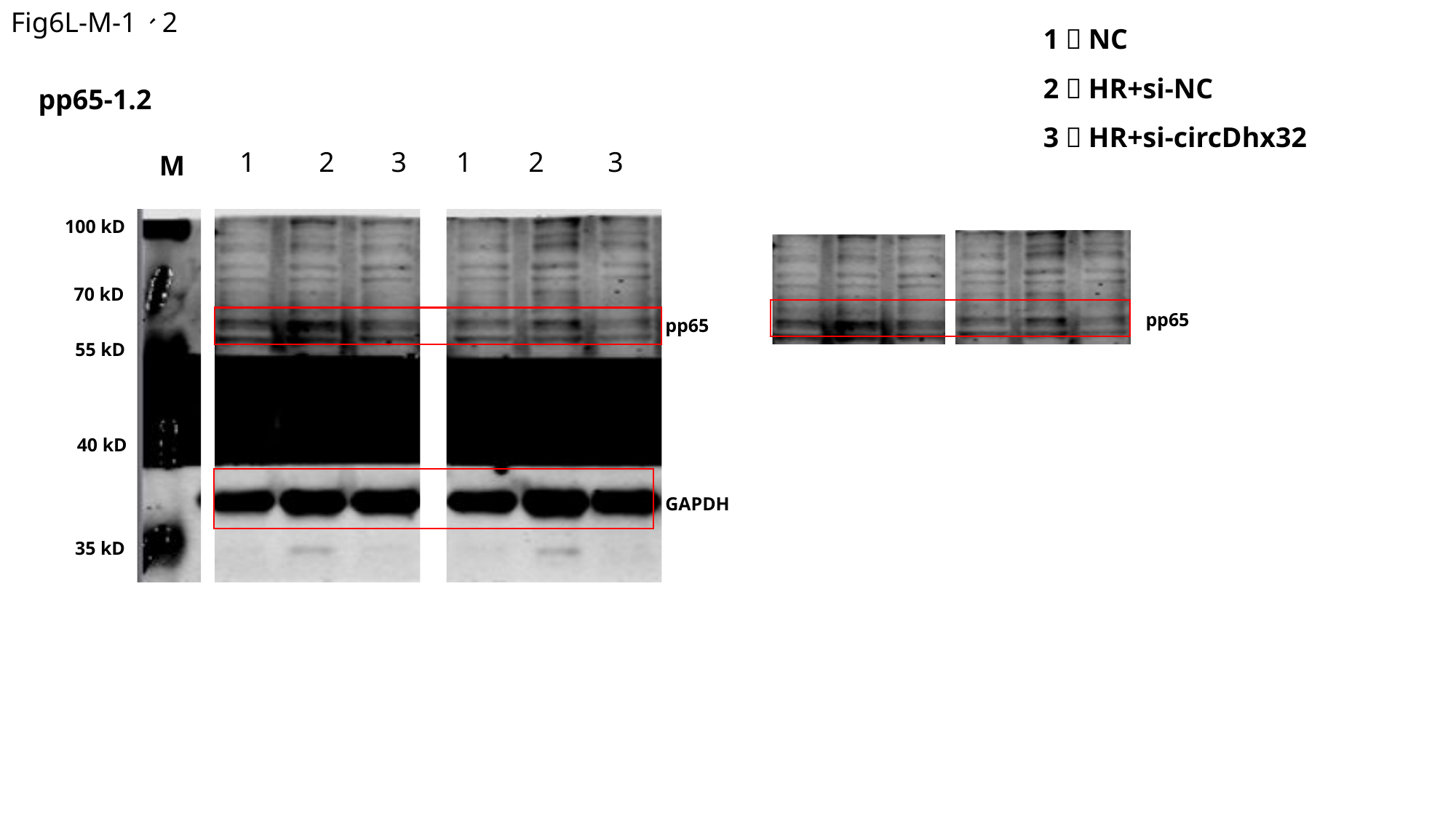

Fig6L-M-1、2
1：NC
2：HR+si-NC
3：HR+si-circDhx32
pp65-1.2
 1 2 3 1 2 3
M
100 kD
70 kD
pp65
pp65
55 kD
40 kD
GAPDH
35 kD

## Slide 55
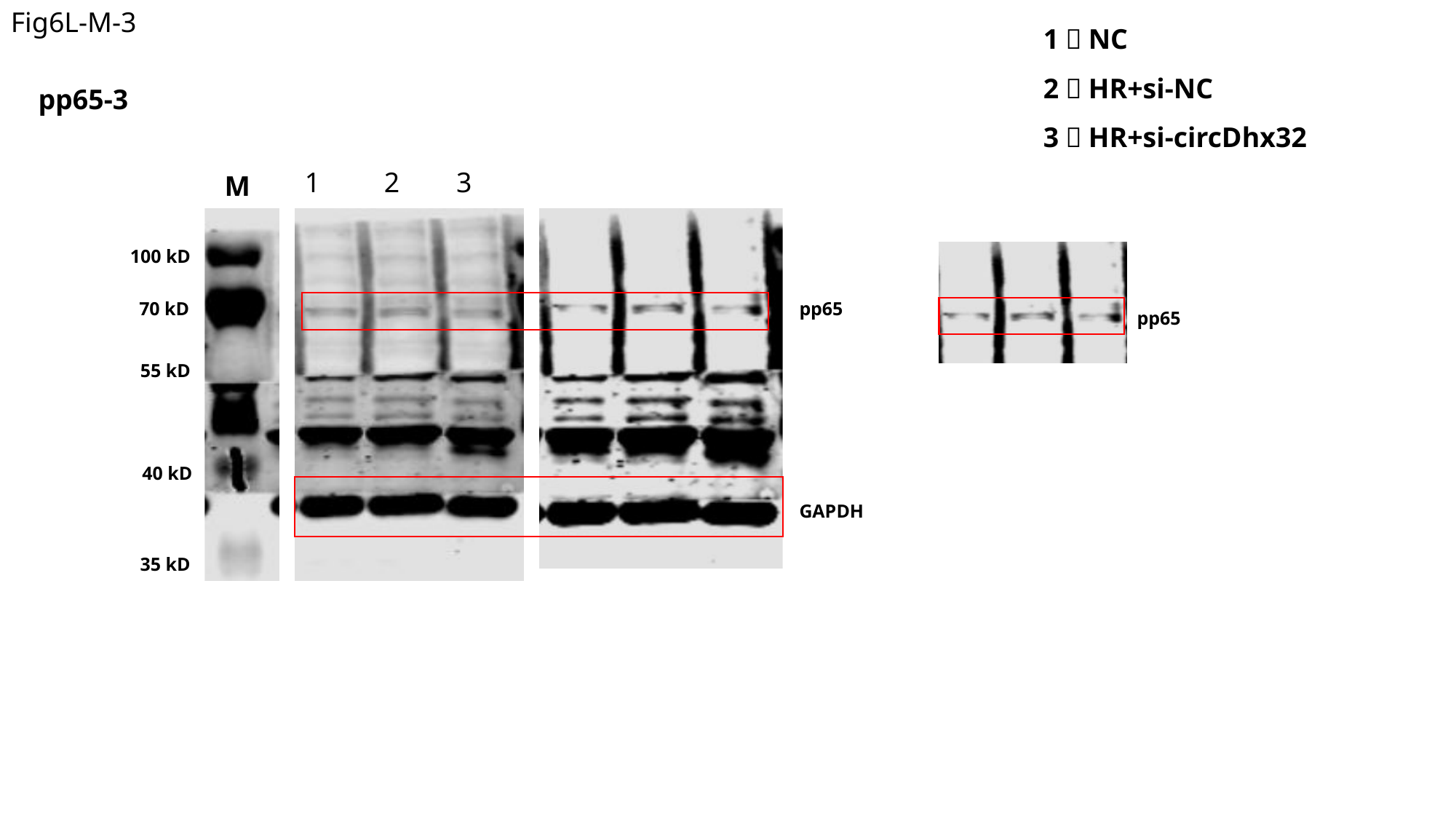

Fig6L-M-3
1：NC
2：HR+si-NC
3：HR+si-circDhx32
pp65-3
 1 2 3
M
100 kD
70 kD
pp65
pp65
55 kD
40 kD
GAPDH
35 kD

## Slide 56
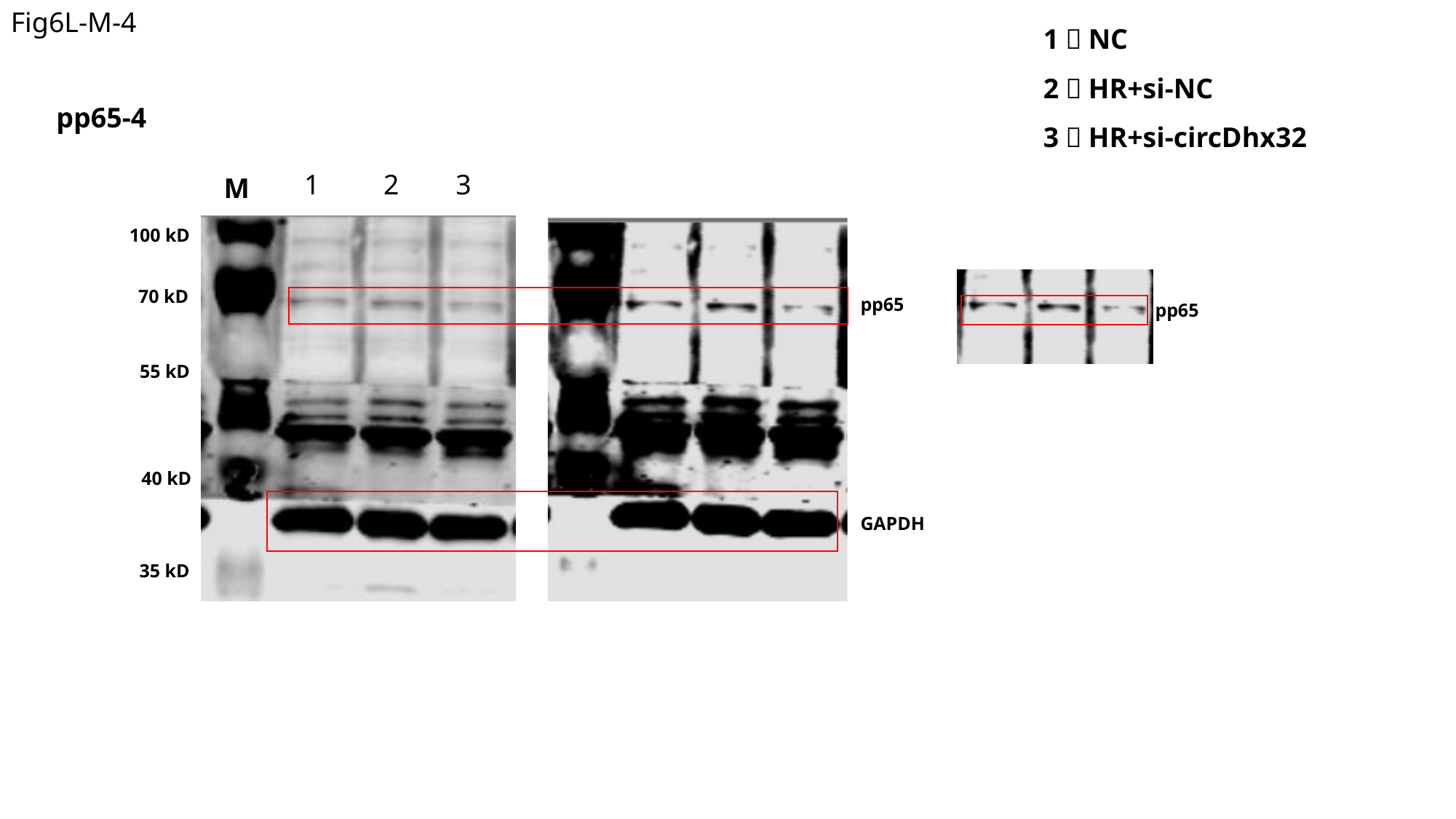

Fig6L-M-4
1：NC
2：HR+si-NC
3：HR+si-circDhx32
pp65-4
 1 2 3
M
100 kD
70 kD
pp65
pp65
55 kD
40 kD
GAPDH
35 kD

## Slide 57
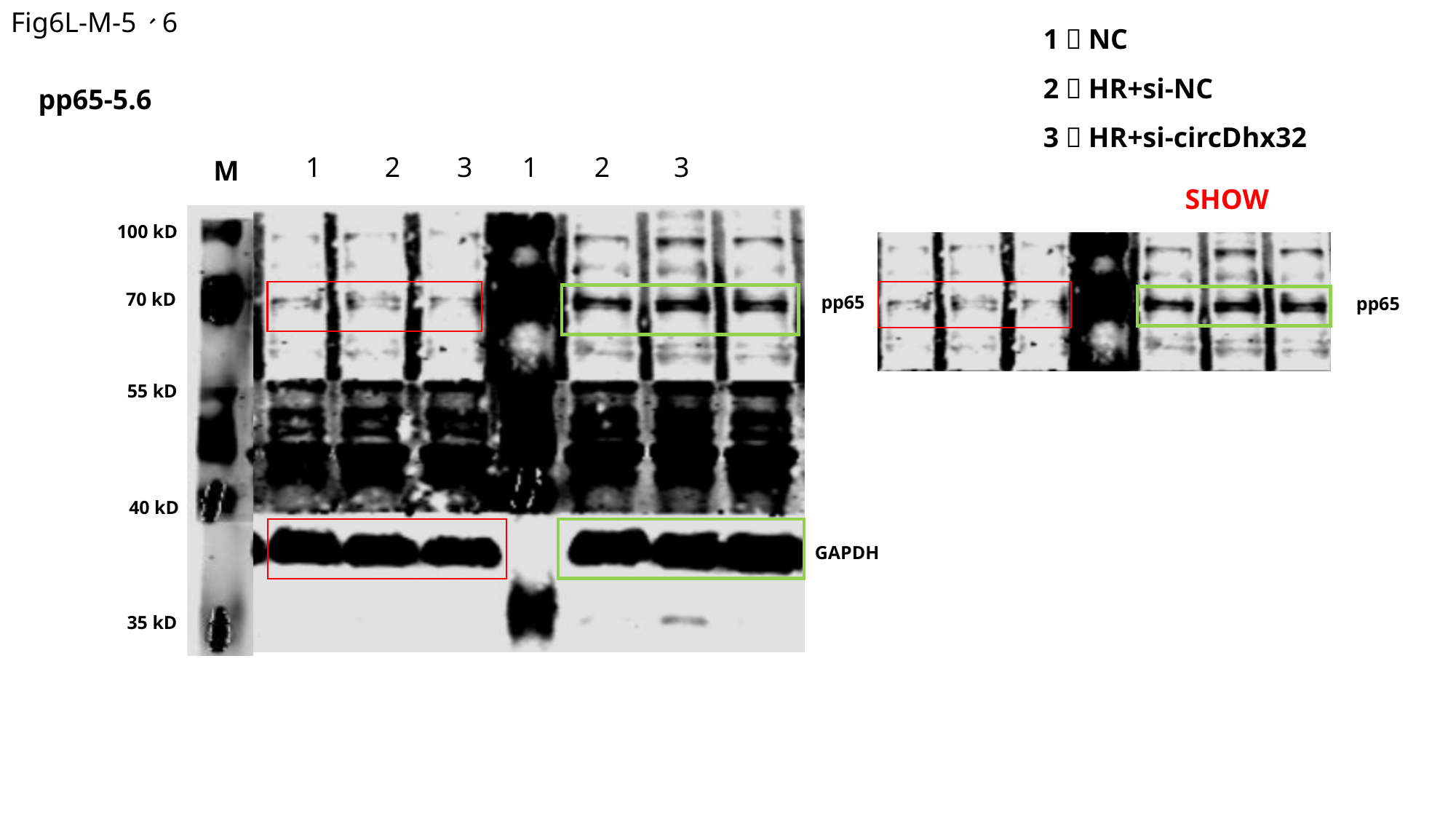

Fig6L-M-5、6
1：NC
2：HR+si-NC
3：HR+si-circDhx32
pp65-5.6
 1 2 3 1 2 3
M
SHOW
100 kD
70 kD
pp65
pp65
55 kD
40 kD
GAPDH
35 kD

## Slide 58
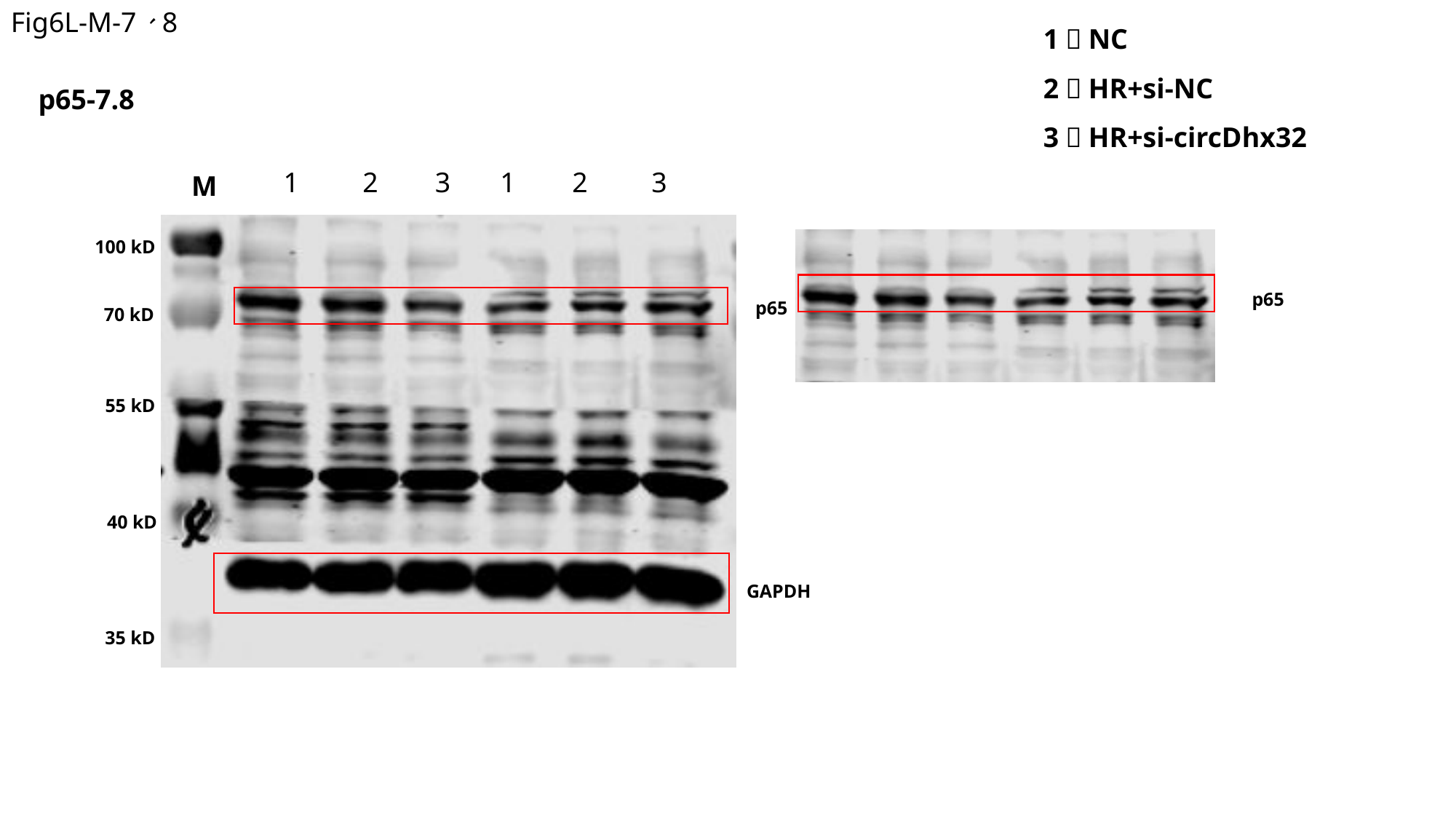

Fig6L-M-7、8
1：NC
2：HR+si-NC
3：HR+si-circDhx32
p65-7.8
 1 2 3 1 2 3
M
100 kD
p65
p65
70 kD
55 kD
40 kD
GAPDH
35 kD

## Slide 59
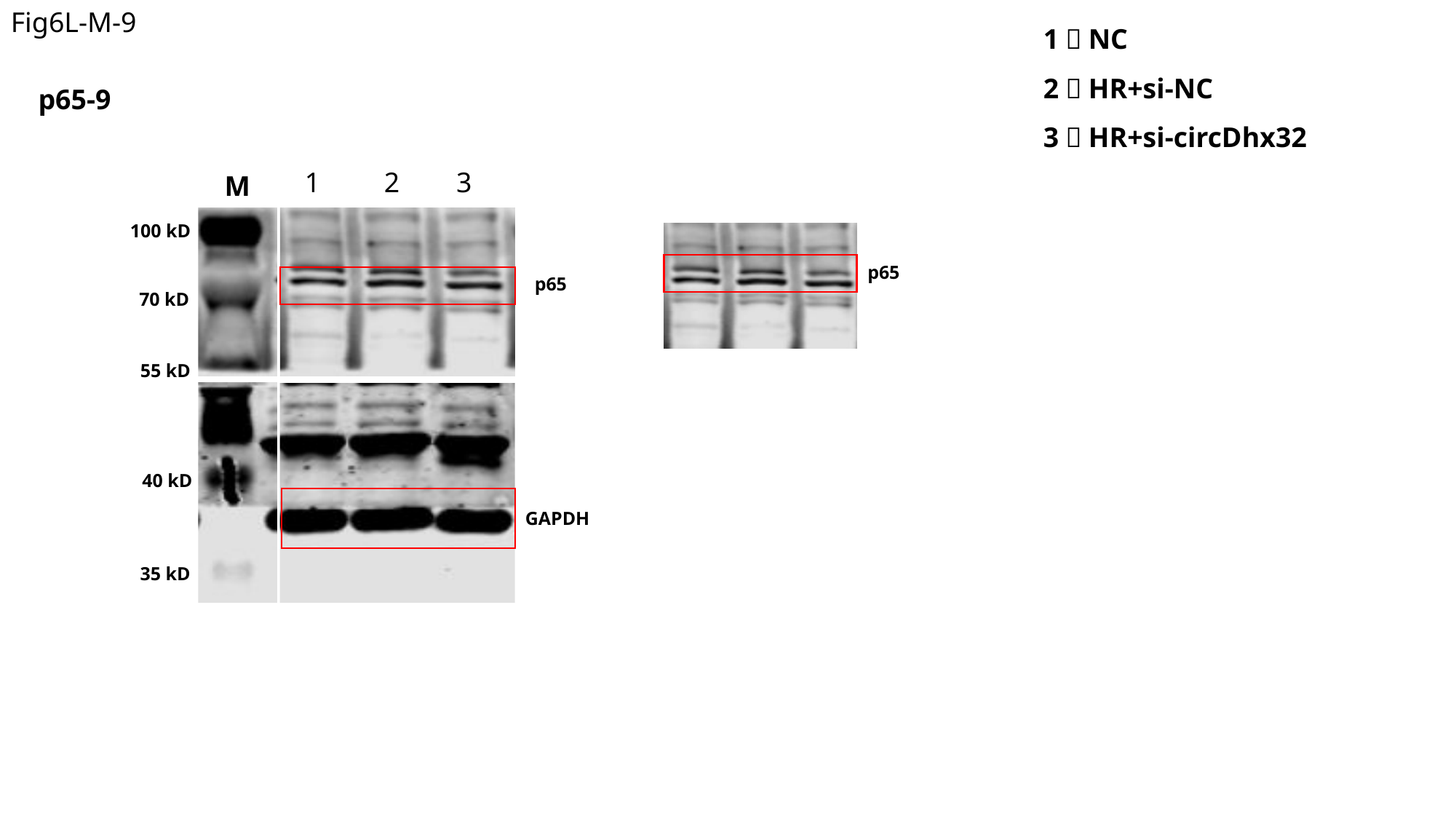

Fig6L-M-9
1：NC
2：HR+si-NC
3：HR+si-circDhx32
p65-9
 1 2 3
M
100 kD
p65
p65
70 kD
55 kD
40 kD
GAPDH
35 kD

## Slide 60
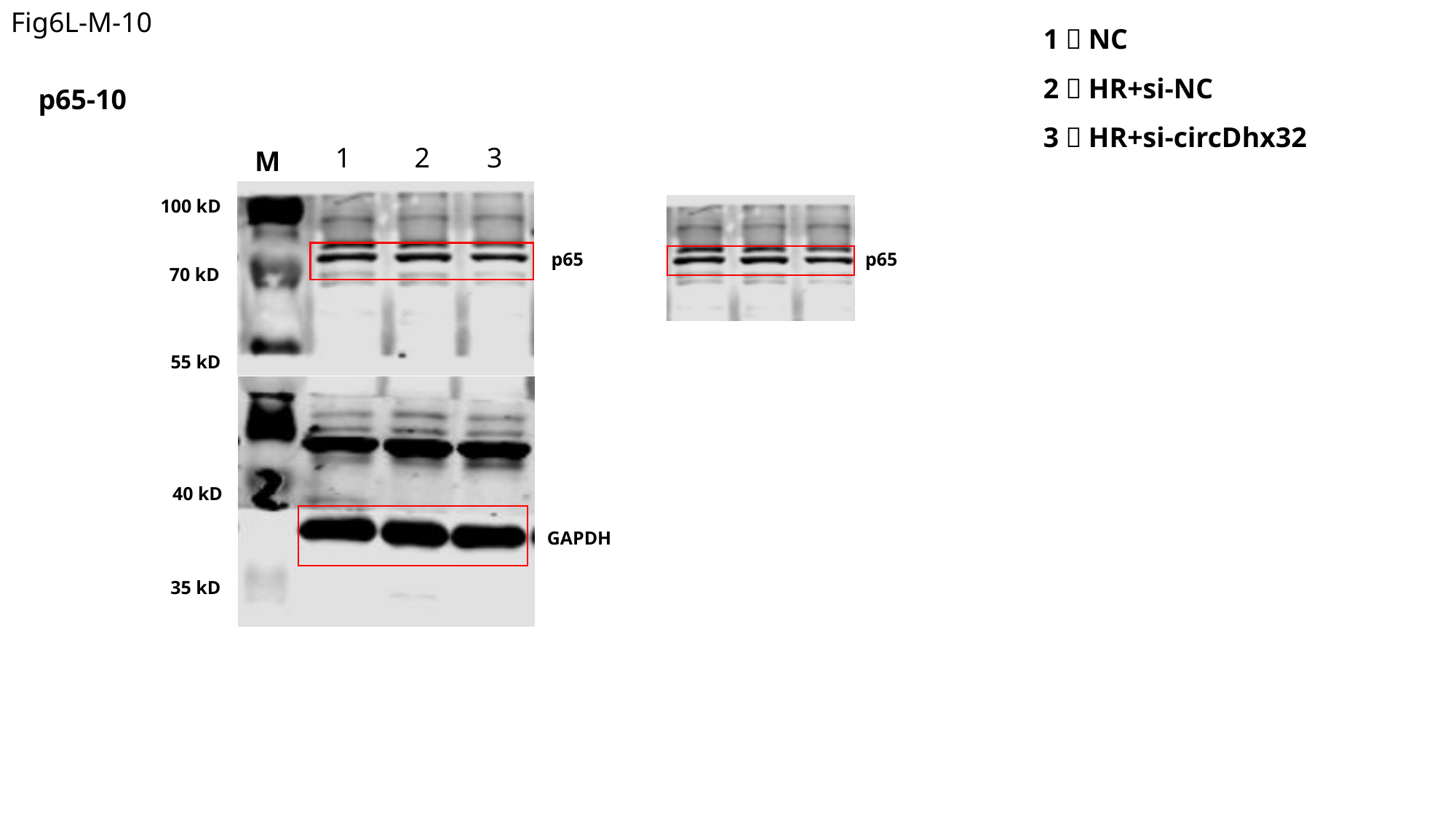

Fig6L-M-10
1：NC
2：HR+si-NC
3：HR+si-circDhx32
p65-10
 1 2 3
M
100 kD
p65
p65
70 kD
55 kD
40 kD
GAPDH
35 kD

## Slide 61
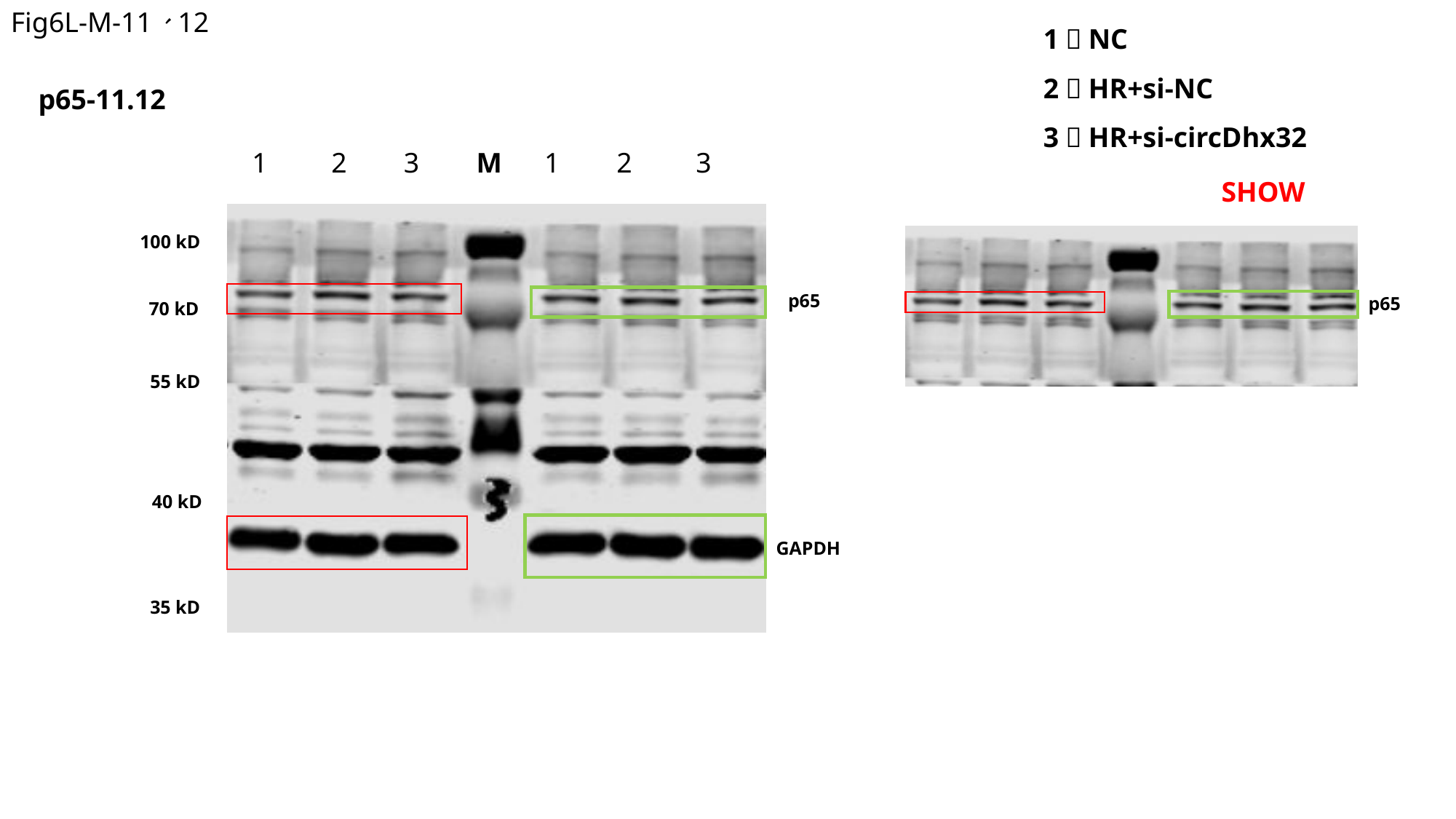

Fig6L-M-11、12
1：NC
2：HR+si-NC
3：HR+si-circDhx32
p65-11.12
 1 2 3 M 1 2 3
SHOW
100 kD
p65
p65
70 kD
55 kD
40 kD
GAPDH
35 kD

## Slide 62
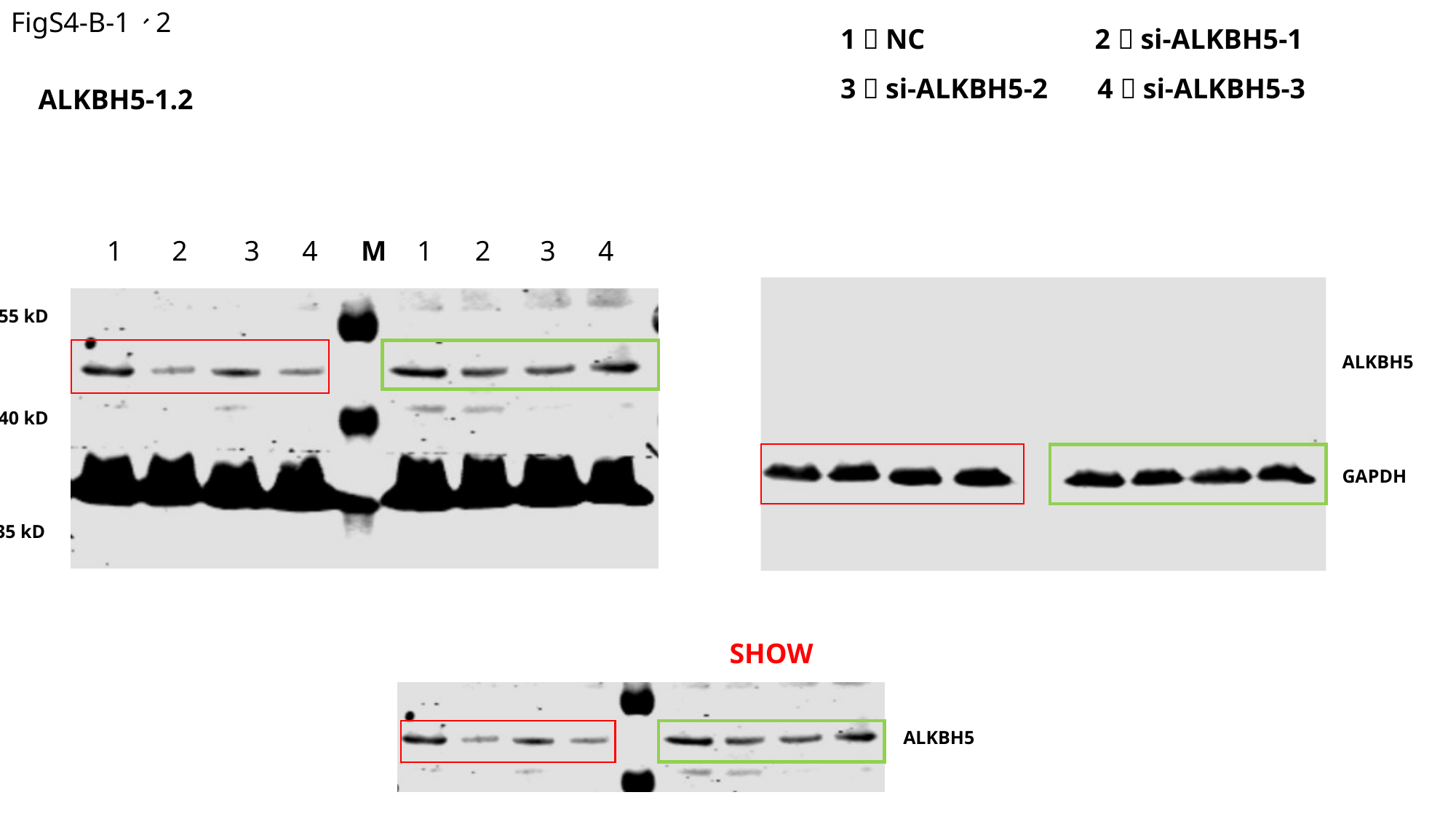

FigS4-B-1、2
1：NC 2：si-ALKBH5-1
3：si-ALKBH5-2 4：si-ALKBH5-3
ALKBH5-1.2
 1 2 3 4 1 2 3 4
M
55 kD
ALKBH5
40 kD
GAPDH
35 kD
SHOW
ALKBH5

## Slide 63
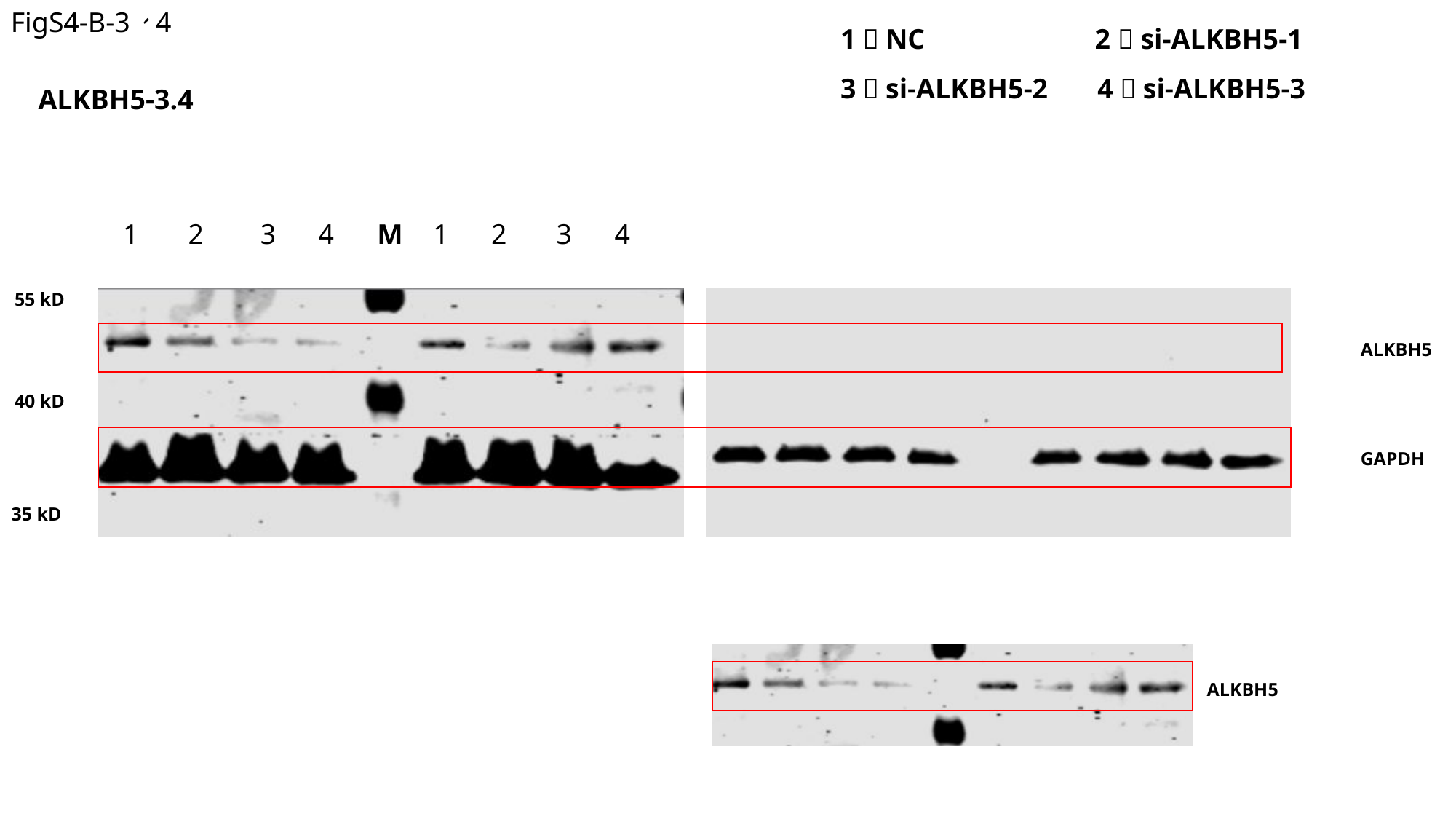

FigS4-B-3、4
1：NC 2：si-ALKBH5-1
3：si-ALKBH5-2 4：si-ALKBH5-3
ALKBH5-3.4
 1 2 3 4 1 2 3 4
M
55 kD
ALKBH5
40 kD
GAPDH
35 kD
ALKBH5

## Slide 64
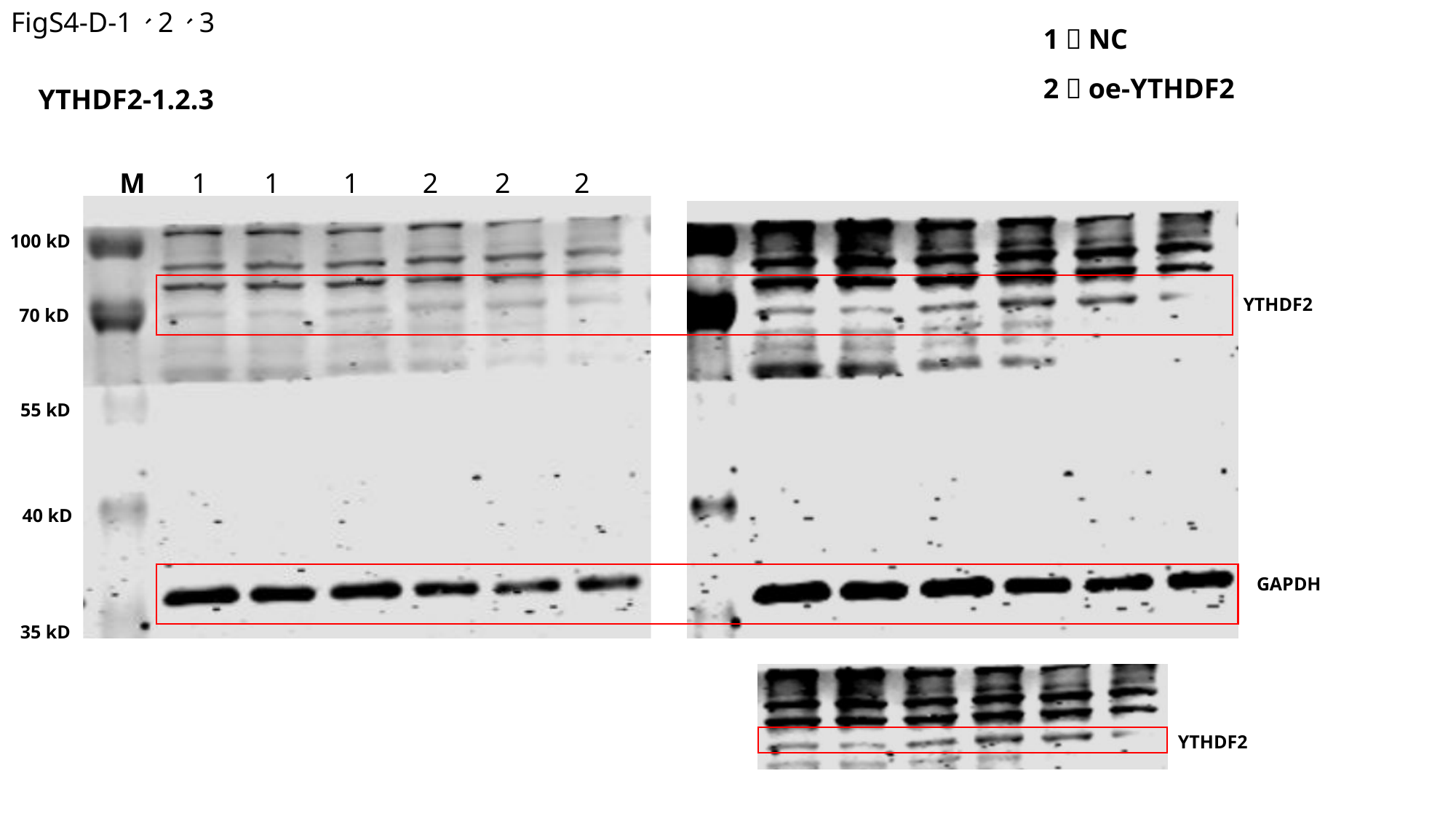

FigS4-D-1、2、3
1：NC
2：oe-YTHDF2
YTHDF2-1.2.3
M
 1 1 1 2 2 2
100 kD
YTHDF2
70 kD
55 kD
40 kD
GAPDH
35 kD
YTHDF2

## Slide 65
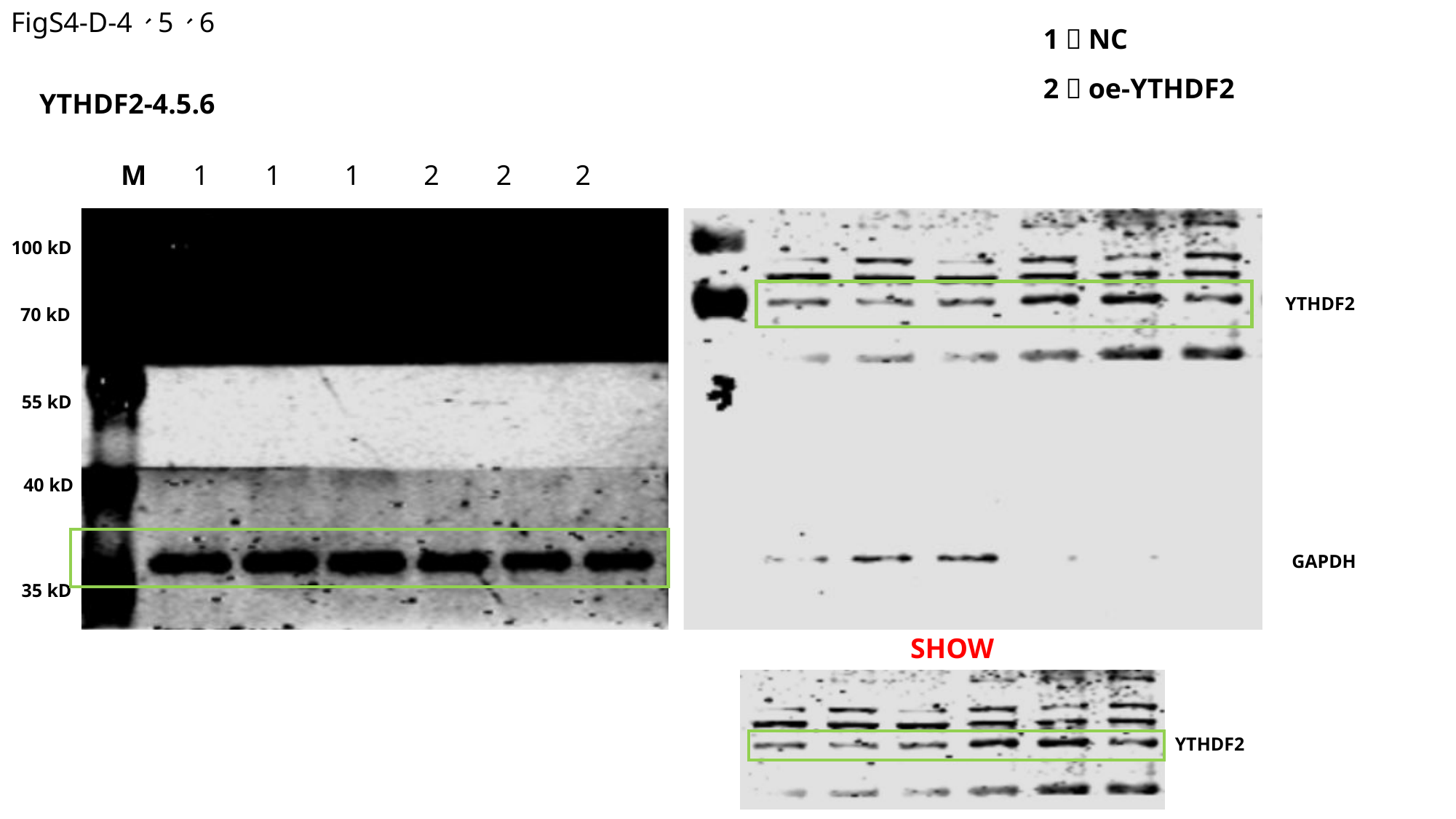

FigS4-D-4、5、6
1：NC
2：oe-YTHDF2
YTHDF2-4.5.6
M
 1 1 1 2 2 2
100 kD
YTHDF2
70 kD
55 kD
40 kD
GAPDH
35 kD
SHOW
YTHDF2

## Slide 66
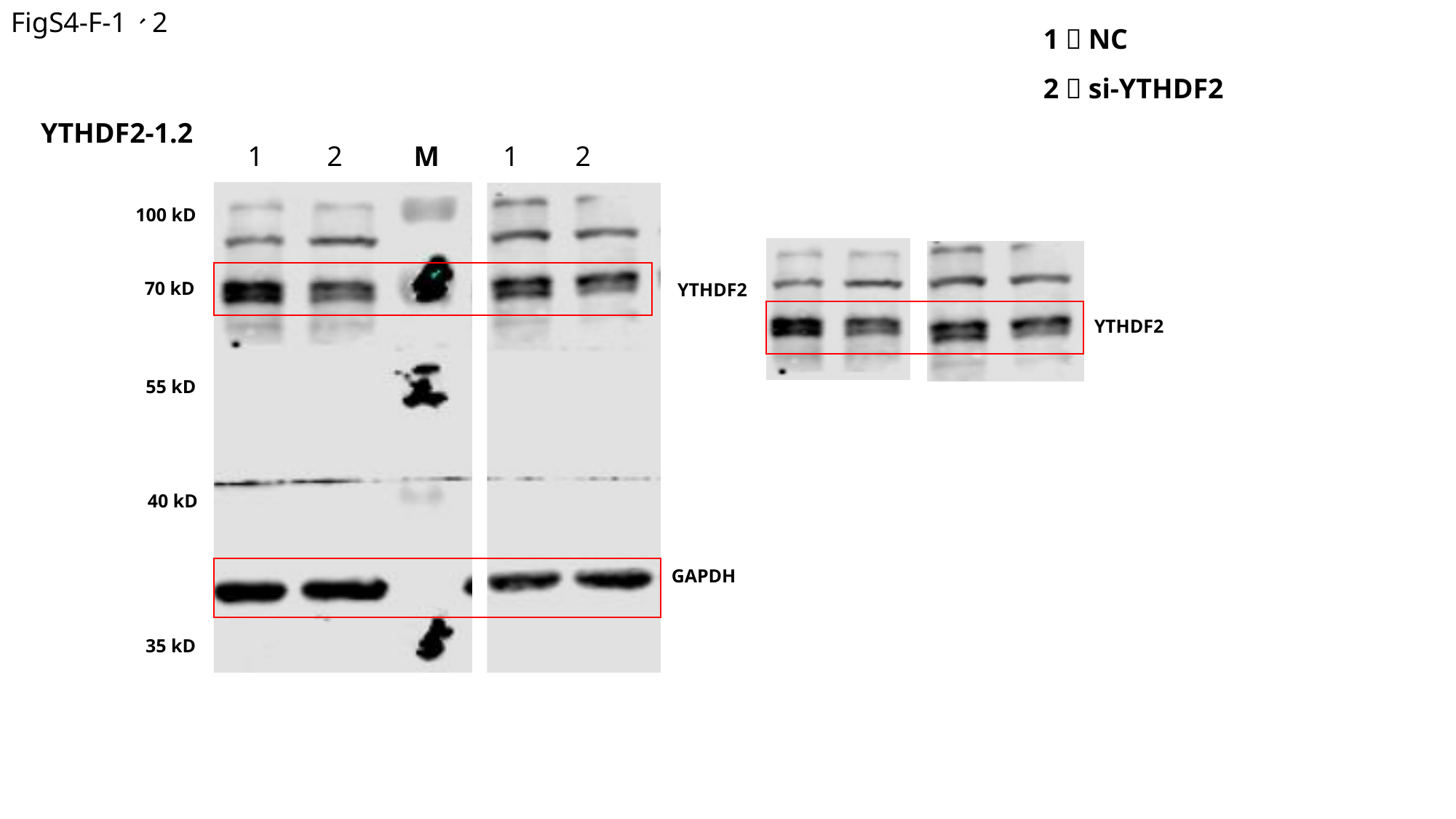

FigS4-F-1、2
1：NC
2：si-YTHDF2
YTHDF2-1.2
 1 2 M 1 2
100 kD
70 kD
YTHDF2
YTHDF2
55 kD
40 kD
GAPDH
35 kD

## Slide 67
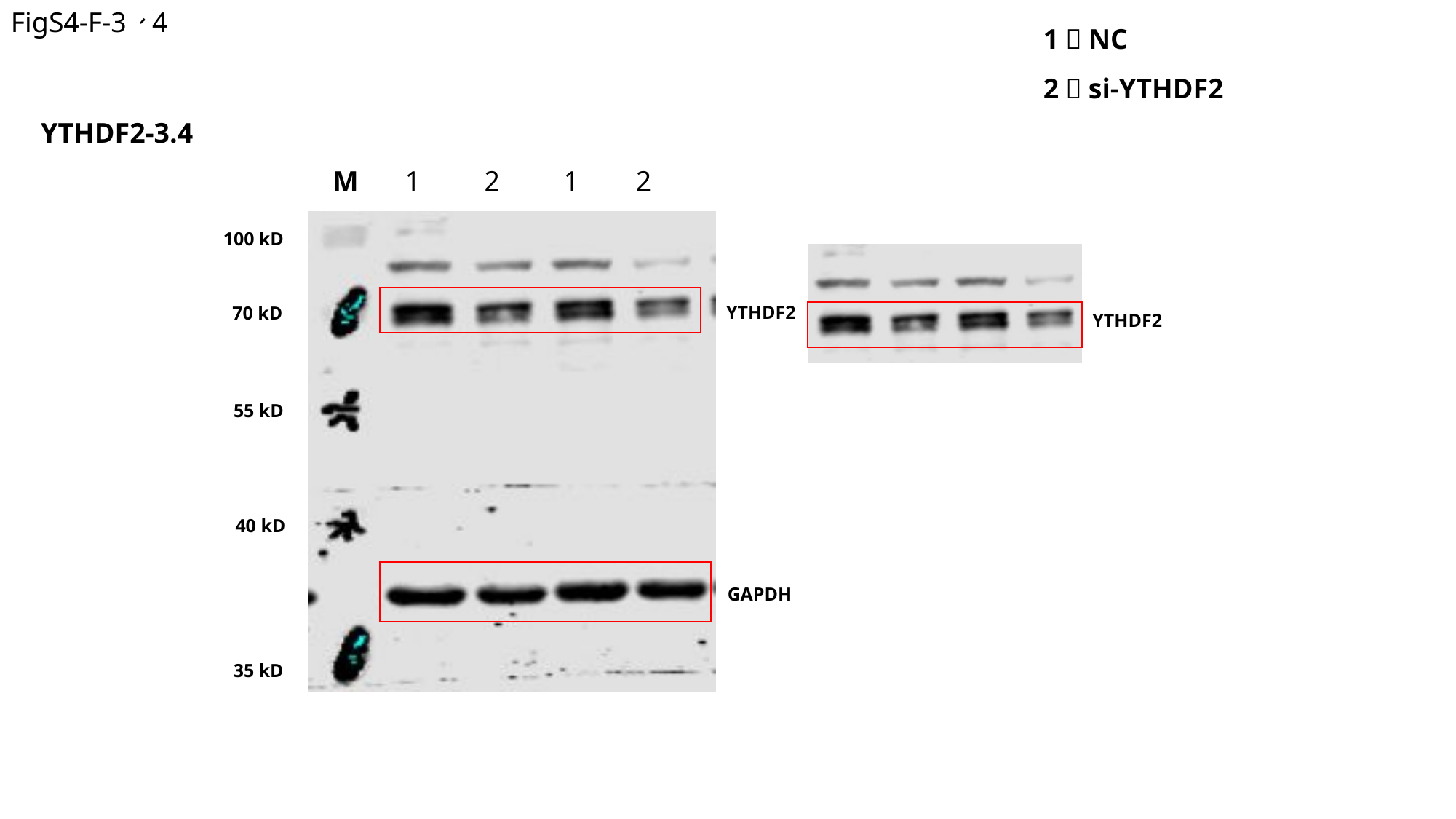

FigS4-F-3、4
1：NC
2：si-YTHDF2
YTHDF2-3.4
M
 1 2 1 2
100 kD
YTHDF2
70 kD
YTHDF2
55 kD
40 kD
GAPDH
35 kD

## Slide 68
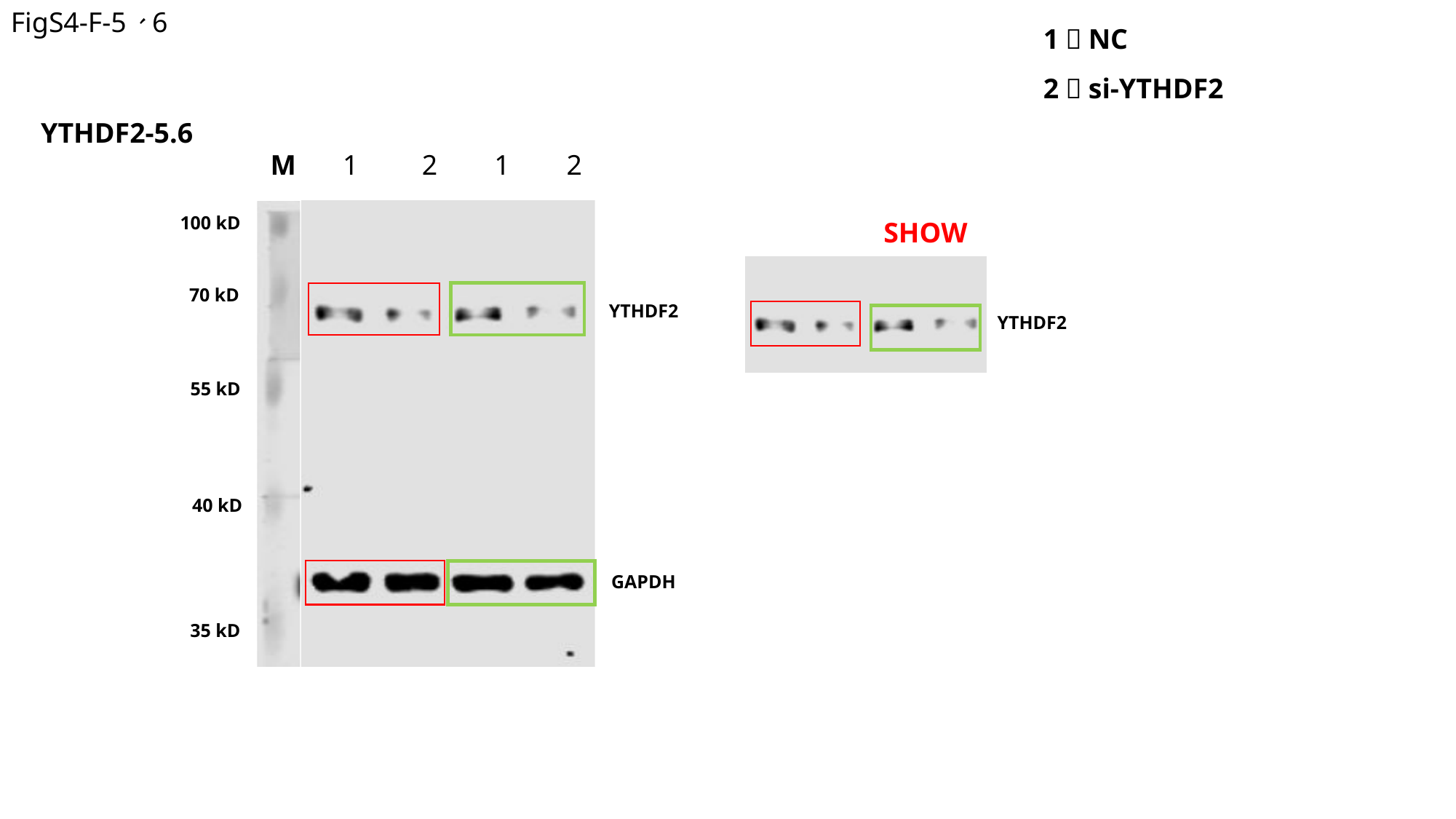

FigS4-F-5、6
1：NC
2：si-YTHDF2
YTHDF2-5.6
M
 1 2 1 2
100 kD
SHOW
70 kD
YTHDF2
YTHDF2
55 kD
40 kD
GAPDH
35 kD

## Slide 69
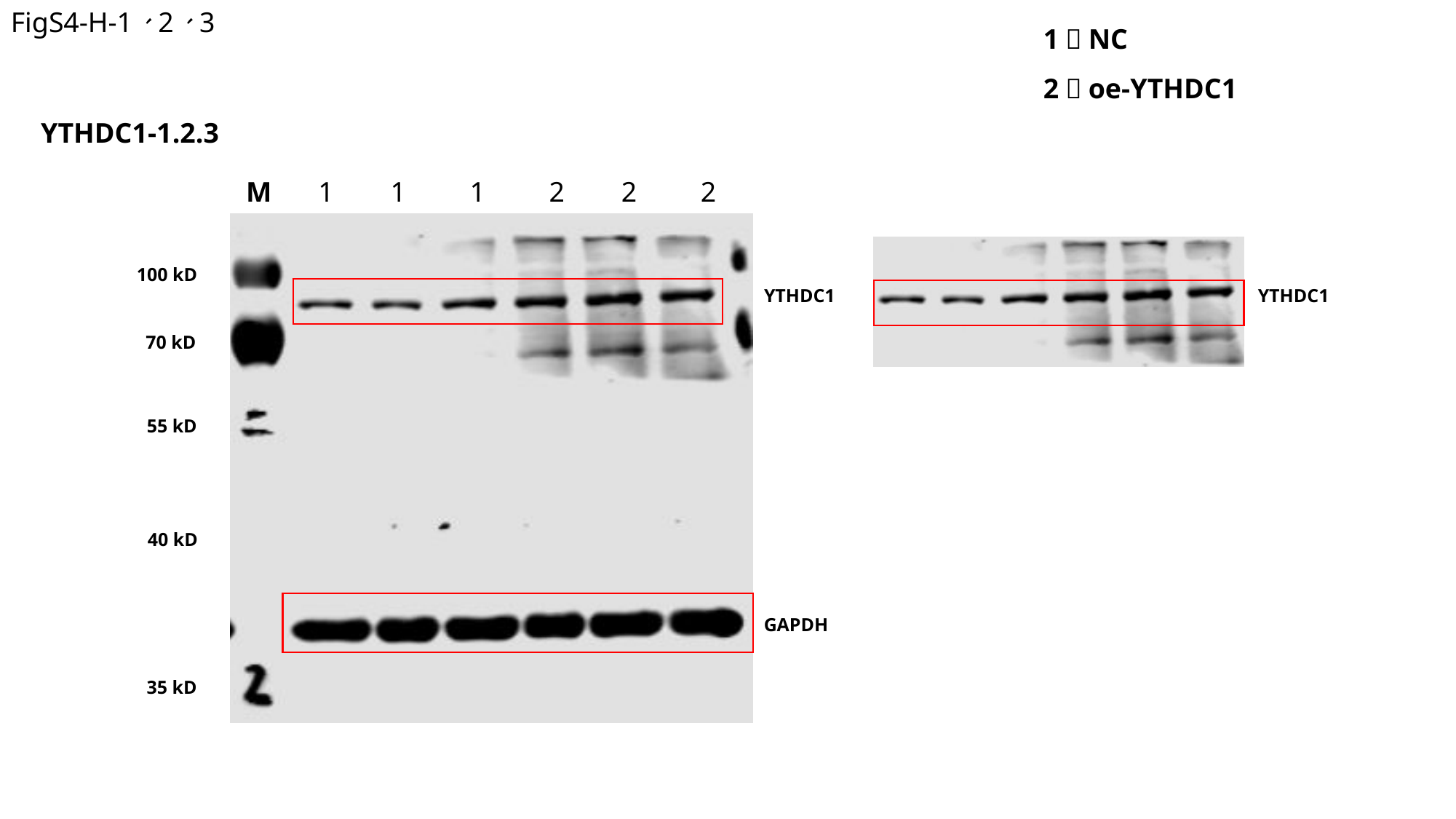

FigS4-H-1、2、3
1：NC
2：oe-YTHDC1
YTHDC1-1.2.3
M
 1 1 1 2 2 2
100 kD
YTHDC1
YTHDC1
70 kD
55 kD
40 kD
GAPDH
35 kD

## Slide 70
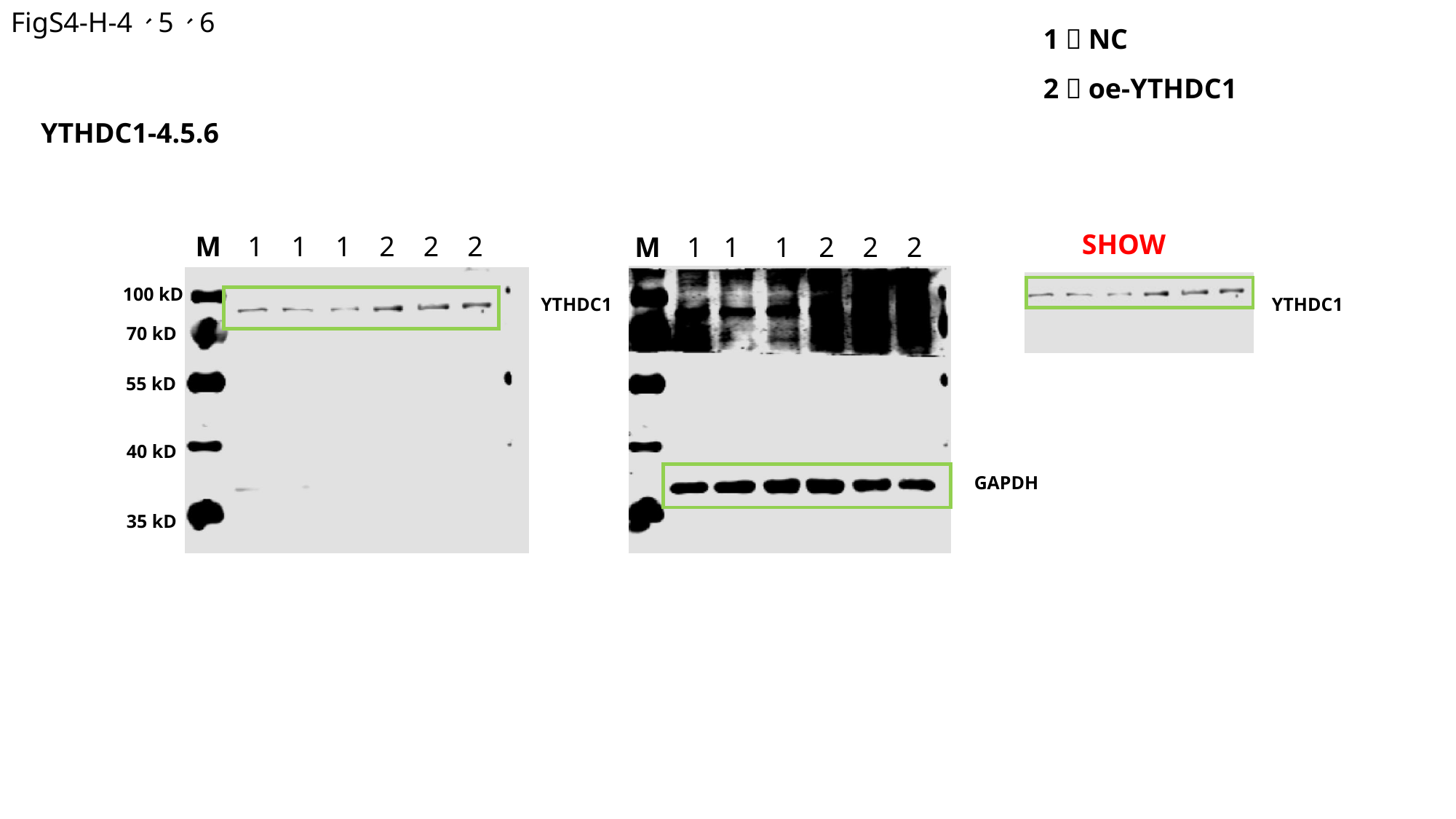

FigS4-H-4、5、6
1：NC
2：oe-YTHDC1
YTHDC1-4.5.6
SHOW
 1 1 1 2 2 2
M
 1 1 1 2 2 2
M
100 kD
YTHDC1
YTHDC1
70 kD
55 kD
40 kD
GAPDH
35 kD

## Slide 71
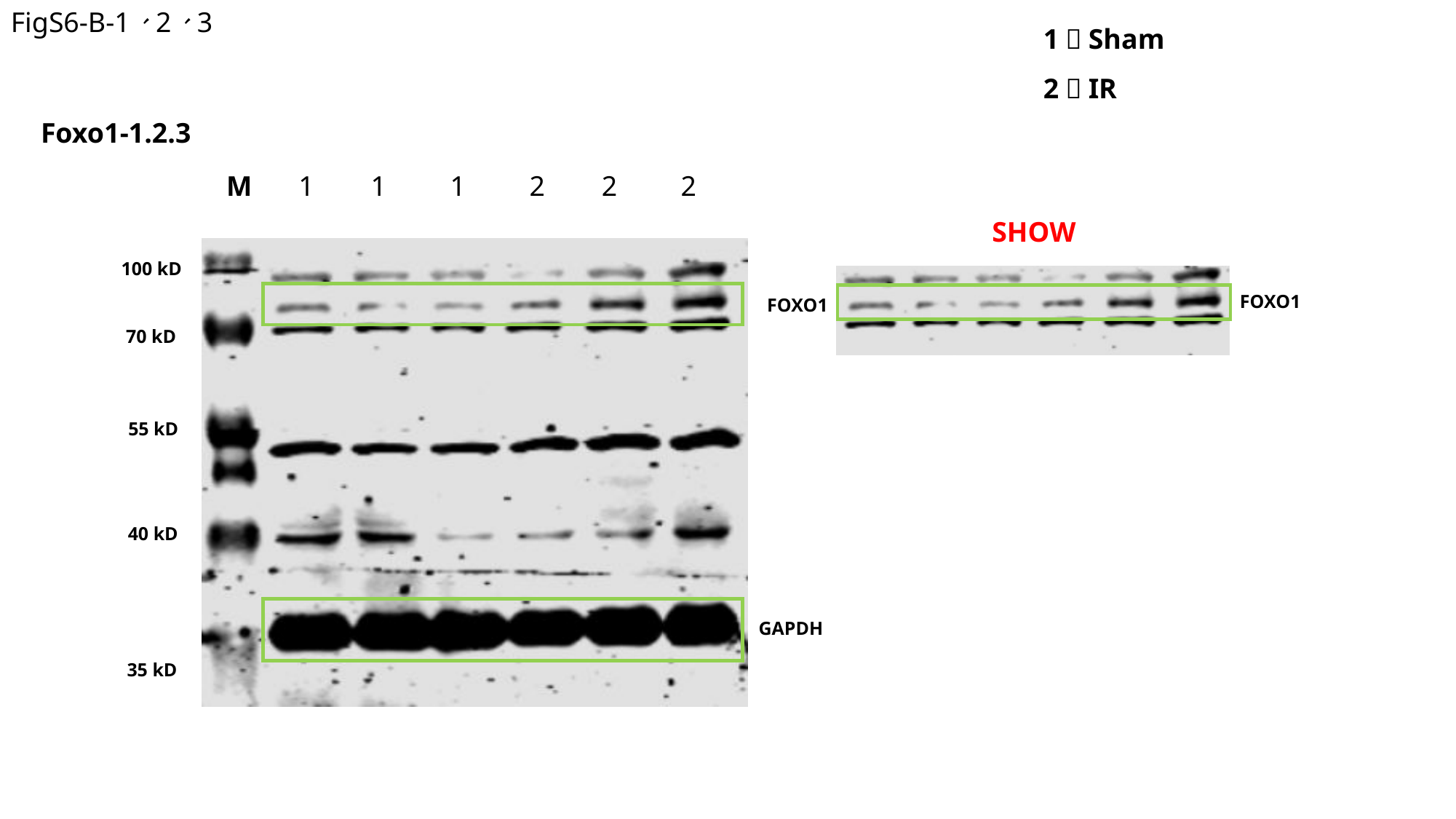

FigS6-B-1、2、3
1：Sham
2：IR
Foxo1-1.2.3
M
 1 1 1 2 2 2
SHOW
100 kD
FOXO1
FOXO1
70 kD
55 kD
40 kD
GAPDH
35 kD

## Slide 72
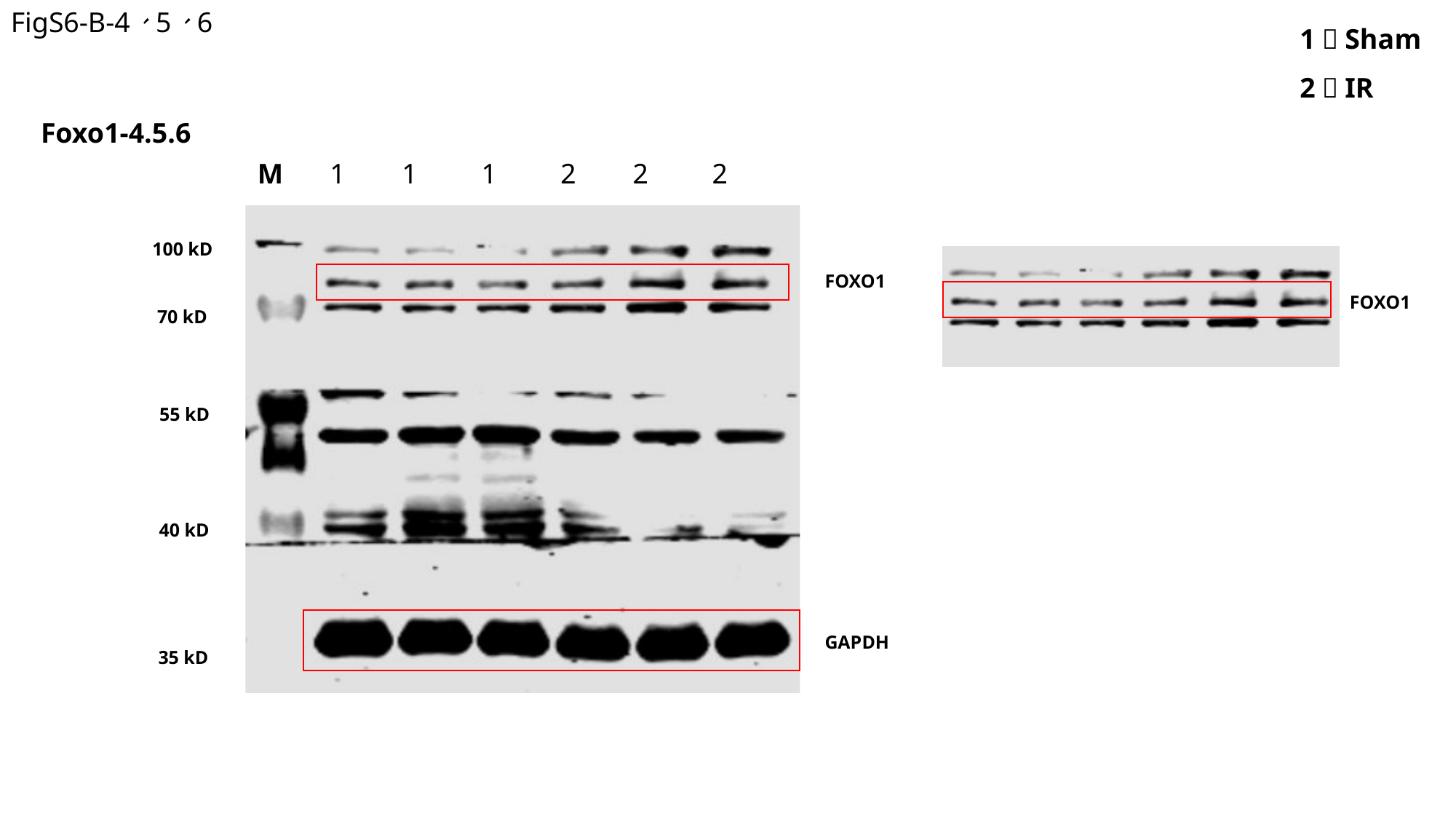

FigS6-B-4、5、6
1：Sham
2：IR
Foxo1-4.5.6
M
 1 1 1 2 2 2
100 kD
FOXO1
FOXO1
70 kD
55 kD
40 kD
GAPDH
35 kD

## Slide 73
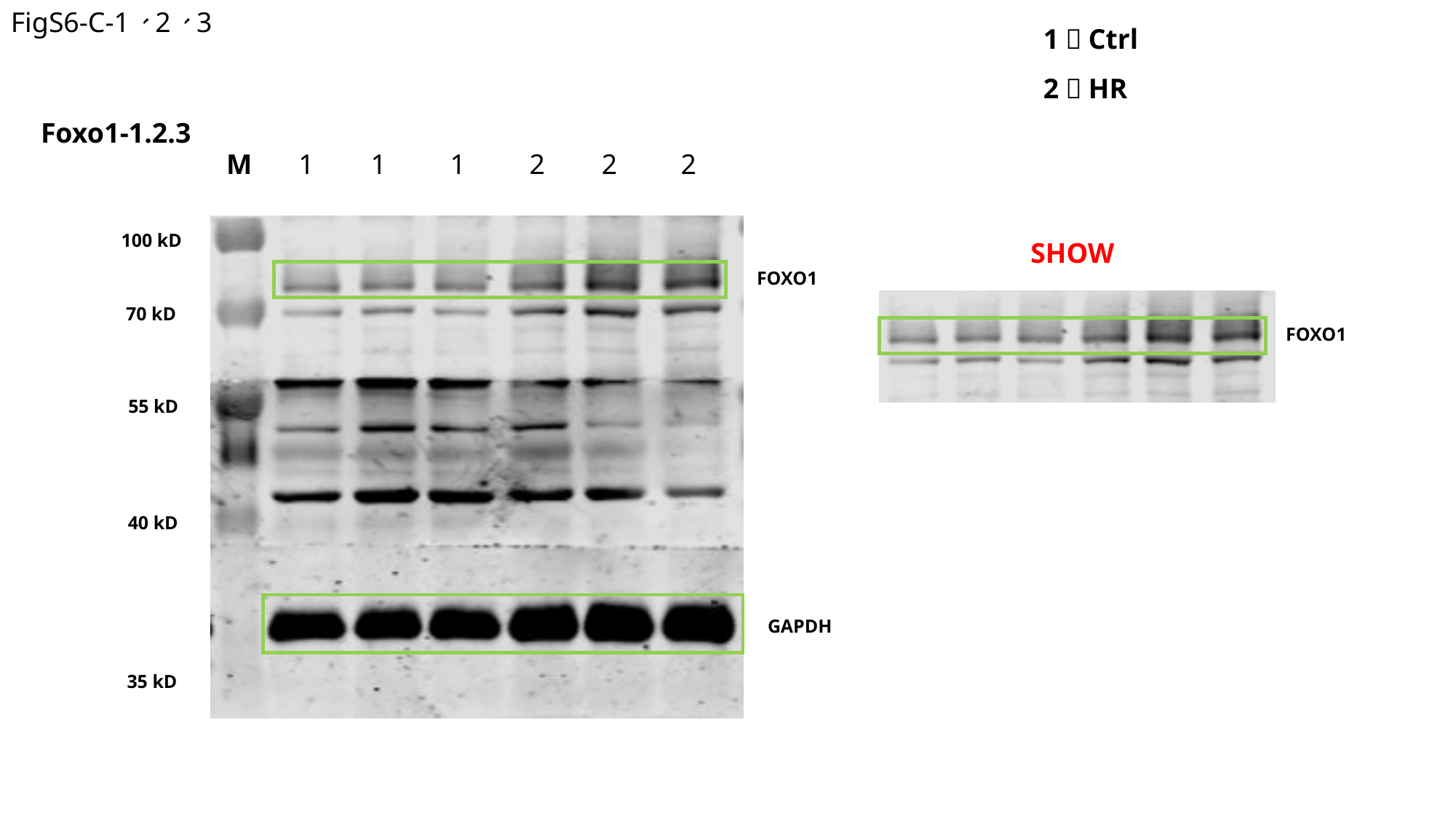

FigS6-C-1、2、3
1：Ctrl
2：HR
Foxo1-1.2.3
M
 1 1 1 2 2 2
100 kD
SHOW
FOXO1
70 kD
FOXO1
55 kD
40 kD
GAPDH
35 kD

## Slide 74
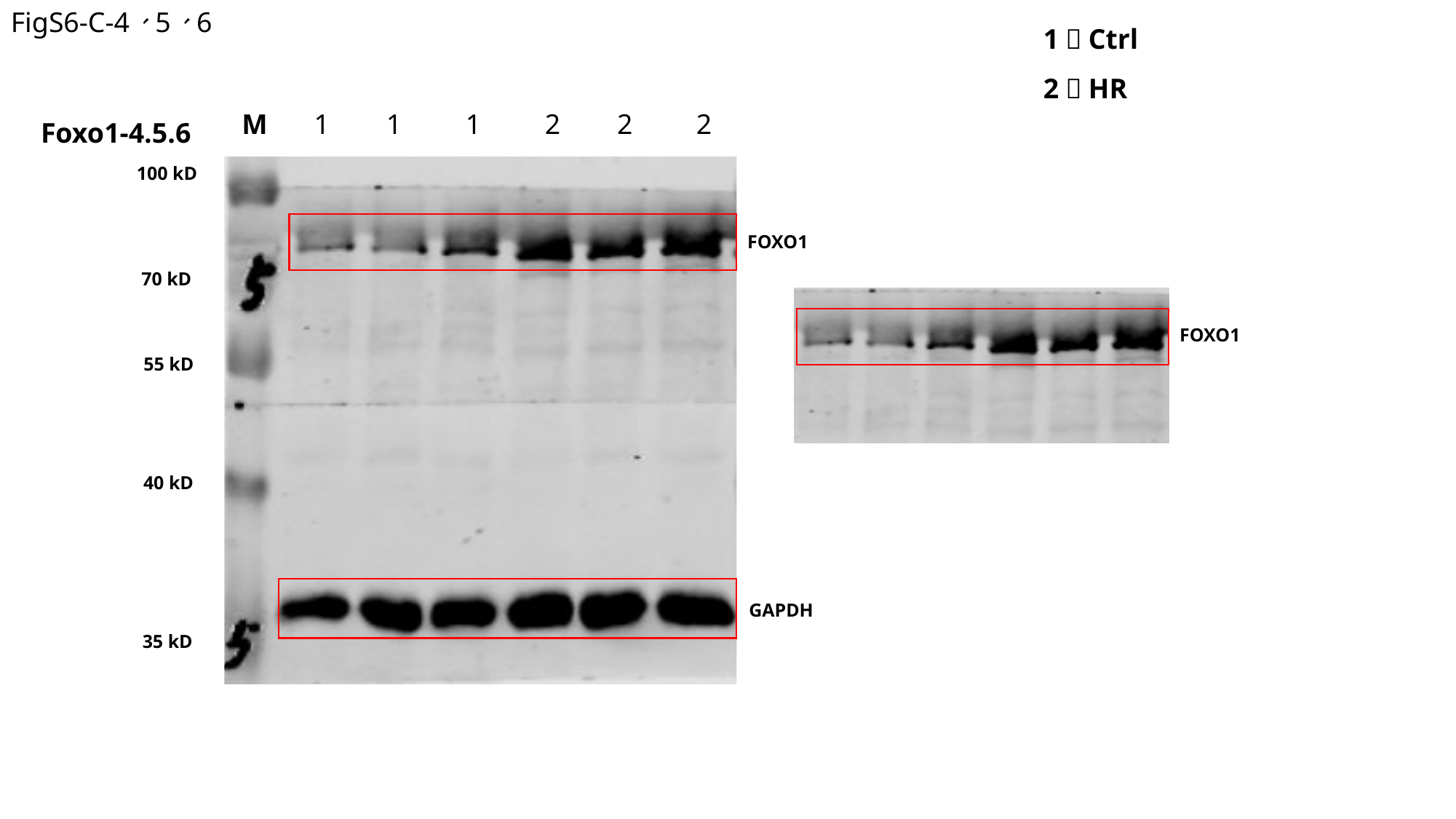

FigS6-C-4、5、6
1：Ctrl
2：HR
M
 1 1 1 2 2 2
Foxo1-4.5.6
100 kD
FOXO1
70 kD
FOXO1
55 kD
40 kD
GAPDH
35 kD

## Slide 75
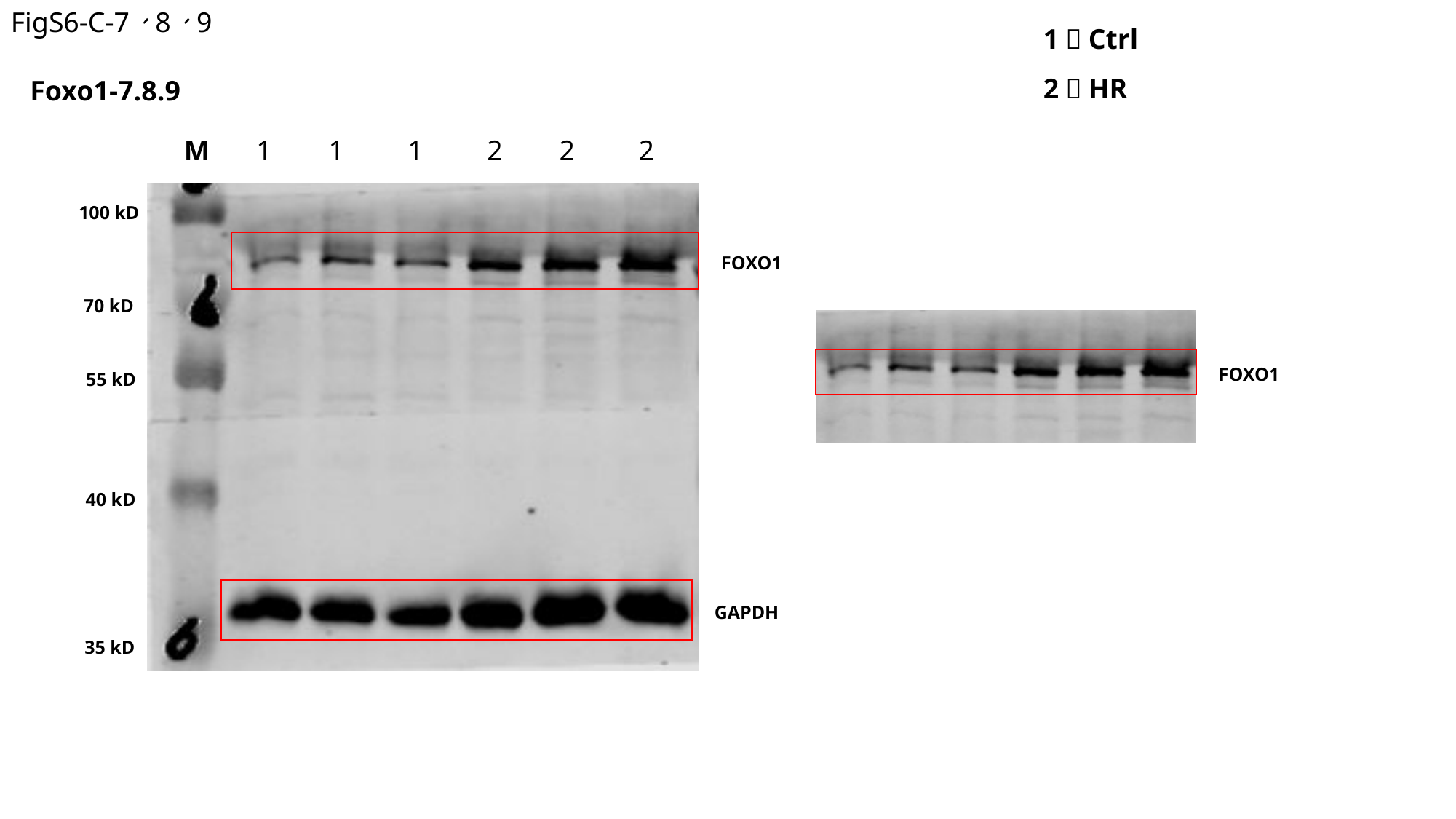

FigS6-C-7、8、9
1：Ctrl
2：HR
Foxo1-7.8.9
M
 1 1 1 2 2 2
100 kD
FOXO1
70 kD
FOXO1
55 kD
40 kD
GAPDH
35 kD

## Slide 76
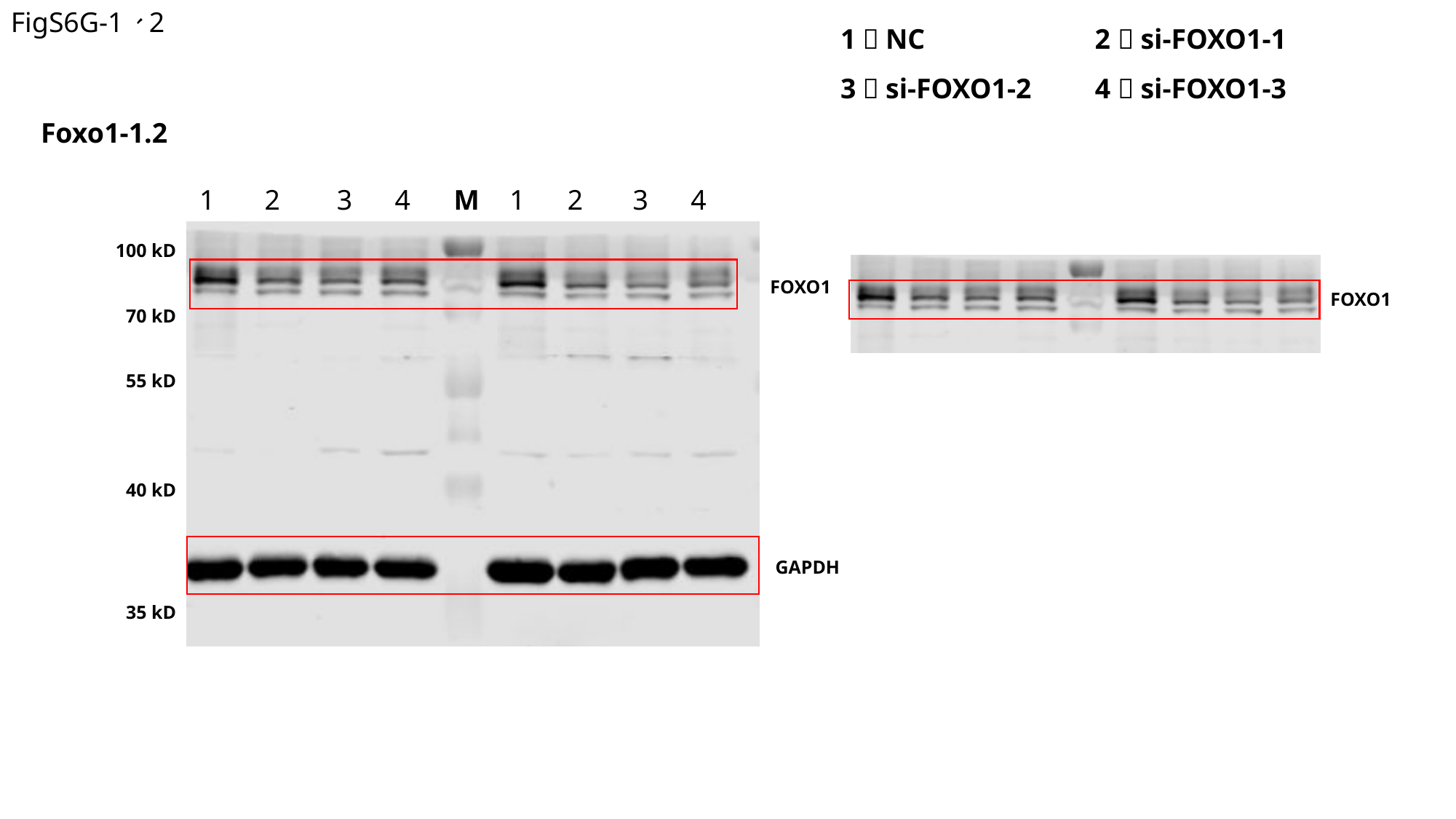

FigS6G-1、2
1：NC 2：si-FOXO1-1
3：si-FOXO1-2 4：si-FOXO1-3
Foxo1-1.2
 1 2 3 4 1 2 3 4
M
100 kD
FOXO1
FOXO1
70 kD
55 kD
40 kD
GAPDH
35 kD

## Slide 77
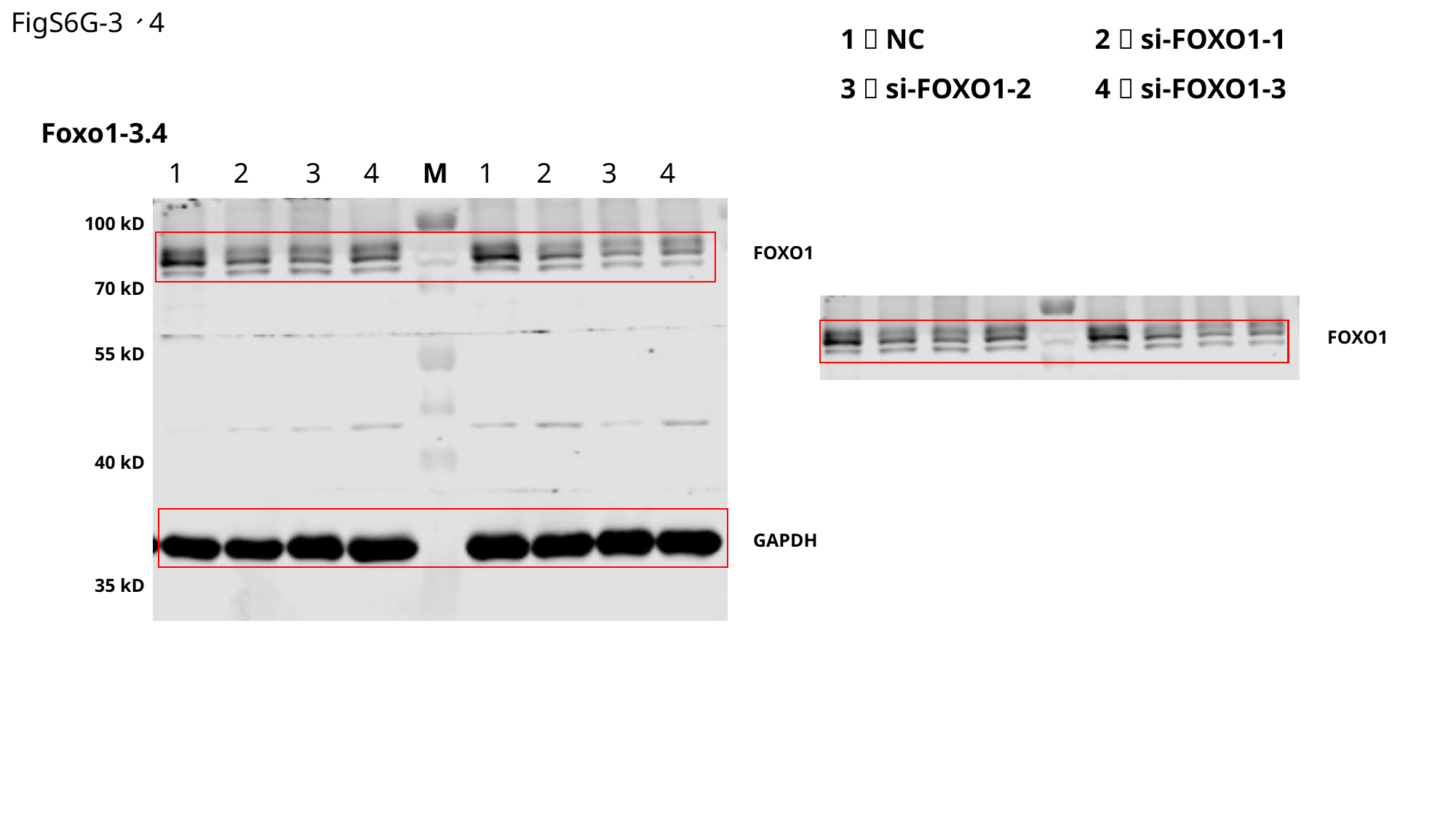

FigS6G-3、4
1：NC 2：si-FOXO1-1
3：si-FOXO1-2 4：si-FOXO1-3
Foxo1-3.4
 1 2 3 4 1 2 3 4
M
100 kD
FOXO1
70 kD
FOXO1
55 kD
40 kD
GAPDH
35 kD

## Slide 78
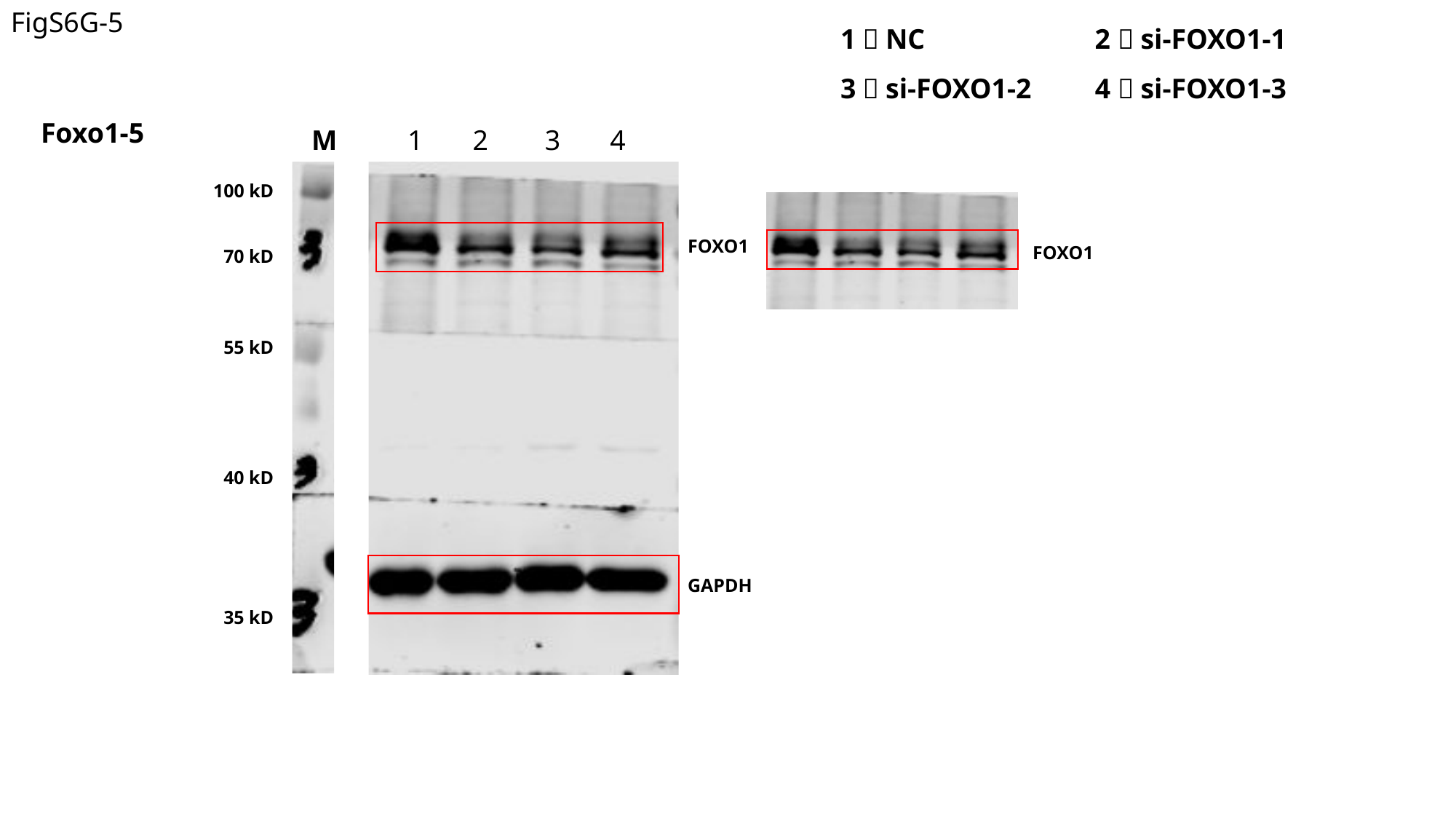

FigS6G-5
1：NC 2：si-FOXO1-1
3：si-FOXO1-2 4：si-FOXO1-3
Foxo1-5
M
 1 2 3 4
100 kD
FOXO1
FOXO1
70 kD
55 kD
40 kD
GAPDH
35 kD

## Slide 79
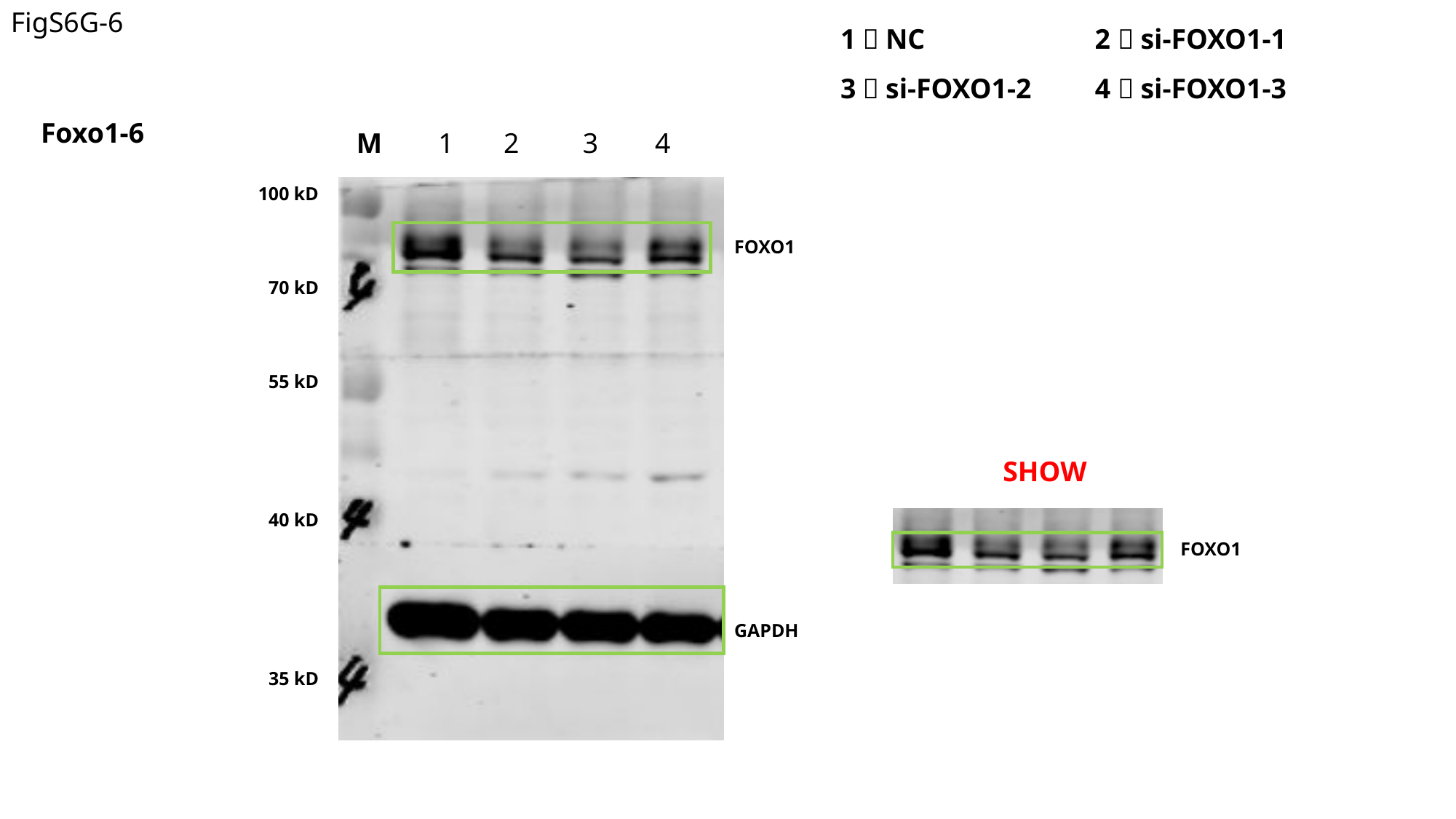

FigS6G-6
1：NC 2：si-FOXO1-1
3：si-FOXO1-2 4：si-FOXO1-3
Foxo1-6
M
 1 2 3 4
100 kD
FOXO1
70 kD
55 kD
SHOW
40 kD
FOXO1
GAPDH
35 kD

## Slide 80
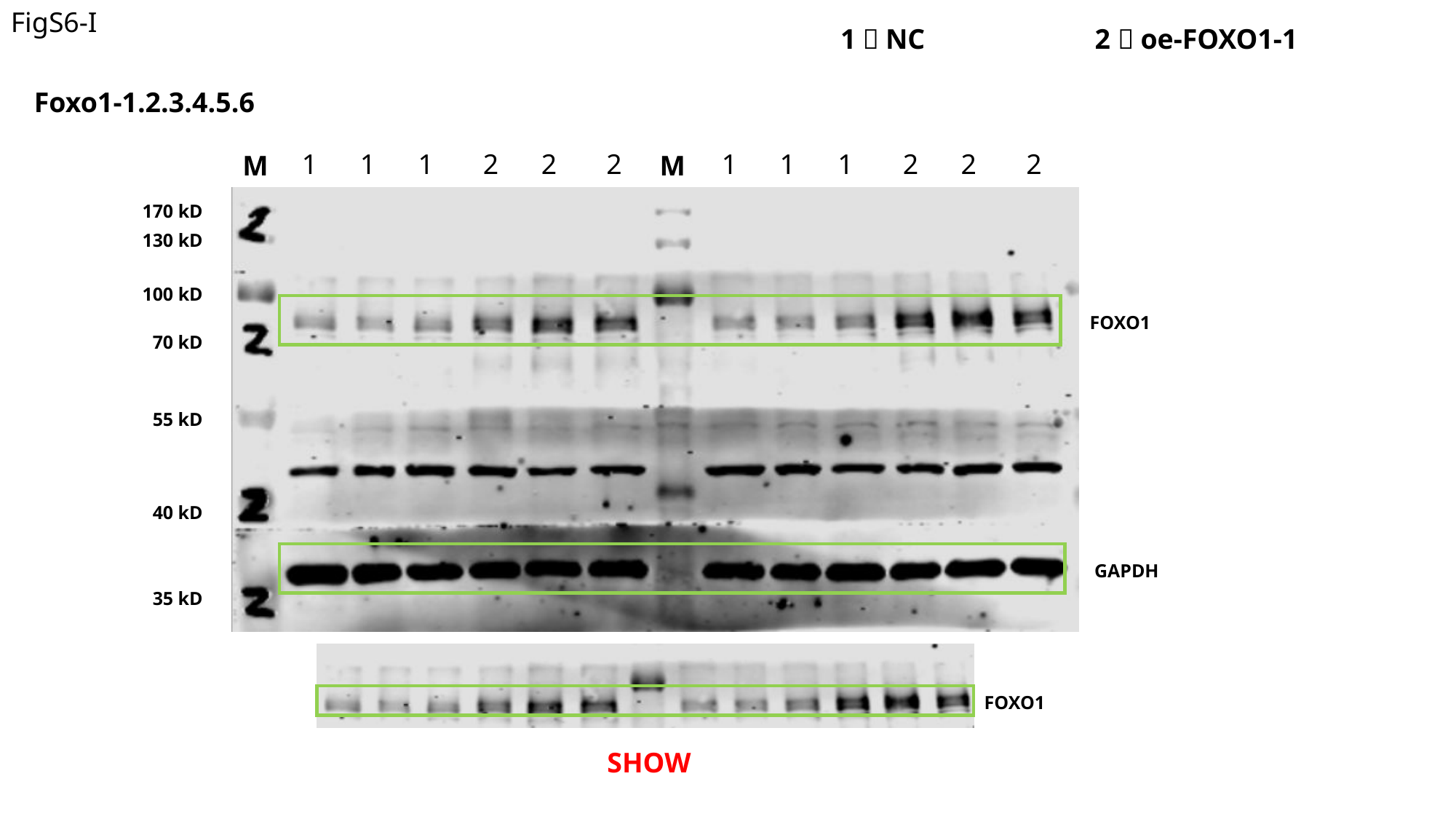

FigS6-I
1：NC 2：oe-FOXO1-1
Foxo1-1.2.3.4.5.6
 1 1 1 2 2 2
 1 1 1 2 2 2
M
M
170 kD
130 kD
100 kD
FOXO1
70 kD
55 kD
40 kD
GAPDH
35 kD
FOXO1
SHOW
